# Native characterization of nucleic acid motif thermodynamics via non-covalent catalysis

**DOI:** 10.1038/ncomms10319

**Published:** 2016-01-19

**Authors:** Chunyan Wang, Jin H. Bae, David Yu Zhang

**Affiliations:** 1 Department of Bioengineering, Rice University, Houston, Texas 77030, USA; 2 Systems, Synthetic, and Physical Biology, Rice University, Houston, Texas 77030, USA

## Abstract

DNA hybridization thermodynamics is critical for accurate design of oligonucleotides for biotechnology and nanotechnology applications, but parameters currently in use are inaccurately extrapolated based on limited quantitative understanding of thermal behaviours. Here, we present a method to measure the Δ*G*° of DNA motifs at temperatures and buffer conditions of interest, with significantly better accuracy (6- to 14-fold lower s.e.) than prior methods. The equilibrium constant of a reaction with thermodynamics closely approximating that of a desired motif is numerically calculated from directly observed reactant and product equilibrium concentrations; a DNA catalyst is designed to accelerate equilibration. We measured the Δ*G*° of terminal fluorophores, single-nucleotide dangles and multinucleotide dangles, in temperatures ranging from 10 to 45 °C.

## Abstract (web summary)

DNA hybridisation thermodynamics parameters underlie rational design of oligonucleotides for diagnostics and nanotechnology. Here, the authors present an accurate method to measure the free energy of a given DNA structure at specific temperature and buffer conditions.

## Graphical abstract

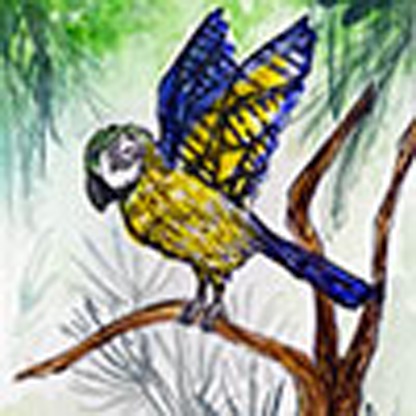

Nucleic acid biotechnology uses engineered oligonucleotides as therapeutic agents (for example, micro RNAs)^1^,^2^,^3^,^4^,^5^,^6^ and diagnostics reagents (for example, PCR primers and next generation sequencing capture probes)^7^,^8^,^9^,^10^,^11^,^12^. Nucleic acid nanotechnology uses engineered oligonucleotides as building blocks for constructing precisely patterned structures^13^,^14^,^15^,^16^,^17^ and complex spatio-temporal circuits^18^,^19^,^20^,^21^,^22^. In both fields, accurate rational design of oligonucleotides with intended thermodynamics properties is critical to achieving desired system function.

Errors in predicted Δ*G*° or melting temperature values result in undesirable non-specific interactions or unpredictable aggregation/assembly pathways. Currently, the best DNA design and folding software exhibit errors in predicted melting temperature of ∼1.5 °C (ref. 23), corresponding to errors of roughly 3 kcal mol^−1^. This indicates that there is significant inaccuracy even in common motifs such as DNA base stacks and dangles, an observation confirmed by chemical probing of nucleic acid folding structures^24^.

Current thermodynamic parameters are extrapolated from experiments in very different temperatures and buffer conditions than typically used (for example, melt curves in 1 M Na^+^; (refs 25, 26). In addition, thermodynamics parameters for individual motifs need to be inferred through linear algebra decomposition or principal component analysis, because each melt curve provides the aggregate Δ*H*° and Δ*S*° of many motifs. Large numbers of experiments on different sequences statistically mitigate the latter source of unbiased error, but the former results in systematic errors that cannot be reduced via melt curve experiments.

Here we present a generalized method for measuring Δ*G*° values of individual DNA motifs in native conditions of interest. A reaction is designed with Δ*G*° equivalent to the Δ*G*° of the motif of interest, and the equilibrium concentrations of the reactant and product species are measured to numerically calculate the equilibrium constant *K*_eq_. The innovation that enables this approach is a DNA catalyst system that accelerates forward and reverse rate constants by orders of magnitude, allowing rapid equilibration^27^,^28^.

We believe our native catalysis method can be used to provide an updated database of nucleic acid thermodynamic parameters for more accurate prediction and design software. We have started this process by providing Δ*G*° values for (1) 80 terminal fluorophore parameters, (2) 160 single-nucleotide dangle parameters and (3) 140 multinucleotide dangle parameters. Through the course of our studies, we discovered that multinucleotide dangles destabilize nearby DNA duplexes, asymptoting at +0.56 kcal mol^−1^ above a single-nucleotide dangle for dangles 8 nt or longer.

## Results

### Nucleic acid motif thermodynamics

**Figure 1:**
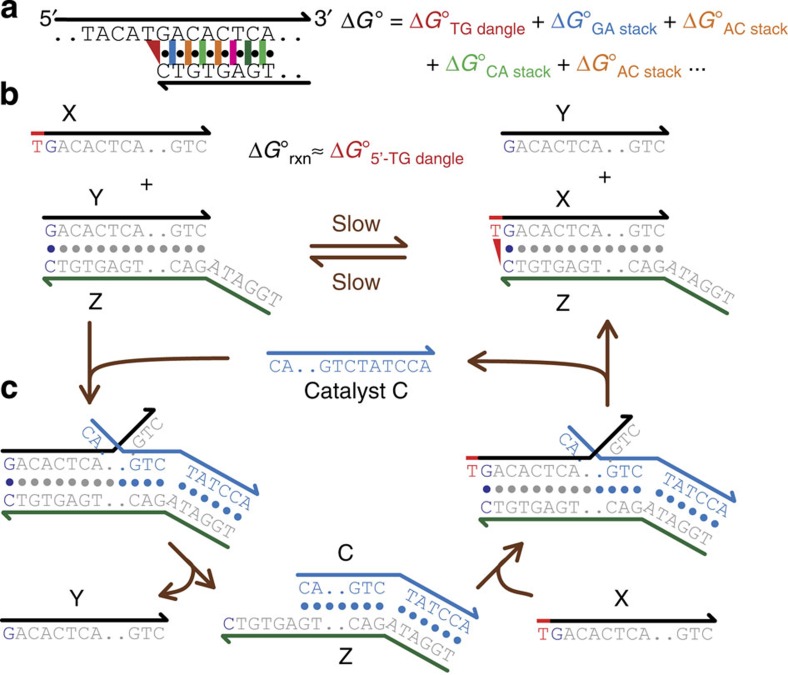
DNA hybridization thermodynamics. (**a**) The standard model of nucleic acid hybridization decomposes any nucleic acid molecule into a number of non-overlapping motifs (for example, dangles and stacks), and predicts the standard free energy of formation of the molecule to be the sum of the Δ*G*° of the individual motifs. Traditionally, motif Δ*G*° values are characterized using melt curves, and then extrapolated to other temperatures and buffer conditions. (**b**) In principle, motif Δ*G*° can be measured in native isothermal conditions by observing the equilibrium concentrations of an appropriately designed 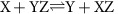 strand displacement reaction. For example, the shown reaction characterizes the Δ*G*° of the 5′-T dangle next to a closing G–C base pair; the partially double-stranded product differs from the partially double-stranded reactant by only the motif of interest. By designing the single-stranded reactants to possess negligible secondary structure, the reaction standard free energy 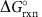 will closely approximate the dangle standard free energy 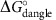. (**c**) A non-covalent strand displacement catalyst C can be designed to accelerate reaction equilibration to complete within minutes. The reverse reaction pathway is analogous to the forward reaction pathway shown here, and is also accelerated by the catalyst.

The standard model of nucleic acid hybridization thermodynamics^25^,^26^ is a local model, in which the structure of a nucleic acid molecule or complex is dissected into a number of non-overlapping motifs, such as base stacks, bulges and dangles. The standard free energy of formation of a molecule is calculated as the sum of its component motifs (Fig. 1a). In the past 30 years, the thermodynamics of many DNA and RNA motifs have been characterized^29^,^30^,^31^,^32^ to inform bioinformatics software for nucleic acid structure prediction^33^,^34^,^35^,^36^ and probe^37^,^38^,^39^ or primer^40^,^41^ or nanostructure^15^,^42^ design. However, inaccuracies in the Δ*G*° of DNA motifs are compounded in the overall Δ*G*° of hybridization, and lead to errors in predicted hybridization affinities.

Traditionally, motif thermodynamics (standard free energy of formation Δ*G*°) are characterized through melt curve analysis experiments^43^,^44^. These methods suffer from two major sources of inaccuracy: (1) yield and Δ*G*° measurement are inaccurate except near the melting temperature, and (2) linear algebra decomposition of motif thermodynamics reduces accuracy. Although recently melt curve analysis accuracy has been improved through the development of high-resolution melt (HRM; ref. 45), these limitations still exist. See Supplementary Note 1 for further discussion on melt curve inference of Δ*G*°. Other methods, such as isothermal titration calorimetry^46^, can be potentially used to provide higher resolution thermodynamic data, but are rarely used in practice because of the high nucleic acid concentration requirements necessitated by the small absolute values of Δ*H*°.

### Reaction construction

Our method starts with the construction of a reaction whose reaction standard free energy 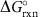 closely approximates the motif standard free energy 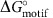 (Fig. 1b). This is achieved by designing a duplex product bearing the motif of interest (XZ) and a duplex reactant differing from the duplex product by only the motif (YZ). To balance the reaction, a single-stranded reactant oligonucleotide (X), which is part of the duplex product but not the duplex reactant, is added to the left side of the reaction. Similarly, a single-stranded product oligonucleotide (Y) is added to the right side of the reaction.

The net reaction is 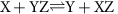, and


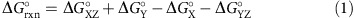



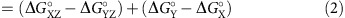


The standard free energy of the motif (in Fig. 1b, a 5′-TG dangle) can be expressed as 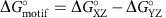 and is equal to 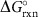 when 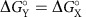. One way to ensure the latter is approximately true is to design the sequences of X and Y to avoid any predicted secondary structure.

The value of 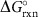 can be numerically computed from the observed equilibrium concentrations of the species:


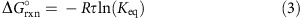



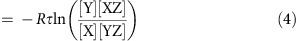


Here *R* is the universal gas constant (or Boltzmann constant), and 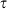 denotes the temperature in Kelvin. Given the initial concentrations [X]_0_, [Y]_0_, [XZ]_0_, and [YZ]_0_, all four equilibrium concentrations can be computed from any one equilibrium concentration based on simple algebra. Thus, we can accurately determine 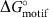 using an accurate measure of the equilibrium concentration of any one of the four species.

### Non-covalent catalysis

The above reaction, by itself, equilibrates slowly with a second order rate constant of roughly 1 M^−1^ s^−1^ (refs 28, 47, 48), corresponding to a half-life of roughly 100 days at 100 nM concentrations. It is not practical to wait so long for the reaction to naturally reach equilibrium because nucleotide oxidation (for example, adenine to inosine) occurs on a similar time scale^49^,^50^, and will bias the inferred thermodynamic results. To accelerate equilibration kinetics, we design a non-covalent strand displacement catalyst C, first introduced in ref. 27.

Figure 1c shows the catalysis reaction mechanism. C first binds to the single-stranded portion of Z in YZ, known as a toehold, and then displaces Y one nucleotide at a time through an unbiased random walk process known as branch migration. Because the elementary single-base replacement steps in branch migration are very fast (10–100 μs (ref. 51)), the entire displacement process occurs with a first order rate constant of about 1 s^−1^ (ref. 28). Thus, the reaction is typically rate limited by the second order toehold binding process, and is similar to hybridization kinetics for sufficiently long toeholds^28^. When all nucleotides in C are base paired to their complementary nucleotides in Z, Y can spontaneously dissociate, leaving intermediate CZ. Subsequently, CZ can react with either X or Y in a similar manner as YZ initially reacted with C, releasing C to catalyse other reactions (Fig. 1c). Thus, catalyst C enables a rapid pathway for both the forward and the reverse reactions of 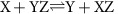 without changing the reaction's equilibrium constant (Supplementary Note 2).

### Fluorophore characterization

In this manuscript, we rely exclusively on fluorescent non-denaturing polyacrylamide gel electrophoresis (PAGE) to quantitate the equilibrium concentrations. Fluorescent PAGE shows very low background and high linearity (Supplementary Figs 5–8, Supplementary Note 4), allowing us to map band intensity to species concentration via a simple scaling constant. It also allows us to verify that the catalytic mechanism is functioning as intended, and that no long-lived side products or intermediates are being formed (such as CXZ or CYZ).

We first study the effects of terminal fluorophores on stabilizing DNA duplexes, both because these interactions have been relatively poorly characterized^52^,^53^ and because these are necessary prerequisites for our studies of other motifs. We chose to focus our fluorophore studies on the carboxy-X-rhodamine (ROX) and Alexa532 fluorophores, because our past experience indicates that these are temperature robust and their per unit fluorescence does not change significantly over a period of months when properly stored.

**Figure 2:**
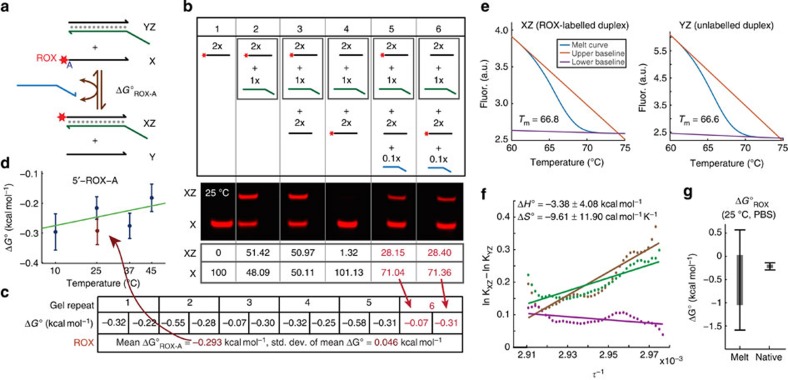
Experimental results. (**a**) Reaction constructed to measure the Δ*G*° of a 5′ ROX fluorophore next to an adjacent DNA duplex with a terminal A nucleotide 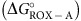. (**b**) Fluorescent PAGE demonstrating non-covalent catalysis. Relevant species were reacted at 25 °C in 12.5 mM Mg^2+^ buffer; 1x denotes ∼300 nM. Lanes 3 and 4 show significant difference in band intensity distribution, indicating that equilibrium is not achieved after 3 h of reaction in the absence of the catalyst. In contrast, lanes 5 and 6 show that a sub-stoichiometric (0.1x) amount of catalyst drives the reaction to equilibrium. Listed numbers below the gel are quantitated gel band intensities (arbitrary fluorescence units). (**c**) Summary of five additional experimental repeats. From the 12 inferred Δ*G*° values, we obtain mean 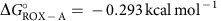. (**d**) Experiments to measure 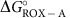 at 10, 25, 37 and 45 °C in PBS (blue), as well as in 12.5 mM Mg^2+^ (brown). The mean and mean s.d. of the Δ*G*° values are plotted against temperature; error bars show 1 s.d. The gels were always run at the same temperature as the hybridization reaction. The green line shows the best linear fit against the four sets of experiments in PBS, corresponding to Δ*H*°=−0.81 kcal mol^−1^ and Δ*S*°=−1.89 cal mol^−1^ K^−1^. (**e**) Melt curve of blunt duplex and 5′ ROX-labelled duplex in PBS buffer, based on fluorescence from the Syto82 intercalating dye (Supplementary Figs 70–72 and Supplementary Note 7). Upper and lower baselines are fitted to infer hybridization yields and equilibrium constants at different temperatures. (**f**) Van't Hoff plot of ln(*K*_eq_) against 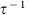 (Kelvin). (**g**) Melt curve analysis produced a Δ*G*° estimate with 14-fold higher s.d. than inferred using our native catalysis approach. Box shows 1 s.d. and error bars show 2 s.d.'s.

Figure 2a shows the net reaction used to characterize the thermodynamic effects of a 5′ ROX next to an adenine (A). We refer to the ROX-labelled species as the X species; species containing X (X and XZ) will appear as visible bands on a fluorescent PAGE, while all other bands will be invisible. Figure 2b shows the results from one fluorescent PAGE experiment characterizing 5′ ROX-A at 25 °C in 12.5 mM Mg^2+^. Gel band intensities were quantitated by software, and relative fluorescence units are listed below the gel; differences in pipetting volume and fluorophore quantum yield in ssDNA and dsDNA states resulted in different total fluorescences for different lanes; see Supplementary Fig. 14 and Supplementary Note 5 for discussion on quantitation and Δ*G*° calculation methodology.

Lanes 1 and 2 are controls showing the positions and intensities of the fluorescent XZ and X bands; species identities were verified with SYBR gold stained gels with a DNA ladder lane (Supplementary Fig. 19). In lane 3, X and Z are pre-mixed for 30 min and then Y is introduced and allowed to react with XZ for 3 h before PAGE. In lane 4, Y and Z are pre-mixed and X is subsequently introduced. The band distribution in lane 3 closely resembles that of lane 2, and the band distribution in lane 4 closely resembles lane 1, despite the fact that lanes 3 and 4 have the same composition of strands; this indicates that equilibrium is far from reached after 3 h of reaction. Lanes 5 and 6 are similar to lanes 3 and 4, except 0.1x of the catalyst C is introduced into each reaction after all other species are introduced, but before the 3 h incubation. The band distribution in lanes 5 and 6 are quantitatively similar to each other and intermediate between lanes 3 and 4, indicating that equilibrium has been achieved.

Lack of a higher molecular weight CXZ band indicates that CXZ and CYZ are short-lived and low in concentration at equilibrium. Under the assumption that CZ is likewise short-lived and insignificant in concentration (Supplementary Fig. 16), the equilibrium concentrations [Y] and [YZ] can be calculated given the initial concentrations [X]_0_, [Y]_0_, [XZ]_0_ and [YZ]_0_ from either [X] or [XZ]:


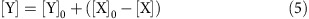



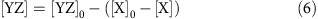



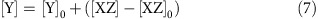



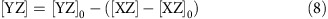


In a perfectly run reaction, the calculated equilibrium concentration [Y] should be the same regardless of whether it is calculated from [X] or [XZ], but in practice slight pipetting errors in the total amount of sample loaded per gel lane will lead to different values. A similar argument applies to [YZ] inference. We correct for these in our mathematical analysis and calculation of *K*_eq_ and Δ*G*° from gel band intensities (Supplementary Figs 16 and 21).

From the intensities of the gel bands in lanes 1, 2, 5 and 6, we calculate 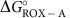 to be −0.07 kcal mol^−1^ from lane 5 and −0.31 kcal mol^−1^ from lane 6. Five additional fluorescent PAGE experiments were run under the same conditions using separately prepared samples, to mitigate the effects of pipetting error in any one experiment, and the inferred Δ*G*° values are shown in Fig. 2c. These 12 independent Δ*G*° values are then combined into a single mean 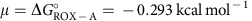, corresponding to our best estimate of the true value of the parameter from our experiments. The s.d. of the mean is calculated assuming Gaussian distributed error in the individual Δ*G*° values, and 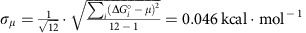.

In the course of calculating Δ*G*° values from gel band intensities, the initial concentrations [X]_0_, [Y]_0_, [XZ]_0_ and [YZ]_0_ are extremely important, and we found that conventional methods based on absorbance and extinction coefficient at 260 nm were insufficiently accurate. Instead, we use stoichiometry PAGE gels to determine the relative concentrations of X or Y to Z (Supplementary Fig. 9 and Supplementary Note 3). Our method systematically overestimates Δ*G*° values by ignoring the presence of the CZ, CXZ and CYZ intermediate species. However, because the amount of catalyst C is only 0.1x, the overestimation is bounded by 0.2 kcal mol^−1^ in an absolute worst case.

### Temperature dependence

In addition to 25 °C in 12.5 mM Mg^2+^, we also performed similar experiments (5–8 gels each) for the ROX-A fluorophore at temperatures of 10, 25, 37 and 45 °C in PBS (Sigma P5493, 0.01 M phosphate; 0.154 M NaCl; pH 7.4). The gels were always run at the same temperature as the hybridization reaction. Although the gel running buffer (TAE with no added salt) differs from the hybridization buffer, our control experiments (Supplementary Fig. 18) shows no significant difference in inferred Δ*G*° due to the transient change in buffer conditions before the start of the electrophoresis. The mean and mean s.d.'s of 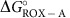 in each of these conditions are plotted against temperature in Fig. 2d. With the four separate series of experiments performed in PBS at different temperatures, we can use the relation 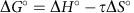 to infer Δ*H*° and Δ*S*° of the 5′-ROX-A using a linear fit.

Because each of the four mean Δ*G*° values at different temperatures has a different level of uncertainty (mean s.d.), it is inappropriate to use a standard least-squares fit. Instead, we obtain the best fit Δ*H*° and Δ*S*° values from a maximal likelihood standpoint, minimizing the sum of square *z* scores. The *z* score is calculated as:


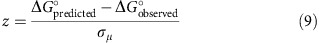


where 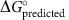 is the value calculated as 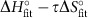, 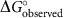 is the mean experimental Δ*G*° at the relevant temperature, and *σ*_*μ*_ is the mean s.d. The green line in Fig. 2d shows the best fit Δ*H*°=−0.81 kcal mol^−1^ and Δ*S*°=−1.89 cal mol^−1^ *K*^−1^. See Supplementary Fig. 17 for details on Δ*H*° and Δ*S*° fitting, including calculated of s.d.'s on best fit Δ*H*° and Δ*S*° values.

Our best fit linear regression to our measured Δ*G*° values often showed poor quality of fit, resulting in large s.d.'s in our inferred values of Δ*H*° and Δ*S*°. One possible explanation could be the temperature dependence of motif Δ*H*° and Δ*S*° values. Although the standard model of DNA hybridization^25^ assumes temperature invariance of Δ*H*° and Δ*S*°, there is debate within the literature as to whether this is true for all hybridization motifs^54^,^55^,^56^,^57^,^58^.

### Comparison to melt curve analysis

To compare the quality of the thermodynamics parameters produced by our native catalysis against the standard-of-practice melt curve analysis approach, we also performed HRM analysis of the ROX-labelled XZ and unlabelled YZ molecules (Fig. 2e). Hybridization yields of the XZ and YZ molecules are inferred from the melt curves, and used to inform the numerical calculation of the equilibrium constants *K*_XZ_ and *K*_YZ_, of the 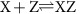 and 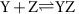 reactions, respectively. The difference in the logarithms of *K*_XZ_ and *K*_YZ_ corresponds to the equilibrium constant of the ROX-duplex motif, and is plotted against the inverse of the temperature in Kelvin in a van't Hoff plot. The best linear fits to the van't Hoff plot provides Δ*H*° and Δ*S*° values, from which Δ*G*° at a particular temperature can be calculated.

Triplicate runs of the melt curves of XZ and YZ gave relatively low quantitative agreement in the van't Hoff plot, due to the small changes in melting temperature and thermodynamics involved (Fig. 2f). The Δ*G*° value of the ROX-duplex interaction at 25 °C in PBS, based on melt curve parameters, is predicted with a mean of −0.51 kcal mol^−1^ with a mean s.d. of 0.54 kcal mol^−1^. In contrast, our native catalysis method produces Δ*G*°=−0.22 kcal mol^−1^, with a mean s.d. of 0.038 kcal mol^−1^. Although unbiased errors due to repeat experiments can be attenuated via statistically large sampling, the 14-fold higher s.d. of parameters produced by melt curves would necessitate 196-fold more experiments, on average, to generate the same mean s.d. as our native catalysis technique.

### Single-nucleotide dangles

**Figure 3:**
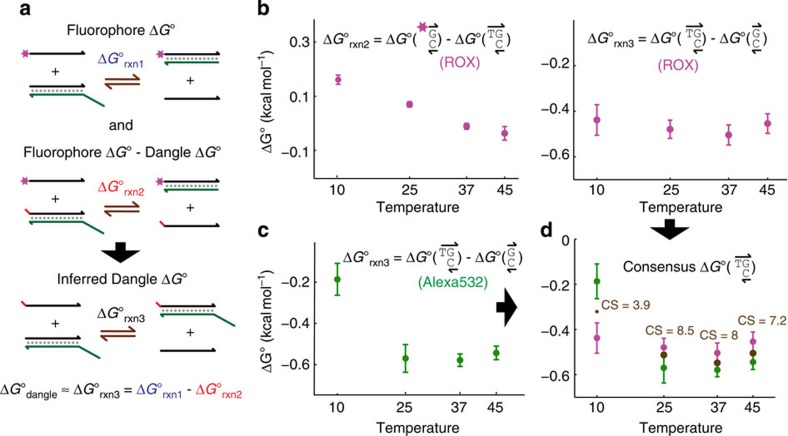
Dangle Δ*G*° characterization. (**a**) To accurately measure dangle Δ*G*° using fluorescent PAGE, we design a second reaction with reaction standard free energy 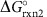 reflecting the effects of both the dangle of interest and the fluorophore. By numerically subtracting 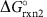 from 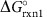, we calculate the value of 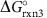 corresponding to the dangle. (**b**) Summary of mean and mean s.d. 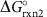 based on ROX, and the corresponding 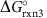 after subtracting from the relevant 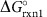 values from Fig. 2d. (**c**) 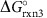 values inferred using the Alexa532 fluorophore, rather than the ROX fluorophore; see Supplementary Table 4 for data on Alexa532-based measurements. (**d**) Consensus dangle Δ*G*° values are calculated as the maximal likelihood Δ*G*° value based on the independent readings from ROX and Alexa532 experiments. The CS represents 10 minus the sum of the squares of z score of the consensus Δ*G*° relative to the mean Δ*G*° values measured from ROX and Alexa532; a CS of eight indicates that the consensus Δ*G*° is within 1 s.d. of each mean Δ*G*°. See Supplementary Table 6 for summary results of all 32 single-nucleotide dangles.

Although it is possible to construct a similar catalysed 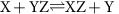 reaction to directly measure dangle motif thermodynamics, it would be difficult to accurately quantitate the concentrations of any of the species, because X and Y would have very similar mobility, and likewise with XZ and YZ. Consequently, we chose to indirectly characterize the dangle motifs by measuring the relative Δ*G*° of a dangle compared with a fluorophore (reaction 2 in Fig. 3a), and subtracting that value from the Δ*G*° of the fluorophore that we obtained earlier in Fig. 2 and related experiments. The resulting 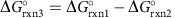 corresponds to the net reaction querying only the dangle, and closely approximates 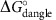 that we ultimately wish to characterize. As a further benefit, because both 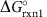 and 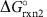 are overestimated by a similar amount due to the ignorance of the CZ, CYZ and CXZ species, 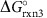 becomes unbiased due to the cancellation of the bias terms. The disadvantage of such a 2-reaction approach is the increased s.d. on the Δ*G*° parameters from subtracting two distributions.

The left panel of Fig. 3b shows the summarized mean 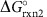 values at different temperatures in PBS based on a number of fluorescent PAGE experiments similar to that shown in Fig. 2b and the corresponding mean s.d.'s. The right panel shows the mean 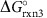 values obtained by subtracting the 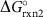 values in the left panel from the 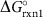 values shown in Fig. 2d. The mean s.d.'s of 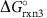 are calculated assuming that errors in 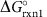 and 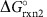 are independent, so that variance is additive:


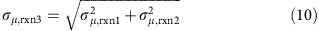


Figure 3c shows the mean and mean s.d. of 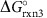 for the same 5′-TG dangle assayed using the Alexa532 fluorophore, and Fig. 3d shows the overlay of the two sets of values. The thermodynamics of Alexa532 was likewise characterized for a variety of nearest neighbour bases and temperatures (Supplementary Table 4). The *consensus* Δ*G*° value that we attribute to the dangle at each temperature is calculated as the maximum likelihood value based on minimizing the sum of square z scores. Mathematically,


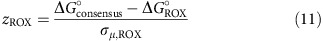



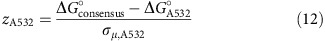



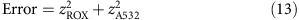


As a more intuitive metric of the agreement between the ROX and Alexa532, we define consensus score (CS) as:


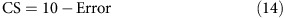


The CS is a measure of how much two independently ordered sets of strands, using two distinct fluorophores, agree with each other in terms of inferred Δ*G*° parameters, out of a maximum score of 10. CS ≥8 indicates that the consensus Δ*G*° is within 1 s.d. of both the ROX- and the Alexa-based Δ*G*° values, and CS ≥0 indicates that the consensus Δ*G*° is within 2.24 s.d.'s.

Table 1 shows consensus Δ*G*° values of all 32 single-nucleotide dangles at 25 and 37 °C in PBS, and at 25 °C in 12.5 mM Mg^2+^. Also shown is the corresponding s.d.'s and CS for each Δ*G*° parameter. Additional parameters are available for similar experiments performed at 10 and 45 °C in PBS and best fit Δ*H*° and Δ*S*° values (Supplementary Table 7). Using measured Δ*G*° values in PBS at four different temperatures, we also were able to calculate best fit values of Δ*H*° and Δ*S*° for the dangle motifs explored. As with fluorophore Δ*H*° and Δ*S*° parameters, however, we caution the reader against too much reliance on these values, due to the poor quality of linear fits, and the possibility of temperature-dependent Δ*H*° and Δ*S*° values^54^,^55^,^56^,^57^,^58^.

### Error analysis

In the course of our experiments for the previous two subsections on characterizing fluorophore and single-nucleotide dangle Δ*G*° values, we performed at least five fluorescent PAGE gels for each fluorophore parameter (corresponding to 10 independent Δ*G*° measurements) and at least two fluorescent PAGE gels for each fluorophore-dangle parameter. These repeats were done to reduce the mean s.d. on Δ*G*° values.

**Figure 4:**
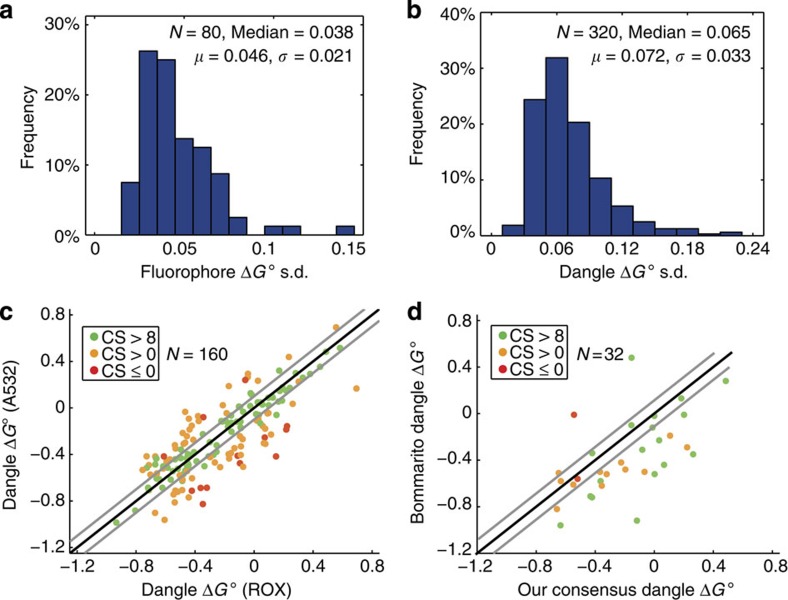
Error analysis of native characterization methodology. (**a**) Distribution of mean s.d.'s of a fluorophore next to a duplex (Fig. 2). All 80 fluorophore Δ*G*° parameters we obtained have mean s.d. below 0.15 kcal mol^−1^. (**b**) Distribution of mean s.d.'s of single-nucleotide dangles (Fig. 3). All 320 single-base dangle parameters have mean s.d. below 0.25 kcal mol^−1^. Because dangle Δ*G*° values were obtained by subtracting two Δ*G*° values each with their own independent s.d.'s, the s.d.'s are larger than those in **a**. (**c**) Comparison of dangle Δ*G*° values obtained via ROX and via Alexa532. Black line shows equality and grey lines show ±0.1 kcal mol^−1^ deviation. Of the 160 parameter pairs, 80 (50.0%) have CS >8, 66 (41.25%) have CS between 0 and 8, and 14 (8.75%) have CS <0. (**d**) Comparison of our consensus dangle Δ*G*° values with literature values by Bommarito *et al.* 59

Figure 4a plots as a histogram the distribution of the mean s.d.'s of Δ*G*° values for fluorophore interactions. *N*=80 corresponds to the number of different Δ*G*° parameters measured: (2 fluorophores; 5′ or 3′; 4 nearest neighbour nucleotides; 5 temperature/buffer conditions). These were collected from 429 separate fluorescent PAGE gels corresponding to 858 individual Δ*G*° values measured. All 80 dangle parameters' mean s.d.'s were below 0.15 kcal mol^−1^, with a mean of 0.046 kcal mol^−1^ and a s.d. (on the mean s.d.) of 0.021 kcal mol^−1^.

Similarly, Fig. 4b plots as a histogram the distribution of the mean s.d.'s of the Δ*G*° values for *N*=320 single-nucleotide dangle parameters: (2 fluorophores; 5′ or 3′; 4 dangle nucleotides; 4 nearest neighbour nucleotides; 5 temperature/buffer conditions). These were collected from 723 separate fluorescent PAGE gels corresponding to 1446 individual Δ*G*° values measured. The mean and s.d. of the parameter s.d.'s here were 0.072 kcal mol^−1^ and 0.033 kcal mol^−1^, respectively, and all parameter s.d.'s were below 0.25 kcal mol^−1^. These parameter s.d. values are larger than those in Fig. 4a both because they include the variance from fluorophores and because of the relatively fewer independent Δ*G*° measurements per parameter (average 4.52 per dangle parameter, compared with average 10.73 per fluorophore parameter).

Figure 4c plots the dangle Δ*G*° parameters measured using the Alexa532 against the same parameters measured using ROX. The Alexa532- and ROX-based measurements of the dangle motif Δ*G*° represent independent experiments and assays, agreement between the final dangle Δ*G*° values (high CS) indicates high confidence in our methodology and consensus Δ*G*° value. Parameters with CS>8 generally have no >0.1 kcal mol^−1^ deviation between the ROX and Alexa532 measurements.

We observed 14 parameters (out of 160) with CS≤0 (Supplementary Table S9-1), whereas a Gaussian distribution of errors would typically result in 4 out of 160 parameters (2.5%) possessing CS≤0. Except one outlier at −37.1, CS values ranged between −8.7 and 10.0, with 91% of values being positive and 50% of values being 8 or higher. Parameters with low CS likely imply that one or more assumptions our methodologies required were incorrect for these parameters. For example, it is possible that the equilibrium concentrations of CZ are different for the corresponding experiments using the two different fluorophores, resulting in bias in one or both Δ*G*° values. We believe that the consensus parameters with CS≥8 are likely to be objectively correct, because while any of a number of problems could cause the mean Δ*G*° measured from Alexa532 to differ from that from ROX, it is unlikely for these values to be coincidentally similar to within 0.1 kcal mol^−1^ while being wrong.

Figure 4d plots the literature single-nucleotide dangle parameters by Bommarito *et al.*^59^ against our consensus single-nucleotide dangle parameters at 37 °C. There is relatively little agreement between our values and Bommarito's values; only 9 out of 32 dangle parameters agreed to within 0.1 kcal mol^−1^. The mean absolute error between our consensus values and Bommarito's values was 0.269 kcal mol^−1^, and the root mean square error between them was 0.333 kcal mol^−1^.

Furthermore, there is little correlation between our parameters' CS and their agreement with Bommarito's values. Our consensus 5′ 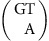 Δ*G*° has CS=9.0, and is 0.601 kcal mol^−1^ below Bommarito's value, while our consensus 3′ 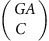 Δ*G*° has CS=10.0, and is 0.802 kcal mol^−1^ above Bommarito's value. Because our method offers a more direct measure of dangle Δ*G*° parameters, compared to Bommarito's melt curve methods that had to deconvolute the thermodynamic contributions of many base stacks from that of dangles, we generally believe our parameters to be more accurate.

Another contribution to the discrepancy may be the difference in the ‘core' sequences (for example, all nucleotides except the dangle and nearest neighbour, in our case) used for the two papers. The standard nearest neighbour model assumes that the identities of distal bases should have no effect on the motif thermodynamics, but both our own analyses (not shown) and experimental studies on coaxial dangles^60^ suggest that this simplifying assumption may not be true.

There is also a systematic bias in comparing the two sets of dangle parameters; on average, our consensus dangles parameters were 0.190 kcal mol^−1^ more positive. One possible contributing factor to the systematic bias is that Bommarito's experiments were performed in 1 M Na^+^, whereas our experiments were in the physiological PBS (0.15 M Na^+^) buffer. The standard model of DNA hybridization assumes that dangles Δ*G*° contributions are not affected by salinity, but we believe this may be incorrect.

### Multinucleotide dangles

We next applied our native motif characterization method to the study of multinucleotide dangles. The standard model of DNA hybridization used by most commonly used software^33^,^36^,^40^ considers only the effects of the first dangle nucleotide. Melt studies on multinucleotide dangles^61^,^62^,^63^,^64^ reported inconsistent findings, with some reporting stabilizing effects of long dangles and others reporting destabilizing effects. As far as we are aware, there have not been any systematic studies on the effects of dangle length on Δ*G*°.

**Figure 5:**
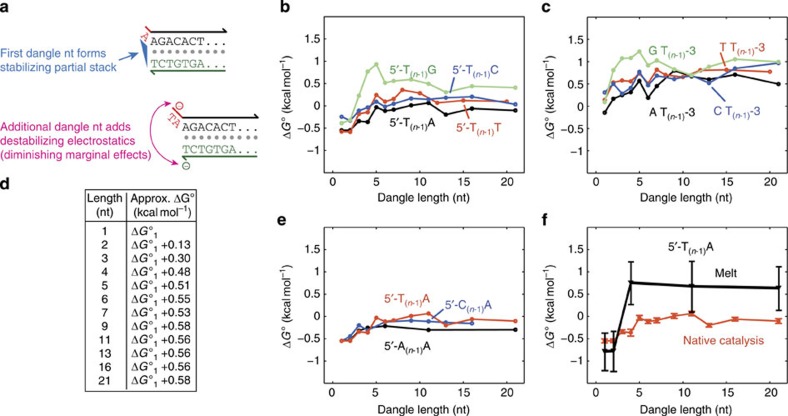
Thermodynamics of multinucleotide dangles. (**a**) The standard model of DNA hybridization considers only the effects of the first dangle nucleotide, which is known to potentially form a partial stack with the last base pair of the adjoining helix. From a biophysics point of view, additional nucleotides of dangle likely contribute a destabilizing effect due to the electrostatic repulsion from the additional dangle bases and the nucleotides on the complementary strand. (**b**) Consensus Δ*G*° for 5′ multinucleotide dangles of different lengths at 25 °C in PBS. Each plotted data point represents the consensus value from experiments using ROX and using Alexa532, as in Fig. 3. (**c**) Consensus Δ*G*° for 3′ multinucleotide dangles of different lengths at 25 °C in PBS. (**d**) Approximate Δ*G*° of multinucleotide dangles based on length and Δ*G*° of the single-nucleotide dangle. (**e**) Consensus Δ*G*° for 5′ multinucleotide dangles of different lengths at 25 °C in PBS. The first dangle base was fixed to be A; the remainder were homopolymers of A, T, or C. (**f**) Comparison of multinucleotide dangle thermodynamics based on native catalysis versus melt curves. Identical buffers and oligonucleotides were used for the two sets of experiments. Inferred parameters are consistent with each other to within 2 s.d.'s, but melt curve-based parameters show 6- to 12-fold higher errors than the native catalysis method.

From a theoretical point of view, we believe that the first dangle nucleotide contributes two opposing effects on the stability of nearby duplexes (Fig. 5a). First, it can form a partial base stack with the terminal base pair, adding stability by increasing the interactions between the aromatic groups in the nucleoside bases^65^. Second, the negatively charged phosphodiester backbone is brought in close proximity to the negatively charged phosphodiester backbone of the complementary strand, reducing stability by increasing electrostatic repulsion. In buffers such as PBS or 12.5 mM Mg^2+^, the distances involved (0.43 nm for the dangle plus 2.36 nm for the B-DNA helix) are comparable to the Debye length (estimated to be roughly 2 nm (ref. 66)).

Except in the case where the dangle forms significant secondary structure, additional dangle nucleotides past the first do not contribute further base stacking, but continue to increase electrostatic repulsion, albeit with diminishing impact due to increasing distances involved. Consequently, we hypothesized that duplexes with longer dangles will be destabilized relative to single-nucleotide dangles. To verify our hypothesis, we measured 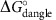 for multinucleotide poly-T dangles with dangle length between 2 and 21. We chose to use poly-T, poly-A and poly-C multinucleotide dangles to ensure that there would be no secondary structure in the dangle. Because the first dangle nucleotide also contributes the partial base stack, we performed experiments for all four nucleotides as the first dangle nucleotide. We did not do any experiments on multinucleotide dangles with non-homogeneous sequence, because the secondary structure of dangles would have introduced additional complicating thermodynamics terms.

Figure 5b shows the consensus Δ*G*° values we measured for 5′ multinucleotide dangles, based on independent experiments with ROX and with Alexa532. For poly-T dangles of >8 nt, the value of Δ*G*° appears to asymptotically approach +0.56 kcal mol^−1^ above that of the single-nucleotide dangle Δ*G*°. Figure 5c shows the consensus Δ*G*° values we measured for 3′ multinucleotide dangles; all values follow similar trend versus dangle length, although all Δ*G*° values are more positive due to the weaker stabilizing effect of the first dangle nucleotide closest to the duplex. Figure 5d shows the averaged, smoothed value of multinucleotide dangle Δ*G*° compared with single-base dangle Δ*G*°, and can be used as guidelines for estimating the Δ*G*° of long dangles; see Supplementary Tables 16–27 for summarized results. Figure 5e shows that other homopolymer dangles also exhibit a general asymptotically destabilizing effect as dangle length increases.

For comparison, Fig. 5f shows the Δ*G*° values of multinucleotide dangles inferred through HRM. The error bars on HRM-derived Δ*G*° values are 6- to 12-fold larger than those inferred based on native catalysis; simultaneously, the two different methods produce Δ*G*° values that are not inconsistent with each other (within 2 s.d.'s). Thus, native catalysis-based characterization represents a more precise method for measuring nucleic acid motif thermodynamics.

## Discussion

In this manuscript, we applied a well-characterized dynamic DNA nanotechnology circuit, the non-covalent catalyst, to the problem of DNA motif thermodynamics characterization. One particular qualitative difference in our motif thermodynamics characterization method is the fact that Δ*G*° values of individual motifs are measured isothermally in native temperature and buffer conditions. This is especially desirable for DNA biotechnology and nanotechnology applications because DNA thermodynamics in buffers needed for enzymatic amplification and self-assembly are often extrapolated from experiments at other conditions.

Using our method, we characterized the Δ*G*° contributions of (1) the ROX and Alexa532 fluorophores next to duplexes, (2) single-nucleotide dangles, and (3) multinucleotide dangles. Our measured parameters possessed high precision due to multiple-independent repeats of each experiment; we typically achieved a mean s.d. of <0.1 kcal mol^−1^. For all dangle parameters, we used ROX- and Alexa532-labelled strands to independently produce two sets of mean Δ*G*° values for internal consistency verification.

Our measurements of fluorophore thermodynamics may possess systematic error of up to 0.2 kcal mol^−1^ due to the presence of intermediate CZ, but our measurements of dangles cancels out this bias through subtracting two Δ*G*° values, so dangle Δ*G*° values should have no systematic bias. There are four sources of unbiased errors in our experiments: pipetting errors, temperature errors, gel band quantitation errors and synthesis errors. All but the last should be independent from experiment to experiment, so repeat experiments will tend to reduce the errors in the mean values.

The single-nucleotide dangle parameters are the only parameters in our studies that have corresponding literature reported values, and there is significant disagreement between our consensus values and literature values^59^. Common sources of error between our approach and previous melt curve approaches include oligonucleotide synthesis impurities and concentration errors. Because DNA oligonucleotide synthesis have advanced significantly over the past 20 years, we believe that our results based on oligonucleotides synthesized by modern optimized techniques is more representative of true thermodynamics. To calibrate the effects of oligonucleotide synthesis errors, we performed the same experiments on a set of independently ordered strands; the Δ*G*° values measured using different synthesis preps agreed with each other to within 0.09 kcal mol^−1^, on average. In addition, we believe that literature reported extinction coefficients^67^ are inaccurate at the 10% level, so that previous studies relying on absorbance at 260 nm for quantitating DNA concentration likely have a larger concentration error than our stoichiometric gel characterization protocol. Finally, our measurements directly interrogate the motif of interest rather than rely on linear algebra decomposition, and we cross-confirmed our Δ*G*° across two independent sets of fluorophores. For all these reasons, we believe our values to be more accurate.

To the extent that there may be differences in the true values of the dangle Δ*G*°, such differences may reflect a limitation of nearest neighbour model itself. A more complex model considering distal base identities to holistically evaluate nucleic acid structure stability may be warranted, but would also require significantly more experiments on multiple core sequence instances of the DNA motifs.

From simple biophysics, we postulated that multinucleotide dangles should contribute an increasing destabilizing thermodynamic effect, towards an asymptotic limit. We confirmed this hypothesis experimentally, and discovered that the saturation length is roughly 8 nt, with Δ*G*° roughly 0.56 kcal mol^−1^ more positive than a single-nucleotide dangle. We suggest that dangle length should be part of the standard model for DNA hybridization and folding software, and that the table in Fig. 5d be used as an empirical formula for estimating multinucleotide dangle Δ*G*° values. However, we currently do not have a quantitative biophysical model that predicts our measured multinucleotide dangle thermodynamic parameters, and encourage interested parties to explore area of research, for example using molecular dynamics simulations.

Performing PAGE is more labour-intensive than running a melt curve, and this is a disadvantage of our overall workflow. However, our approach generated motif Δ*G*° parameters with s.d.'s 6- to 14-fold lower than those obtained from HRM experiments. Statistically, 36- to 196-fold more experimental repeats of HRM would be needed to generate parameters of the same precision. In addition, PAGE experiments could potentially be automated, and other readout mechanisms may be possible. Possible approaches for downstream readout include liquid chromatography and/or microfluidics approaches. Scaling the presented method will be vital in obtaining high accuracy Δ*G*° parameters for a variety of motifs in a variety of temperatures and buffer conditions. For example, many artificial nucleotides with desirable properties, such as 2′-O-methyl RNA^68^, locked nucleic acids^69^ and peptide nucleic acids^70^,^71^, lack characterization beyond rudimentary rule-of-thumb melting temperature effects from base stacks. Establishment of an accurate and comprehensive nucleic acid motif thermodynamics database will help usher a new paradigm of knowledge-driven design of nucleic acid biotechnology and nanotechnology.

## Methods

### Reagents

All DNA oligonucleotides (oligos) were synthesized by and purchased from Integrated DNA Technologies (IDT; Coralville, IA). All oligos were HPLC purified by IDT, and quality controlled by capillary electrophoresis and electrospray ionization mass spectrometry. All oligos were received in solution form at roughly 100 μM concentration in 10 mM Tris-EDTA (pH 8.0) after HPLC purification. Secondary stocks were prepared by diluting DNA samples in 10 mM TE buffer with 12.5 mM MgCl_2_ (pH 8.0) or PBS (10 mM, NaCl 154 mM pH 7.4). Oligo sequences are listed in Supplementary Tables 28–40. Single stranded 10-nt resolution DNA ladder was purchased from IDT. 10–330-bp double strand ladder was purchased from Thermo Scientific.

Chemicals and buffer used include: ammonium persulfate, magnesium chloride hexahydrate (≥98%), Tween 20 (viscous liquid), glycerol (≥99%), acrylamide and bis-acrylamide (19:1) ratio stock solution (m/v concentration of 40%), and TEMED (N,N,N',N'-tetramethylethylenediamine) (≥99%). All chemicals were purchased from Sigma Aldrich unless otherwise noted. SYBR gold gel stain was supplied as 10,000x concentrated in DMSO from Life technologies. SYTO82 orange fluorescent nucleic acid stain was supplied as 5 mM solution in DMSO from Life technologies.

All the experiments were carried out in PBS (phosphate buffer saline) buffer (diluted from purchased 10x PBS) or 10 mM TE 12.5 mM MgCl_2_ buffer which is prepared from 100x TE buffer (1.0 M Tris-HCl pH 8.0, 0.1 M EDTA) and solid magnesium chloride hexahydrate. All the reactions were performed in Peltier-temperature controlled thermocyclers. All samples were covered with aluminium foil during hybridization reaction to avoid potential photobleaching. The gel apparatus was connected to a water bath to control temperature of inner chamber. The accuracy of temperature was calibrated with a thermocouple. Gel running buffer was always pre-incubated to the desired temperature before each experiment.

### Hybridization and displacement reactions

Before analysis by gel electrophoresis, oligonucleotide molecules were allowed to undergo isothermal hybridization and/or strand displacement. All reactions occurred in 650 μl microcentrifuge tubes (Corning), in 50 μl total reaction volume; 1x concentration corresponds to 300 nM final concentration in all cases. Samples were incubated at the reported temperatures for either 20 h (1x PBS) or 3 h (TE-MgCl_2_). A multi-thermocycler (Benchmark) was used for temperature control for reactions at 10 and 25 °C. A MasterCycler Personal (Eppendorf) with a heated lid (95 °C) was used for temperature control for reactions at 37 and 45 °C. All temperatures were verified to be accurate within 0.2 °C using a thermocouple sensor.

After completion of the hybridization and just prior to loading into gel, 15 μl of 30% glycerol (volume to volume) were added to each 50 μl sample to facilitate gel loading. We elected to use 30% glycerol rather than conventional gel loading buffer because the bromophenol blue or xylene cyanol FF chemicals appear to give significant signal in the ROX fluorescence channel; by using glycerol instead, we were able to minimize background signal.

### Polyacrylamide gel preparation

Polyacrylamide gels (10%) were used in all experiments; 50 ml of acrylamide solution were prepared by mixing 32.0 ml ultrapure water (Millipore) with 5.0 ml 10x TAE buffer (Amresco Inc) and 12.5 ml (40% 19:1 bis:acrylamide) stock solution. A measure of 300 μl of 10% ammonium persulfate (m/v) and 28 μl TEMED were subsequently added to the acrylamide solution. Roughly 10 ml of the final solution was transferred into each 1.5 mm thick XCell SureLock gel cassette (Invitrogen) using 5 ml transfer pipettes. Ten-well gel combs (Invitrogen) were inserted into the cassettes to seal the gel from air and form the desired number of wells. Gels were allowed to polymerize for ∼40 min before samples were loaded.

### Polyacrylamide gel electrophoresis

All PAGE experiments were performed using a Model 85–1010 gel box (Galileo Bioscience), with temperature regulated by an external water bath (Cleaver). For most experiments, 1x TAE was used as running buffer; 1x TAE and 12.5 mM MgCl_2_ was used to verify that reduced salinity in running buffer did not significantly change inferred Δ*G*° values. Running buffer was prepared by dilution from 50x TAE buffer and solid magnesium chloride hexahydrate. The gel running temperature was always set to be the same as the temperature of reaction. The voltage and duration each 8 cm gel was run at varied based on temperature: 140 V for 90 min for 10 °C experiments, 140 V for 60 min for 25 °C experiments, 85 V for 65 min for 37 °C experiments, or 75 V for 65 min for 45 °C experiments. The voltages were set to minimize joule heating. In all cases, gel running time was controlled by the power supply (Major Science). Gels were run in parallel in sets of two on the same power supply.

### Post-electrophoresis gel handling

At the end of electrophoresis experiments, the gel cassettes were cracked open using a gel cutter, and the gels were directly transferred to a Typhoon 9500 quantitative gel imager (GE Healthcare) for fluorescence imaging. For control experiments comparing observed bands to a DNA ladder, gels were further stained with 0.01% SYBR gold. The gel was transferred to staining solution (5 μl of SYBR gold into 50 ml of water), and stained for 30 min on a shaker. Then the gel was gently rinsed in deionized water for three times and transferred to scanner for imaging. Gels were scanned at 100 μm resolution with 450 V photomultiplier tuber voltage using appropriate laser and filter settings. Images were saved in both BMP and .gel format for subsequent software analysis.

### Gel band quantitation and analysis

Gel images were analysed using the version 8.1 Image Quant TL 1D gel analysis software (GE Healthcare). Gel bands were automatically detected by the software, fluorescence background was subtracted and fluorescence intensity of bands were reported by normalizing the single-stranded *F* band to 100 arbitrary units (Supplementary Note 1). By default, lanes were defined to be full-width centred on each visible lane, and a ‘rolling ball' background subtraction was used.

### HRM experiments

SYTO 82 was used as reporter dye for HRM experiments in either 1x PBS or Tris-MgCl_2_ buffer. 5p-Comp-T (300 nM) and the corresponding dangle strand (5′-A, 5′-T1A, 5′-T3A, 5′-T10A, 5′-T20A, 5′-A1A, 5′-A3A, 5′-A10A, or 5′-A20A) were mixed with 5 μM of SYTO 82, respectively. Each 10 μl sample was transferred to a quantitative PCR plate (Hard Shell PCR Plates 96-well thin-wall) for the melt experiment (CFX96 Real-Time System with C1000 Touch, Bio-Rad). The samples were heated to 95 °C, then cooled down to 30 °C, holding 10 s at each integer degree Celsius, with a ramp speed of 0.2 °C per sec. The melting process was measured by the fluorescence of SYTO 82. Melting temperatures determination and thermodynamic parameters calculation were described in Supplementary Note 11.

## Additional information

**How to cite this article:** Wang, C. *et al.* Native characterization of nucleic acid motif thermodynamics via non-covalent catalysis. *Nat. Commun.* 7:10319 doi: 10.1038/ncomms10319 (2016).

## Supplementary Material

Supplementary Information Supplementary Figures 1-92, Supplementary Tables 1-40 and Supplementary Notes 1-7

## Figures and Tables

**Table 1:**
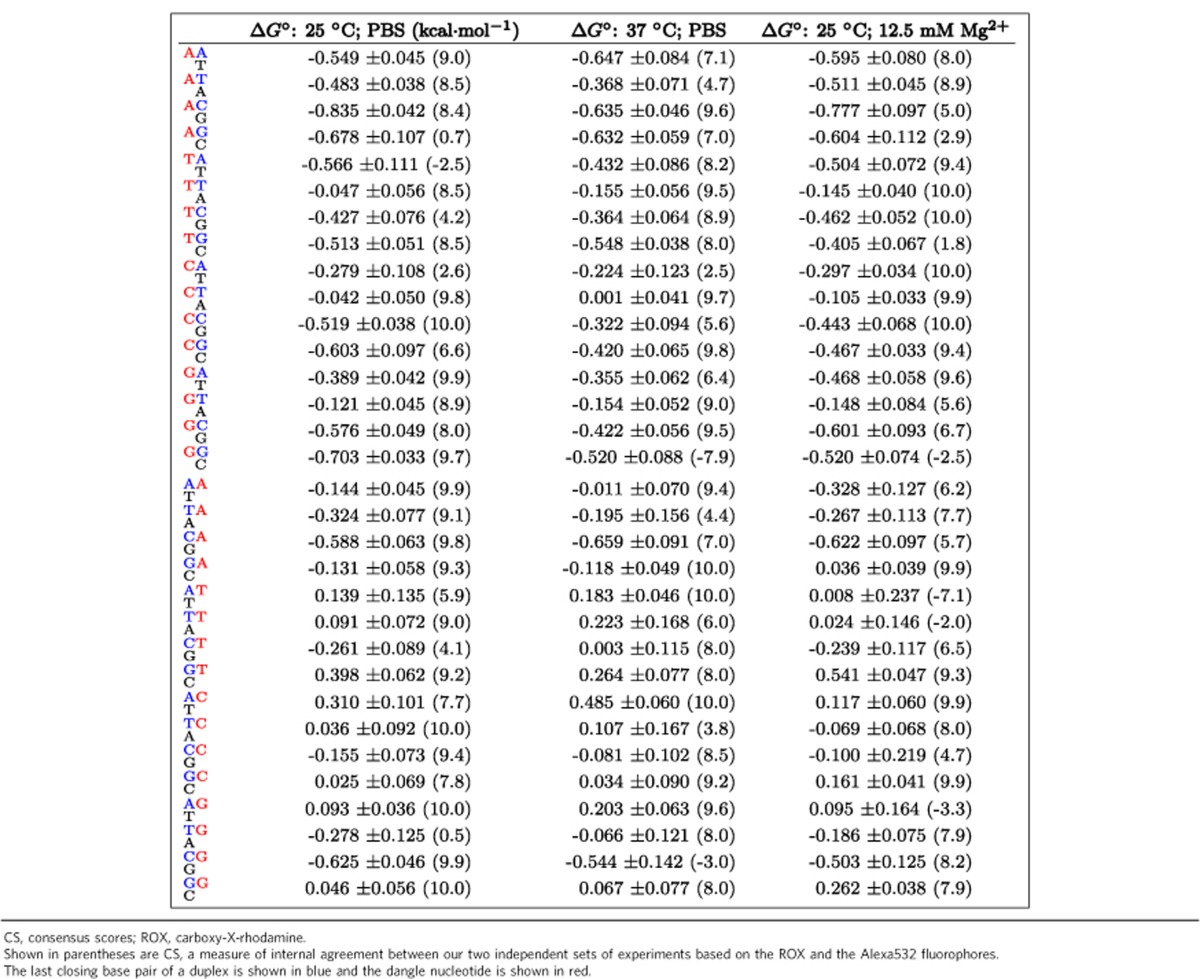
Consensus Δ*G*° of single-base dangles.
