# Supplementary material for: Native characterization of nucleic acid motif thermodynamics via non-covalent catalysis
Source: Nat Commun. 2016 Jan 19;7:10319. doi: 10.1038/ncomms10319 (PMC4735651; doi:10.1038/ncomms10319)
Supplement: Supplementary Information — Supplementary Figures 1-92, Supplementary Tables 1-40 and Supplementary Notes 1-7 [file ncomms10319-s1.pdf]

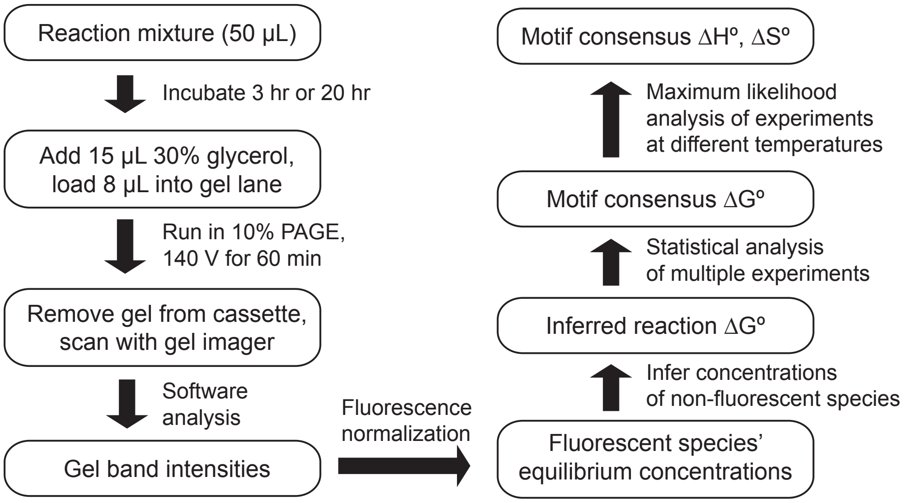

Supplementary Figure 1: Overall native characterization experiment and analysis workflow.

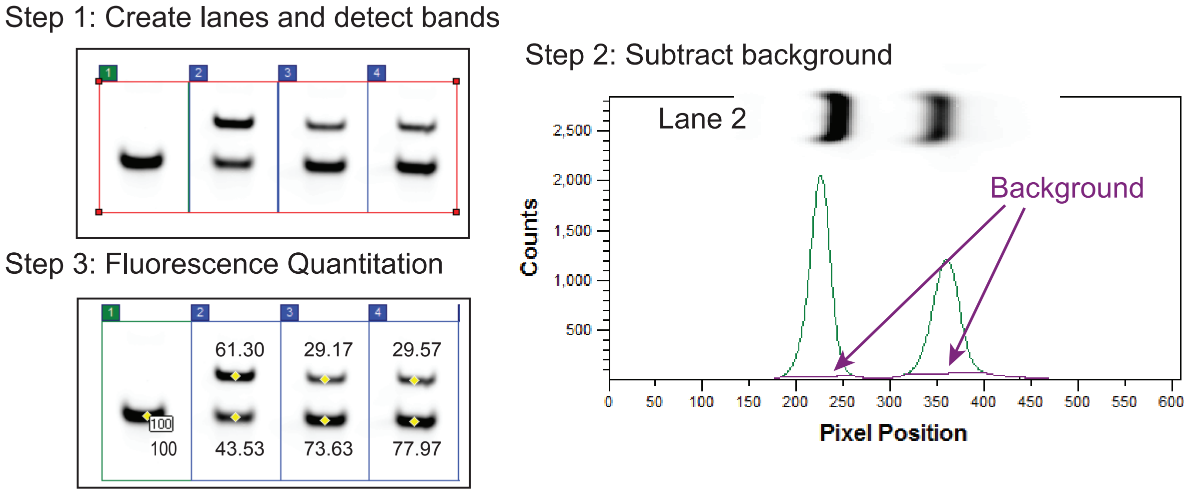

Supplementary Figure 2: Gel image analysis with Image Quant TL software. First, full-width lanes are created centered on the bands of interest. Next, bands are automatically detected, and fluorescence background of the gel is subtracted using a "rolling ball" algorithm packaged with the Image Quant TL software. Finally, the band intensities are calculated, normalized to the lane 1 band being 100 arbitrary units.

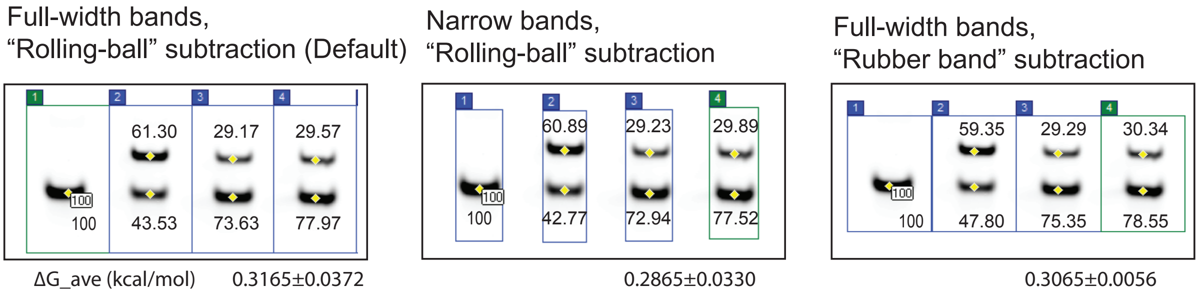

Supplementary Figure 3: Band intensities are robust to slight differences in gel image processing protocol. Narrowing the lane widths and omission of fluorescent background subtraction have only small impact on the relative intensities of the gel bands, and small impact on inferred ΔG°, because the increased or decreased fluorescence intensities apply roughly equally to all bands.

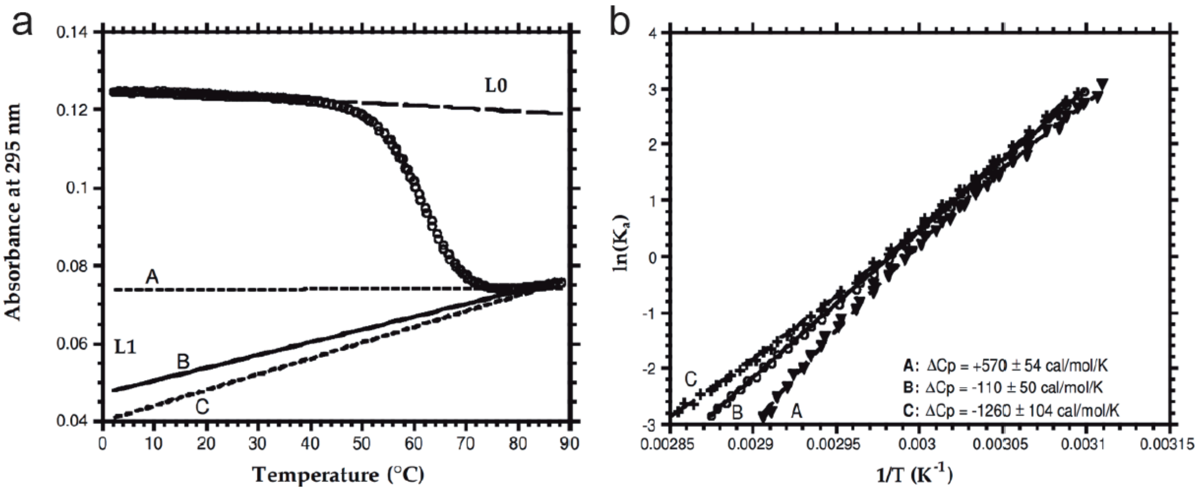

Supplementary Figure 4: Two figures were adopted from [1]. **(a)** Thermal analysis based on melting curve. Baselines chosen can be very subjective, which lead to bigger deviations of data. **(b)** Different ΔCp due to different baselines settings-the values turned out to be positive, negative or zero, leading to subjective error.

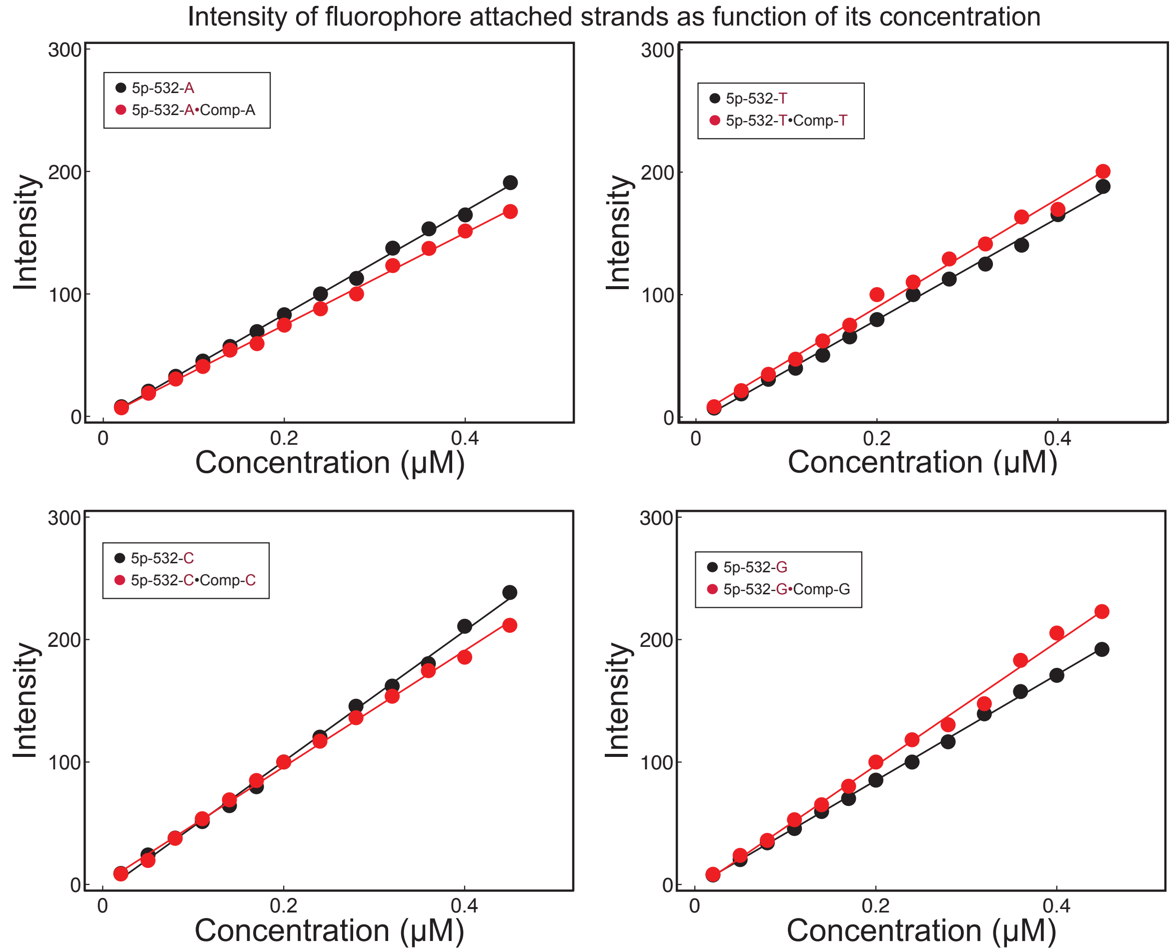

Supplementary Figure 5: Fluorescence intensity of each fluorophore modified strand as a function of concentration. Fluorescence intensity as a function of concentration of 5′-Alexa-532 modified oligonucleotides in single-stranded (black lines) or double-stranded (red lines) state. The nearest neighbor base is A (top left), T (top right), C (bottom left) or G (bottom right). Double-stranded DNA samples were prepared by incubating the relevant fluorophore-labeled oligonucleotide its complement for 30 min. Single-stranded DNA and dsDNA strands samples were prepared in a 50 μL volume and concentration as 20 nM, 50 nM, 80 nM, 110 nM, 140 nM, 170 nM, 200 nM, 240 nM, 280 nM, 320 nM, 360 nM, 400 nM and 450 nM, respectively.

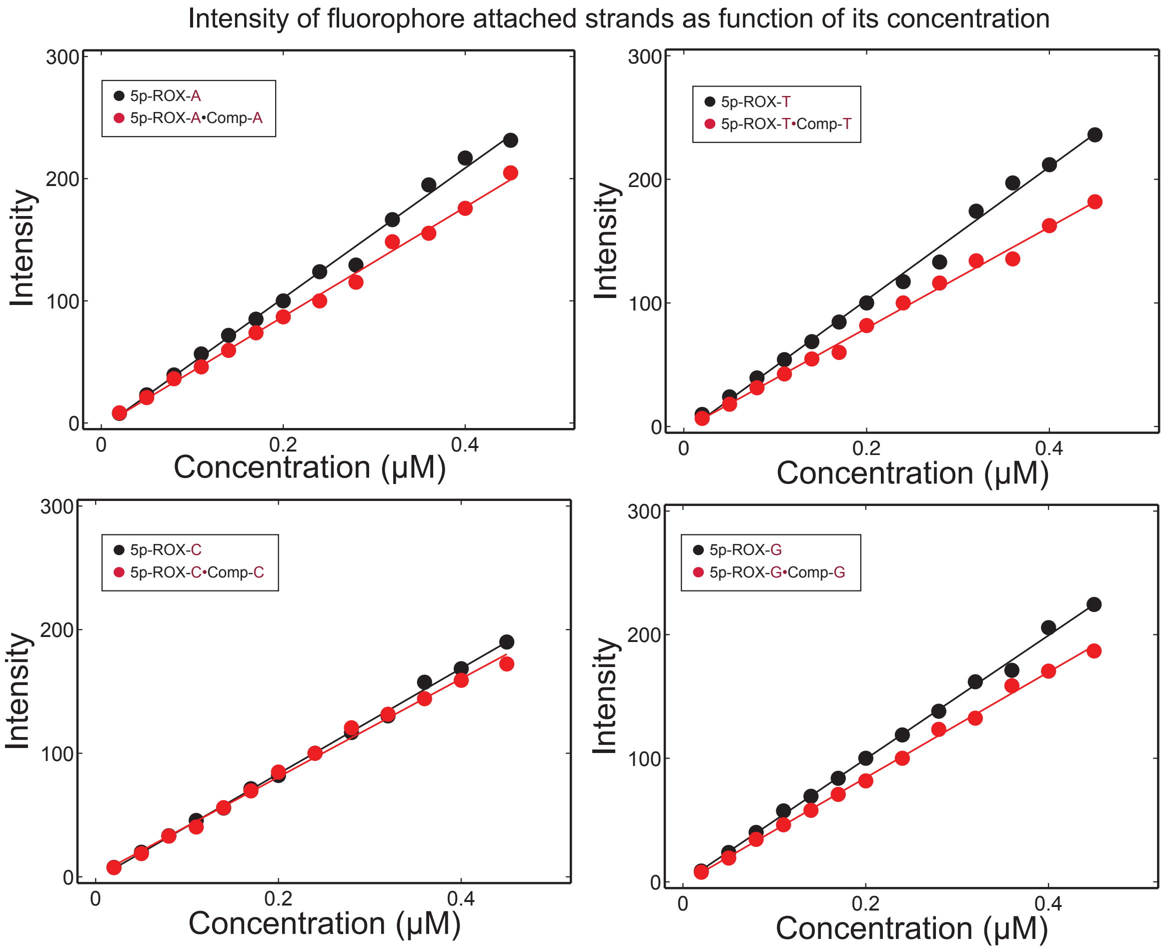

Supplementary Figure 6: Fluorescence intensity linearity for 5′-ROX oligonucleotides.

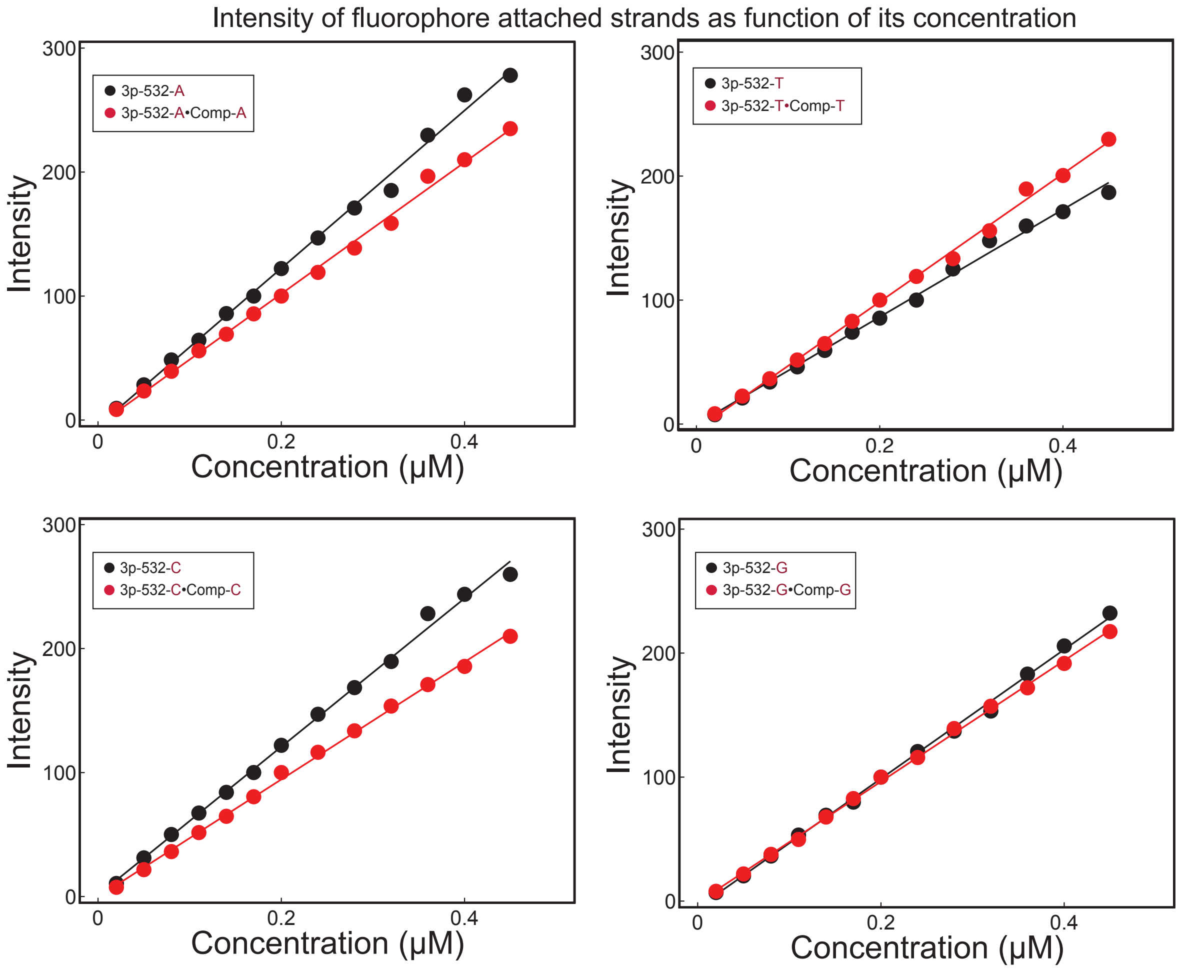

Supplementary Figure 7: Fluorescence intensity linearity for 3′-A532 oligonucleotides.

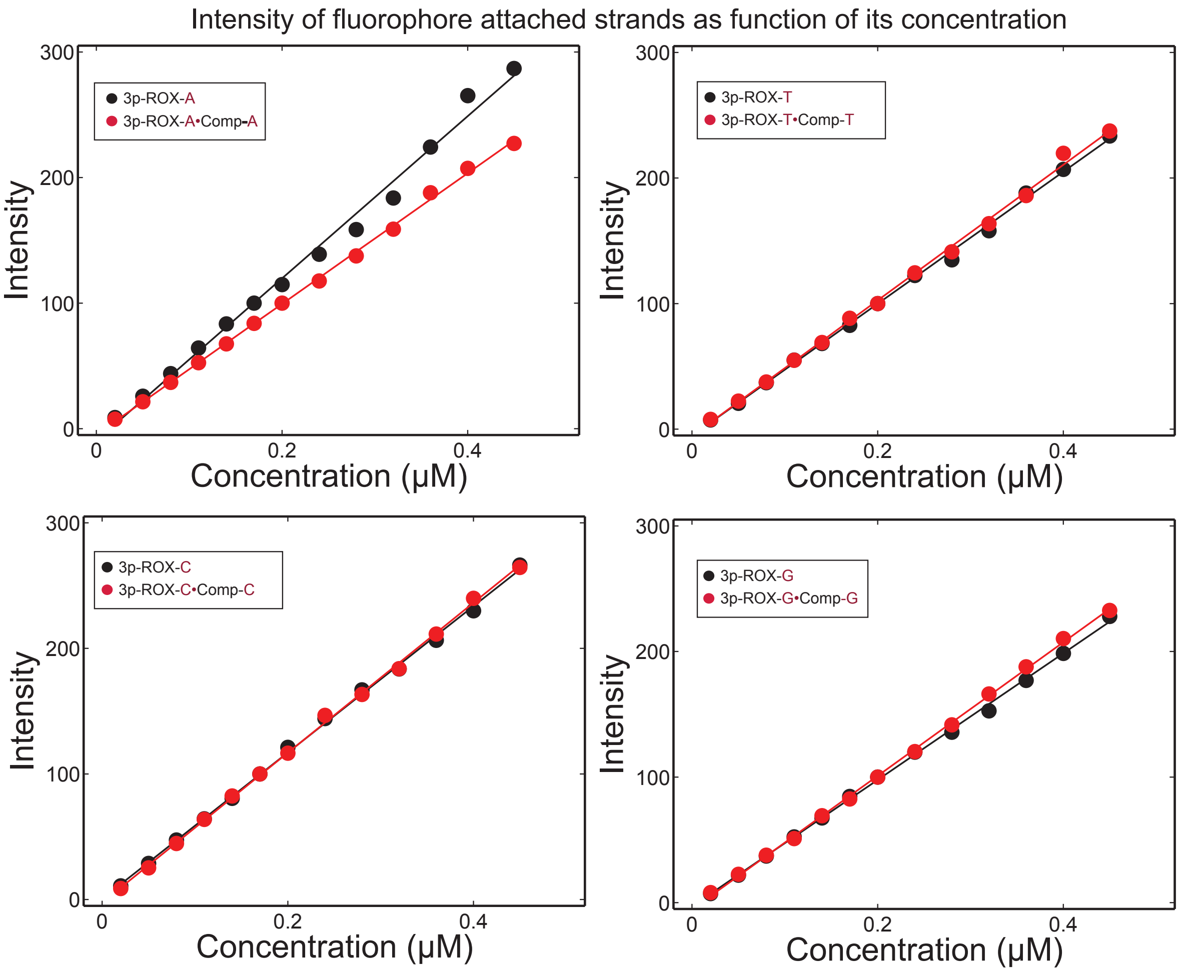

Supplementary Figure 8: Fluorescence intensity linearity for 3′-ROX oligonucleotides.

a

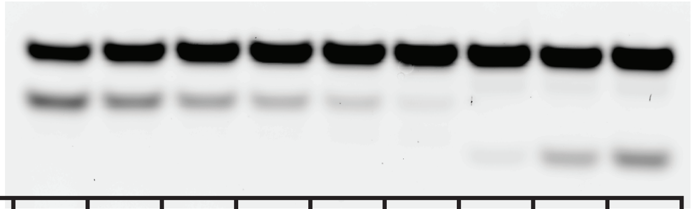

Nominal [3p-Ref-G]/[3p-Comp-G]	0.53	0.67	0.80	0.90	1.0	1.1	1.2	1.5	1.8
dsDNA Intensity	100	130.62	150.94	161.62	175.99	187.92	196.2	176.97	197.82
ssDNA Intensity	35.83	25.28	15.65	10.45	4.78	1.05	0.75	10.76	21.27

b

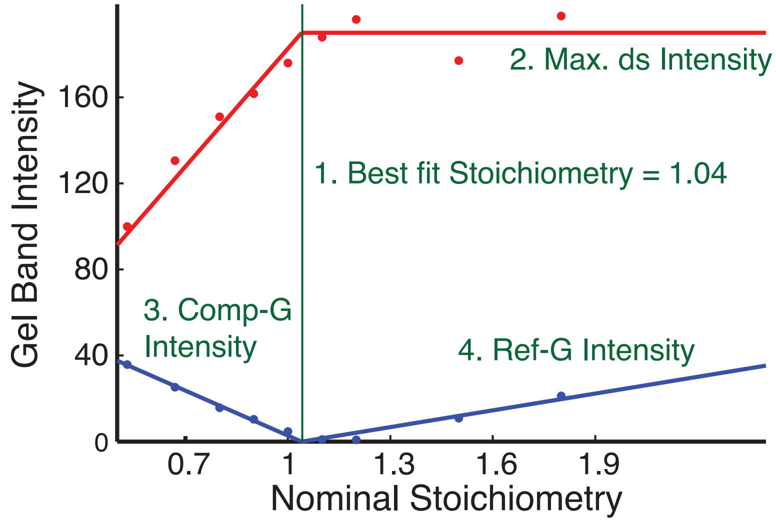

Supplementary Figure 9: Native stoichiometry PAGE, comparing 3p-Ref-G and 3p-Comp-G concentrations. **(a)** A native stoichiometry PAGE (SYBR Gold stained) to analyze the relative concentrations of 3p-Ref-G and 3p-Comp-G. Loading quantities are nominal, based on IDT's estimate of 100 μM for each oligonucleotide stock solution. As the concentration of 3p-Ref-G increases relative to that of 3p-Comp-G, the intensity of the double-stranded band increases, and the intensity of the single-stranded 3p-Comp-G band decreases. At perfect stoichiometry, there should be no single-stranded species. When the concentration of 3p-Ref-G is higher than that of 3p-Comp-G, the double-stranded band intensity should remain saturated, and excess single-stranded 3p-Ref-G results in a new single-stranded band whose intensity is proportional to the excess quantity. **(b)** Plot of double-stranded (red) and single-stranded (blue) DNA band intensities. Scattered cots represent experimental intensity data, and lines show best-fit predictions. Single-strand DNA normalized fluorescence intensity, duplex DNA normalized fluorescence intensity, and true stoichiometric ratio were fitted to the observed band intensities. For this particular pair of oligonucleotides, the stoichiometry ratio based on absorbance at 260 nm was 1.04. Similar PAGE analysis was performed for all unlabeled species. For fluorophore-labeled species, their stoichiometry to the unlabeled complementary strands are directly inferred from fluorescent PAGE analyzing the reaction ΔG° and will be discussed in that section.

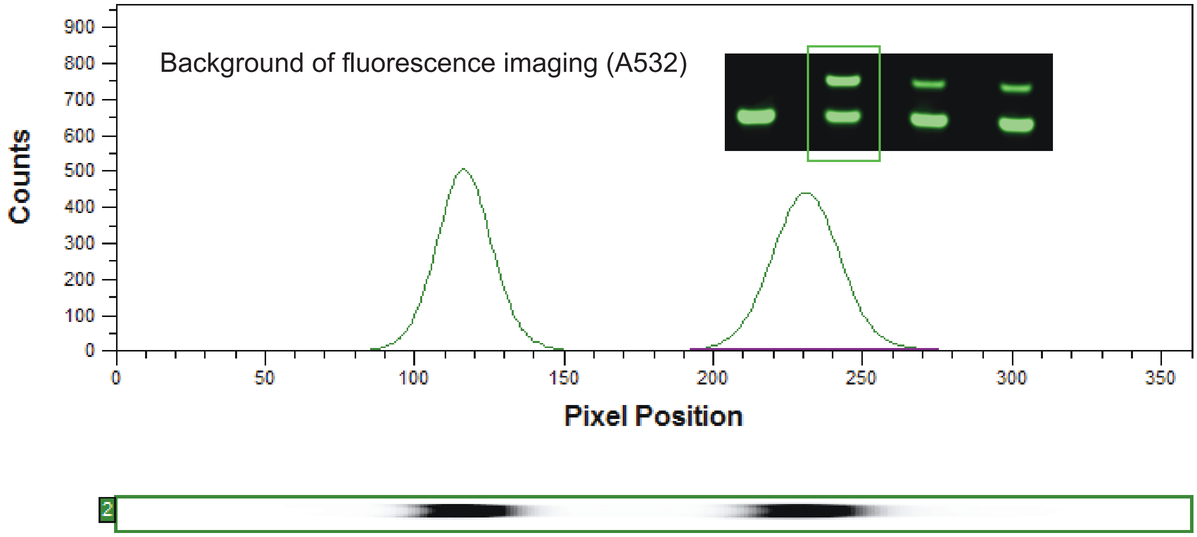

Supplementary Figure 10: Low background fluorescence in the Alexa-532 channel; the gel has virtually zero background signal.

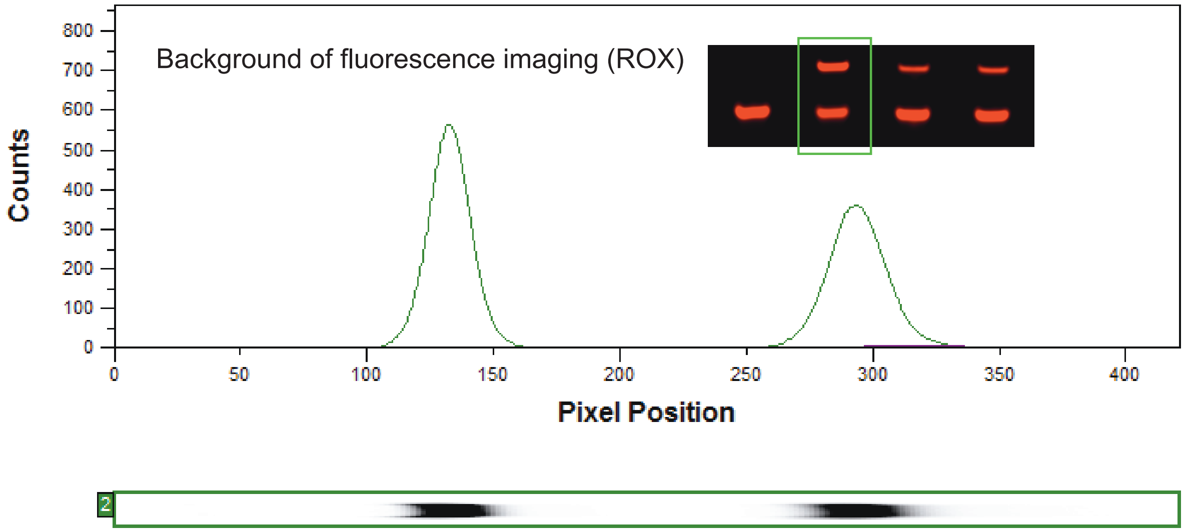

Supplementary Figure 11: Low background fluorescence in the Alexa-532 channel; the gel has virtually zero background signal.

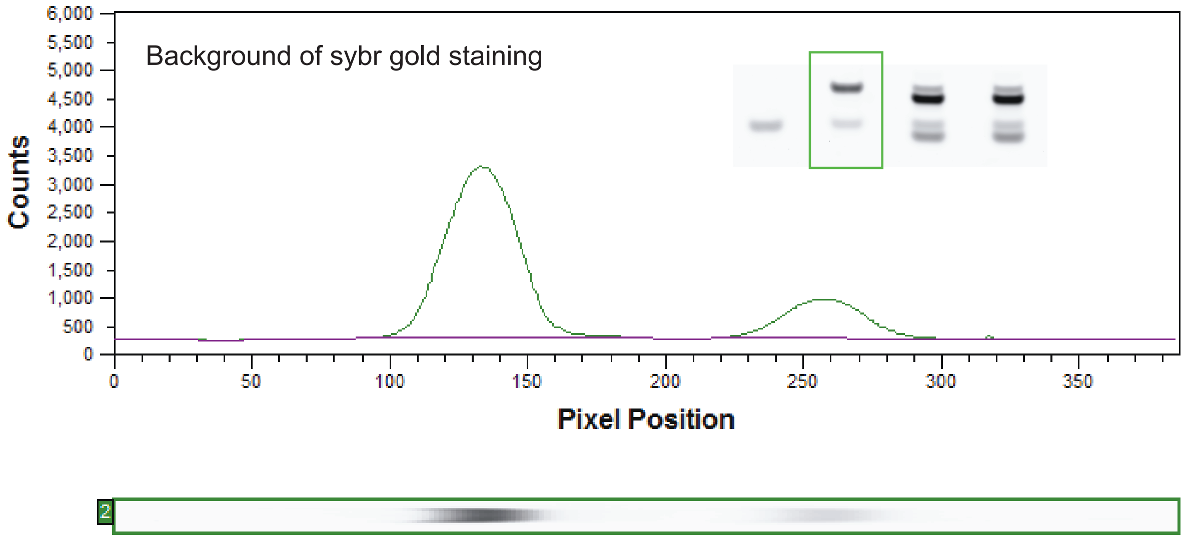

Supplementary Figure 12: Higher background of SybrGold stained gel. The image showed here is the exact same gel as in Fig.S5-2, but was subsequently stained in SybrGold and imaged in the SybrGold fluorescence channel.

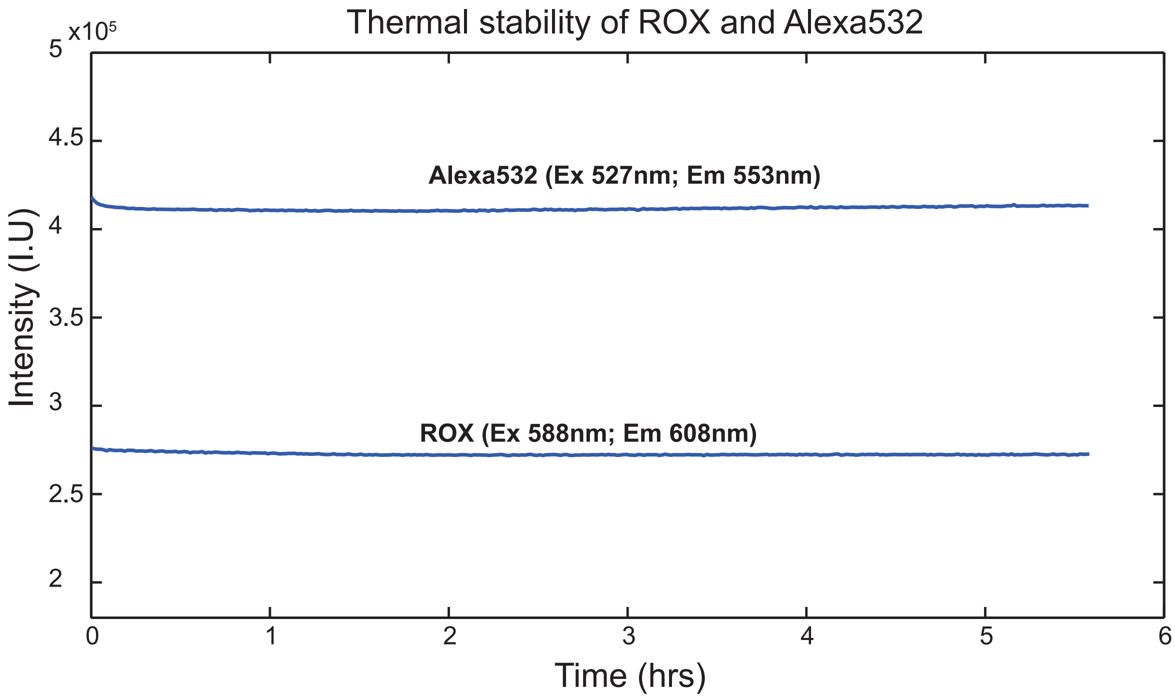

Supplementary Figure 13: Photostability of the ROX and Alexa-532 fluorophores. ROX- and A532-functionalized oligonucleotides were prepared in $MgCl_2$ buffer at 20 nM concentration in a volume of 1.2 mL. ROX-labeled oligo was excited at 588 nm and monitored at 608 nm; Alexa-532-labeled oligo was excited at 527 nm and monitored at 553 nm. Slit size for both experiments were 3 nm for both excitation and emission; the entire reaction proceeded at 45°C.

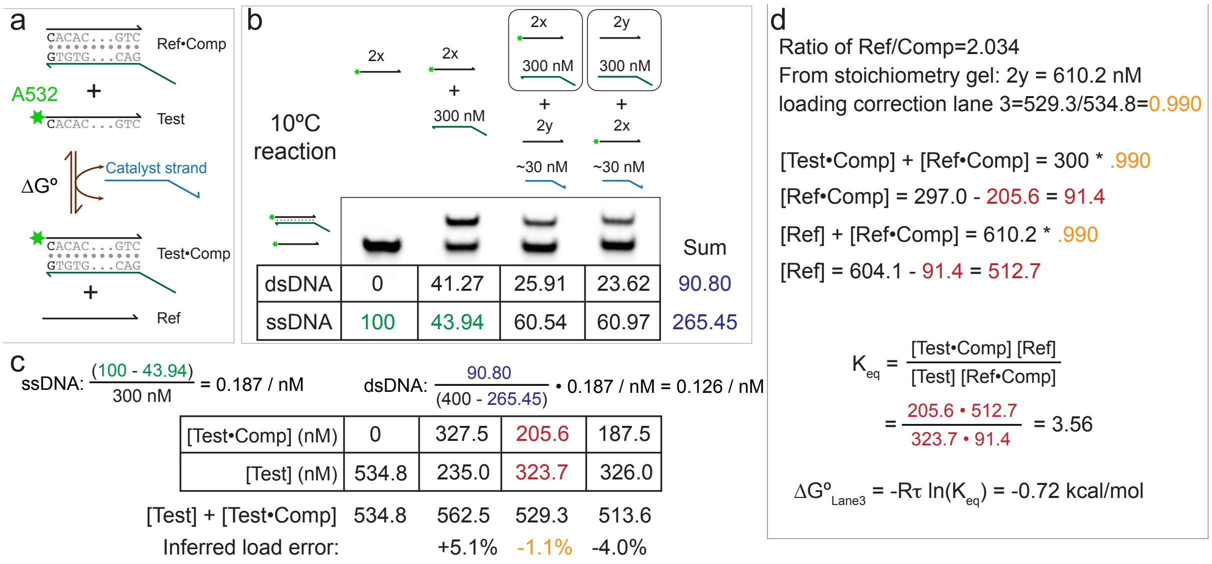

Supplementary Figure 14: Calculating ΔG° values from gel band intensities. **(a)** For this example, we characterized stabilizing thermodynamics of a 5′ Alexa-532 (A532) fluorophore next to a terminal cytosine nucleotide. **(b)** Representative gel image of A532 at 10°C, and quantitated band intensities. **(c)** The fluorescence efficiency of single-stranded species is calculated from the difference in intensity between lanes 1 and 2. The fluorescence efficiency of the double-stranded species is calculated from the sum of the intensities of double- and single-stranded species from all four lanes. The concentrations of the Test·Comp species and the Test species are then used to infer the per-lane loading error. Note that loading error for PAGE gels is typically somewhat larger than typical pipetting error due to capillary forces upon initial contact of the gel-loading tip with the running buffer. **(d)** The concentrations of all 4 relevant species are inferred from the concentrations of the two observed species, and K_{eq} is subsequently calculated.

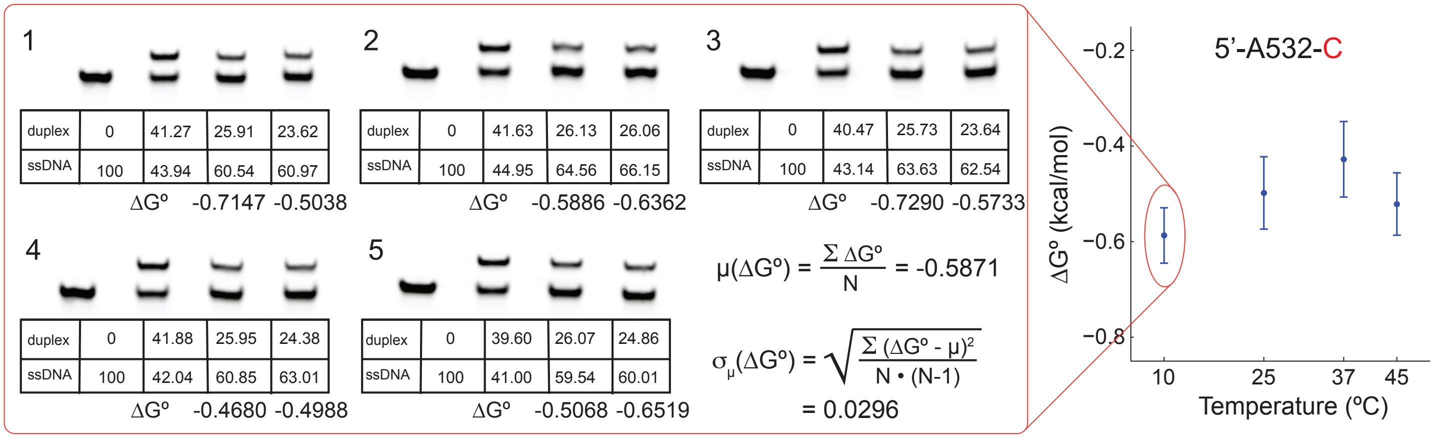

Supplementary Figure 15: The 10 different measurements of ΔG° values from 5 repeats of the same experiment produce a tight confidence interval for the true ΔG° value, based on simple statistics and assume Gaussian distributed experimental error. Similar experiments likewise produce μ and σ_μ values for ΔG° for the same motif at different temperatures.

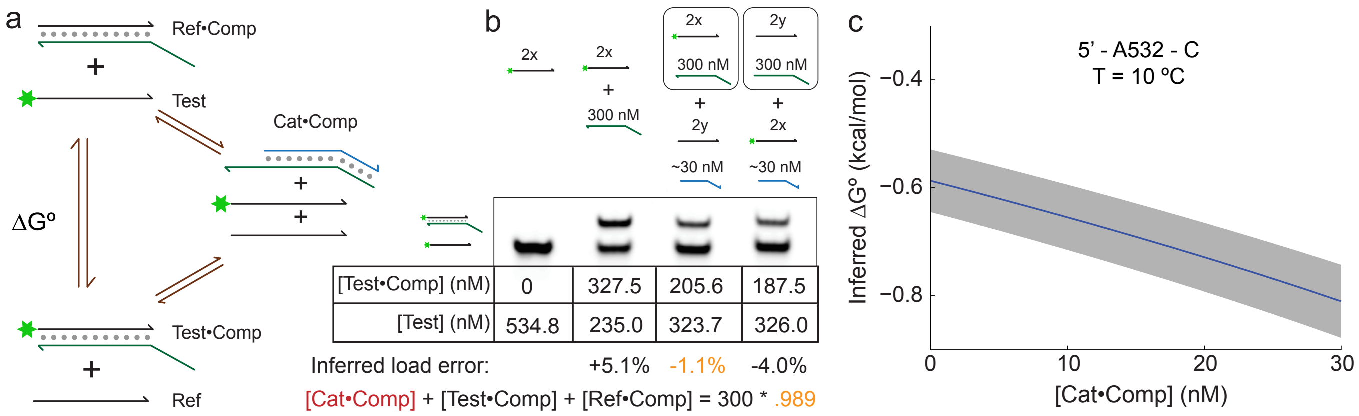

Supplementary Figure 16: Impact of ignoring the Cat·Comp intermediate species on estimated ΔG°. The experiments performed at 10°C is presented as an example for the affect of catalyst complex to the ΔG° calculation. (a) The Cat·Comp state has the fluorescent Test strand in single-stranded state, and thus overestimates the concentration of Ref·Comp. (b) Because we have no direct way of measuring the concentration of Cat·Comp, our current arithmetic that assumes [Cat·Comp] = 0 results in a positively-biased ΔG° value. (c) Inferred reaction ΔG° assuming different equilibrium values of [Cat·Comp]. Because the total concentration of catalyst is only 30 nM, the systematic bias in ΔG° estimate is no more than roughly 0.2 kcal/mol. Furthermore, because the catalyst sequence is identical for all experiments, it is very likely that the systematic bias is nearly identical for all experiments.

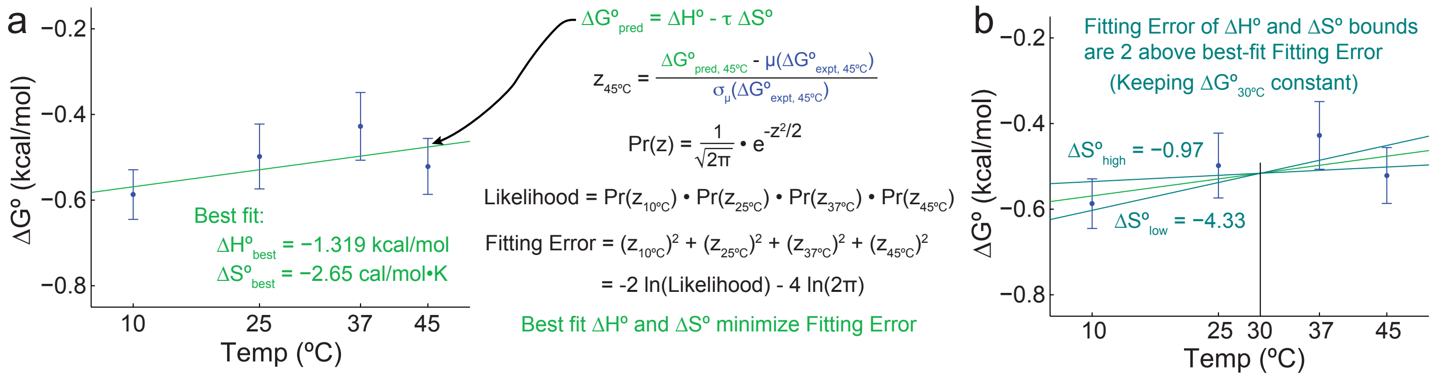

Supplementary Figure 17: Maximum likelihood fits of ΔH° and ΔS° values from ΔG° values. **(a)** The best-fit ΔH° and ΔS° are the pair of values that maximizes the collective likelihood (product of probabilities) for all 4 observed ΔG° values given their mean standard deviations, assuming Gaussian distributed error. The fitting error is calculated as the sum of the squares of the z-scores predicted by the ΔH° and ΔS° vs. observed, and is monotonic with the likelihood. **(b)** The confidence interval of ΔH° is simply calculated as the upper and lower bound values of ΔH° that produce a fitting error two times above the minimum (best) fitting error, holding ΔS° constant. The confidence interval of ΔS° unfortunately cannot be calculated the same way, as holding ΔH° would result in artificially small error bounds on ΔS°. Instead, we held the ΔG° at 30 °C constant, and determined the ΔS° confidence interval by co-varying ΔH° and ΔS°, once again setting the bounds at fitting error two times above the minimum.

5'-532-S1A Tris-Mg (TAE Mg running buffer)

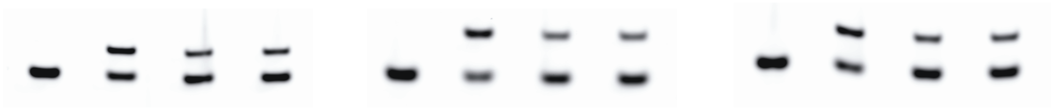

duplex	0	51.71	30.78	31.54
ssDNA	100	50.65	63.51	69.88

duplex	0	52.04	32.21	33.05
ssDNA	100	48.61	64.26	72.41

duplex	0	48.83	32.77	32.79
ssDNA	100	49.51	72.89	72.08

5'-532-S1CA Tris-Mg (TAE Mg running buffer)

5'-A532A ΔGº=-0.4240+/-0.0450 kcal/mol

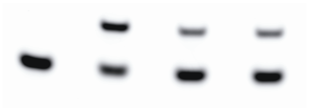

duplex	0	48.26	25.06	24.48
ssDNA	100	47.93	71.83	71.60

Alexa 532 5'-CA
T ΔGº=-0.3103+/-0.0467 kcal/mol

5'-ROX-S1A Tris-Mg (TAE Mg running buffer)

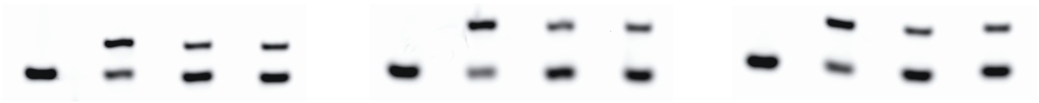

duplex	0	52.70	30.84	30.30
ssDNA	100	39.50	65.71	65.39

duplex	0	61.64	36.51	35.09
ssDNA	100	42.46	70.90	71.34

duplex	0	55.31	31.74	31.54
ssDNA	100	43.45	69.99	70.84

5'-ROX-S1CA Tris-Mg (TAE Mg running buffer)

5'-ROXA ΔGº=-0.3112+/-0.0390 kcal/mol

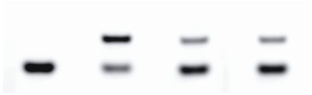

duplex	0	51.82	23.00	21.35
ssDNA	100	40.87	66.16	68.32

ROX 5'-CA
T ΔGº=-0.1558+/-0.0731 kcal/mol

ΔGº (kcal/mol) Reaction buffer Tris-MgCl2 (25ºC)	Running buffer	5'-ROXA	5'532A	5'-CA T (532)	5'-CA T (ROX)
	1xTAE	-0.290+/-0.056	-0.417+/-0.029	-0.295+/-0.030	-0.300+/-0.060
	1xTAE 12.5 MgCl2	-0.311+/-0.039	-0.424+/-0.045	-0.310+/-0.047	-0.156+/-0.073

Supplementary Figure 18: Fluorescent gels for characterizing ΔG° of fluorophores next to base A and single base dangle C next to A in the reaction buffer of 10 mM Tris-Mg, with running buffer as 1xTAE, 12.5 mM $MgCl_2$ at T=25°C.

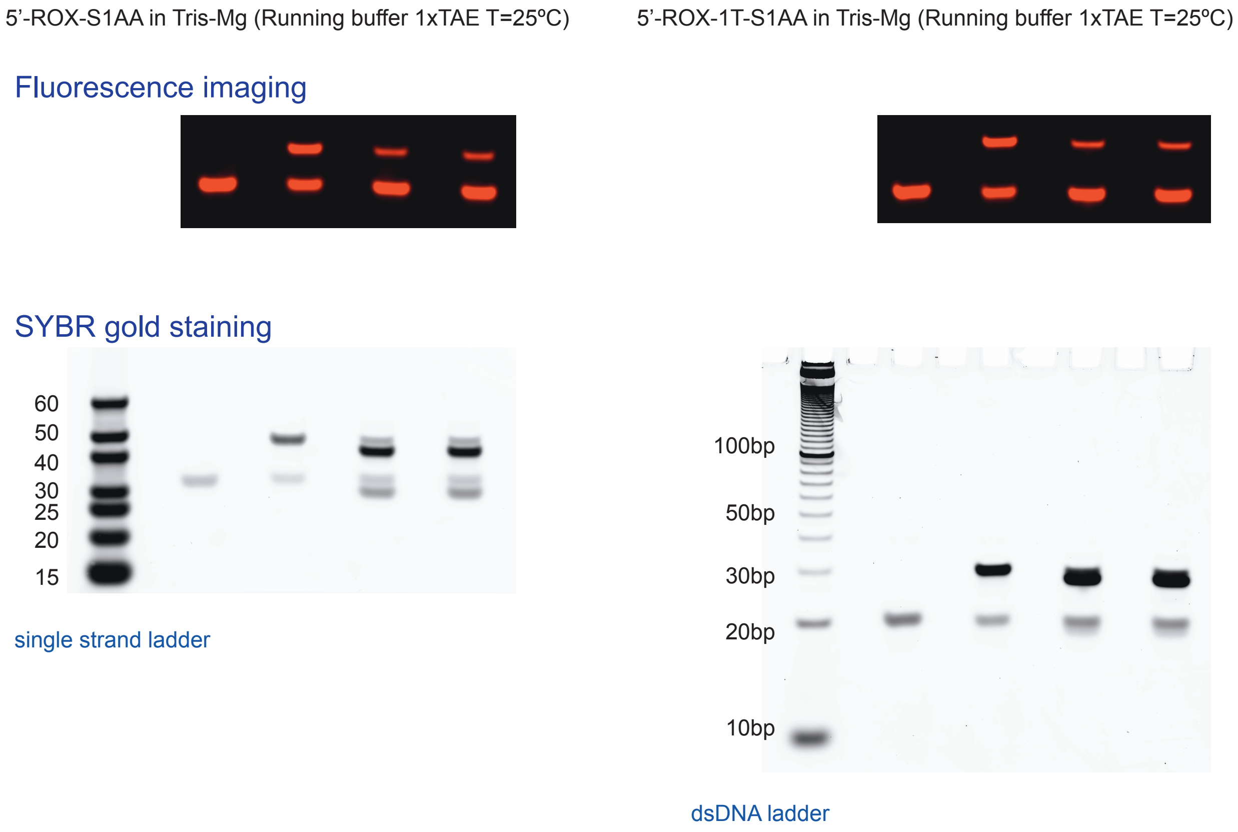

Supplementary Figure 19: Single-stranded DNA ladder 10-60 bases (10, 20, 30, 40, 50, 60) and double-stranded DNA ladder 10-330bp were used as reference in the SybrGold stained channel to confirm the identity of the gel bands.

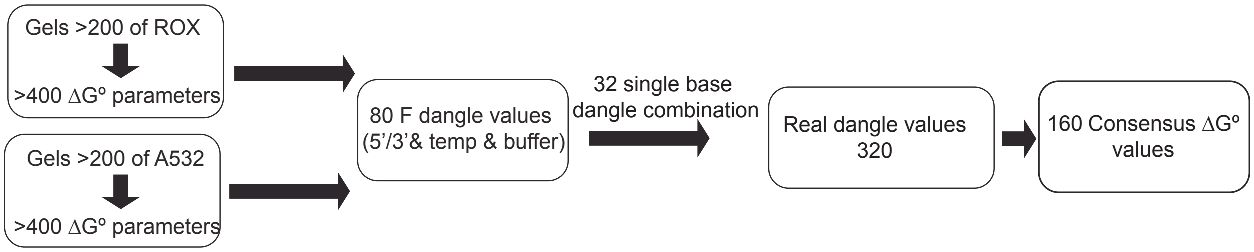

Supplementary Figure 20: Overall analysis workflow for single base dangle characterization.

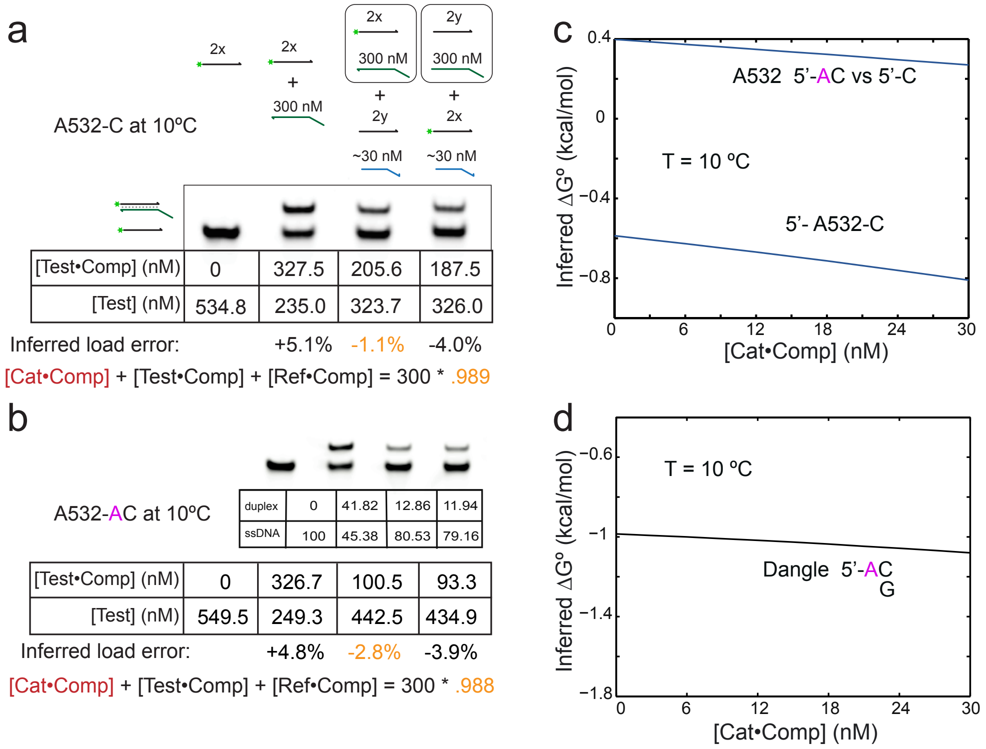

Supplementary Figure 21: Effect of catalyst strand on inferred ΔG° parameters. Our PAGE experiments were run at the same temperatures (10°C, 25°C, 37°C, 45°C) as the hybridization/catalysis reactions. An experiment run at 10°C as an example to show the effect of catalyst to the values of ΔG°. **(a)** An experiment run at 10°C as an example to show the effect of catalyst to the values of ΔG°; this gel assays the stabilizing effect of the Alexa-532 fluorophore when next to cytosine. **(b)** Gel image for 5′-AC dangle characterization. **(c)** Effects of intermediate CZ on the inferred ΔG° of fluorophore, and fluorophore with dangle. **(d)** Subtraction of the two values in panel (c) result in the real ΔG° value of a single-base dangle. The real ΔG° is not significantly affected by the concentration of CZ.

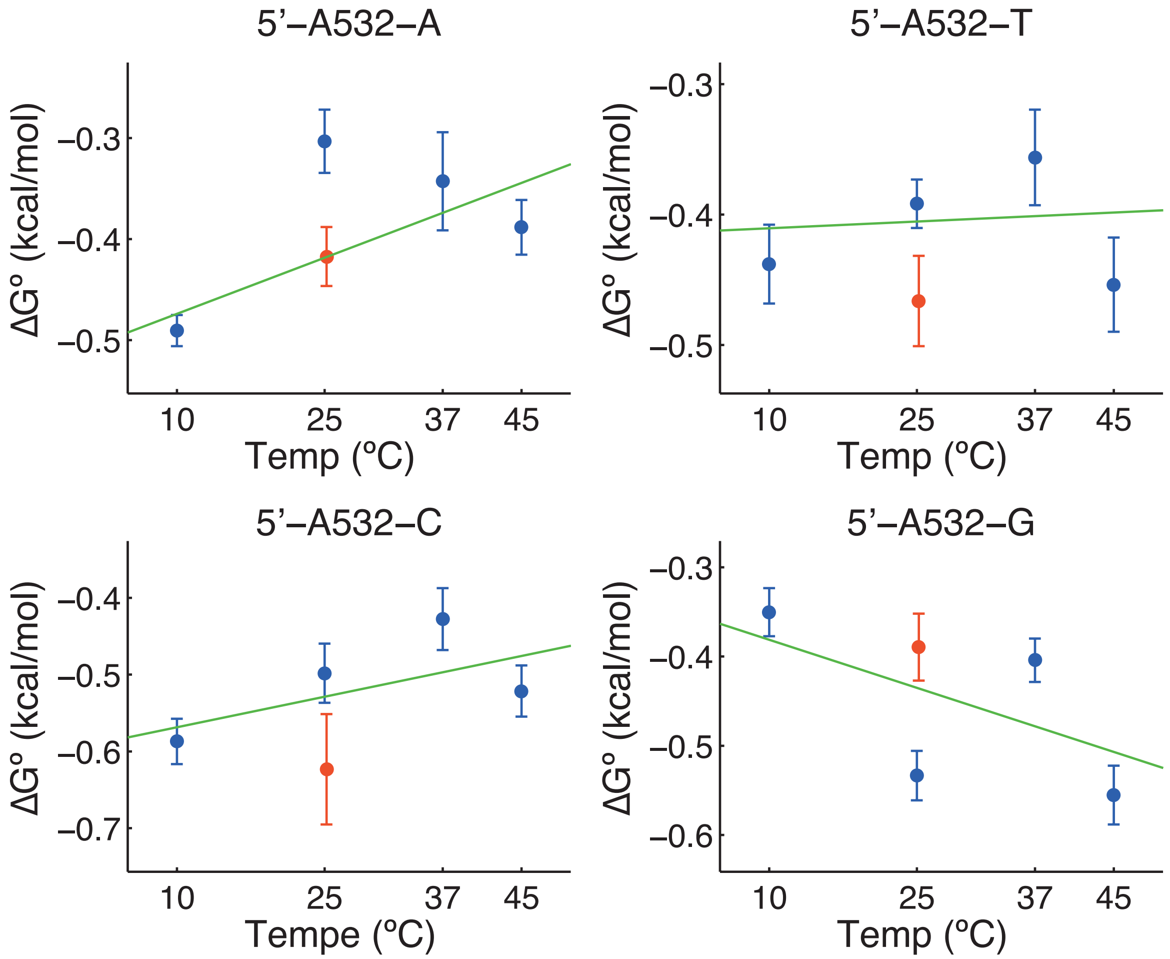

Supplementary Figure 22: $\Delta G°$ values of fluorophore Alexa 532 at four different temperatures (10°C, 25°C, 37°C and 45°C) in 1xPBS buffer and 25°C in Tris-$MgCl_2$ buffer near different bases were investigated. The graph on the top left shows $\Delta G°$ values next to base A; next to base T (top right); next to base C (bottom left); next to base G (bottom right).

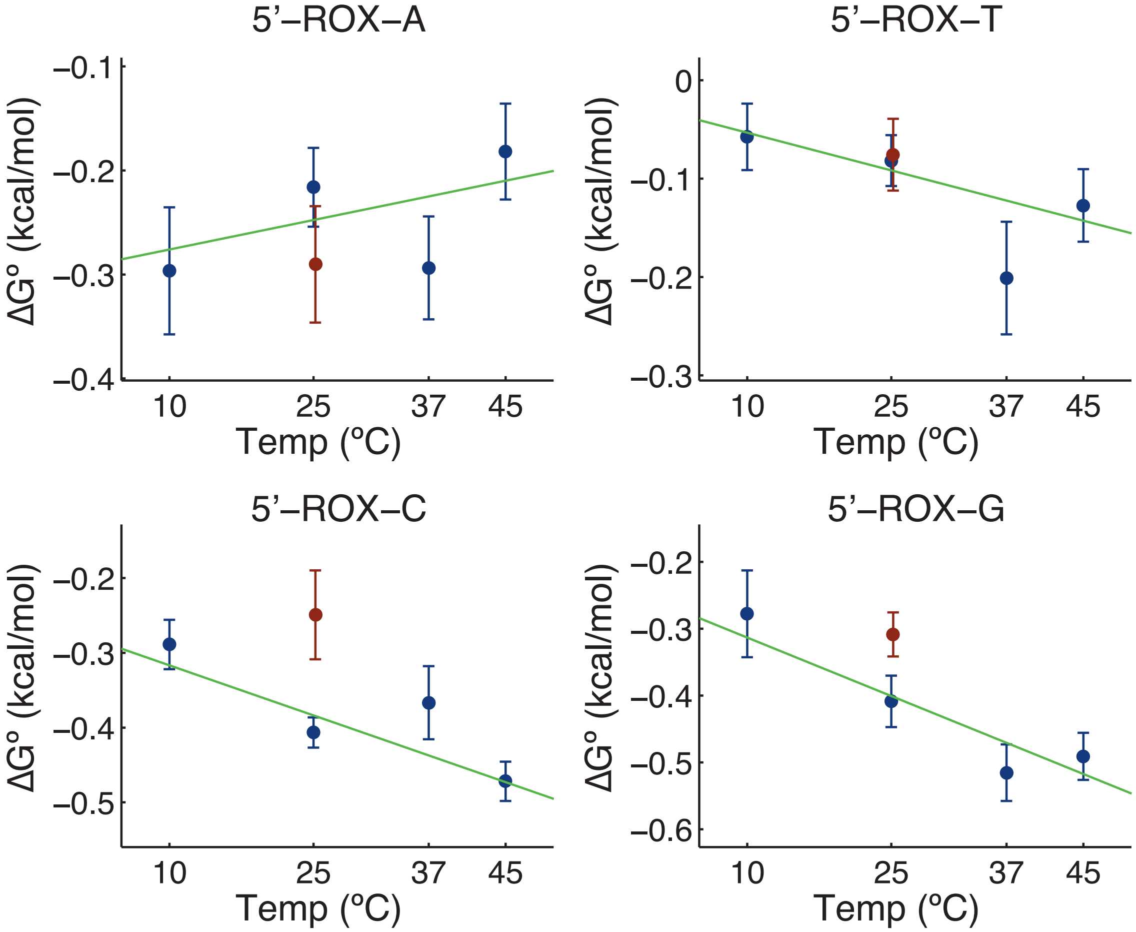

Supplementary Figure 23: ΔG° values of 5′ ROX fluorophores.

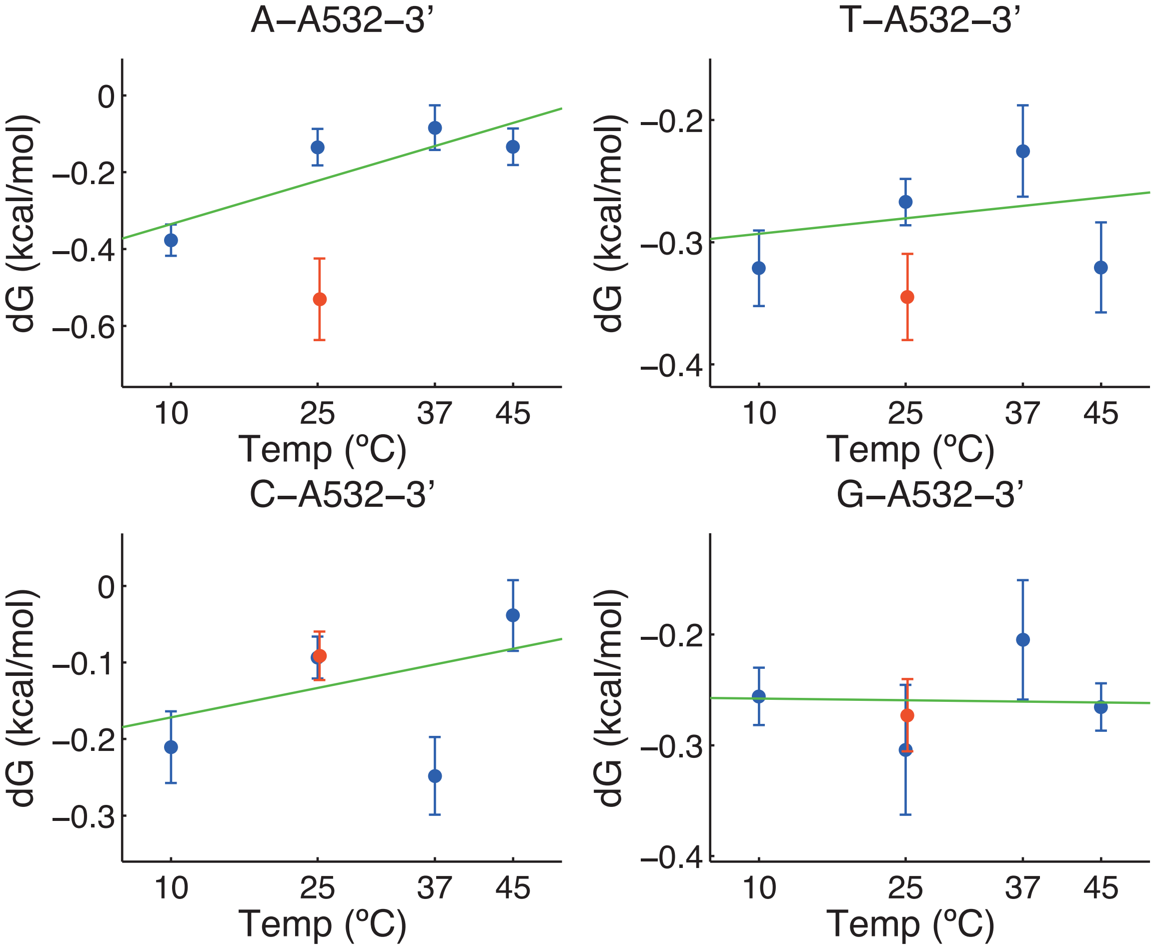

Supplementary Figure 24: ΔG° values of $3'$ Alexa-532 fluorophores.

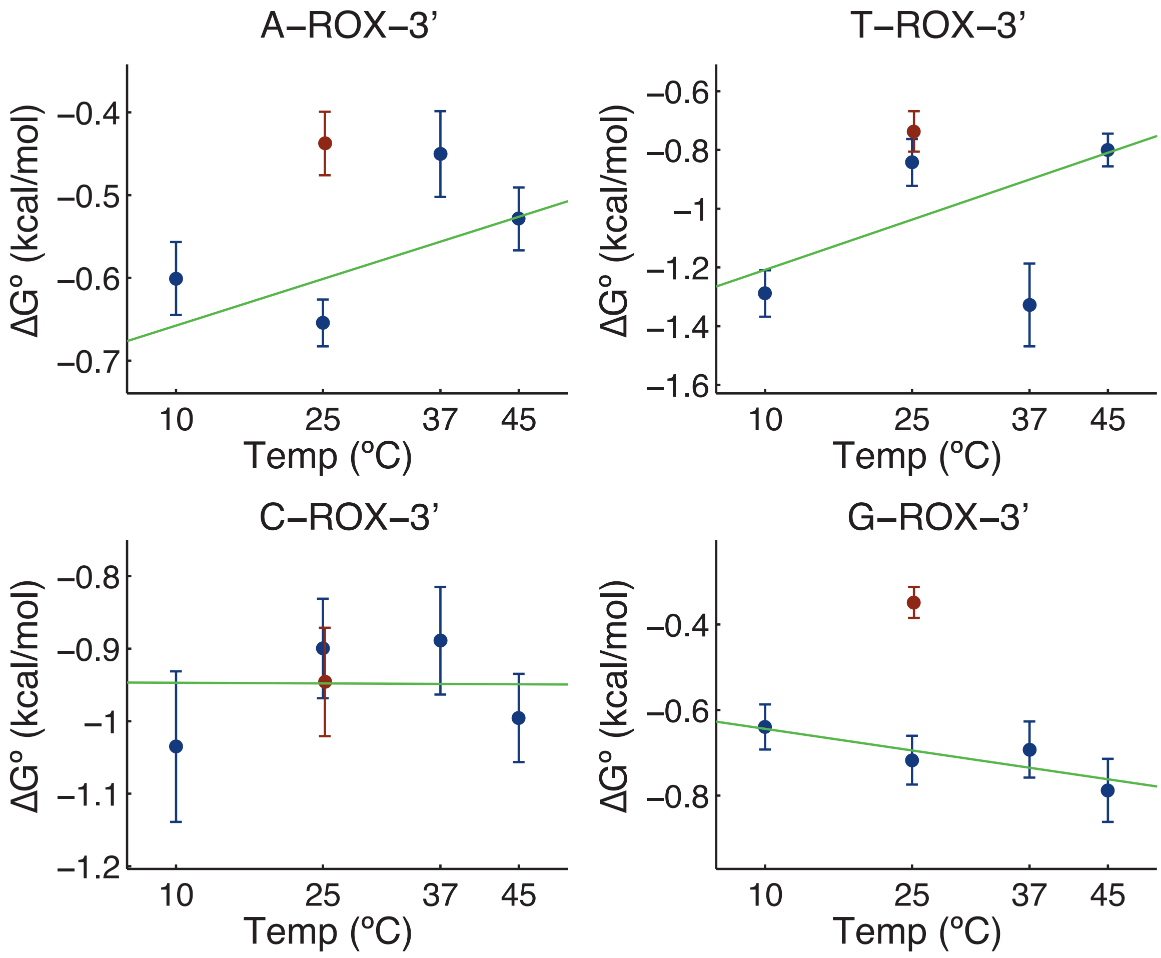

Supplementary Figure 25: ΔG° values of $3'$ ROX fluorophores.

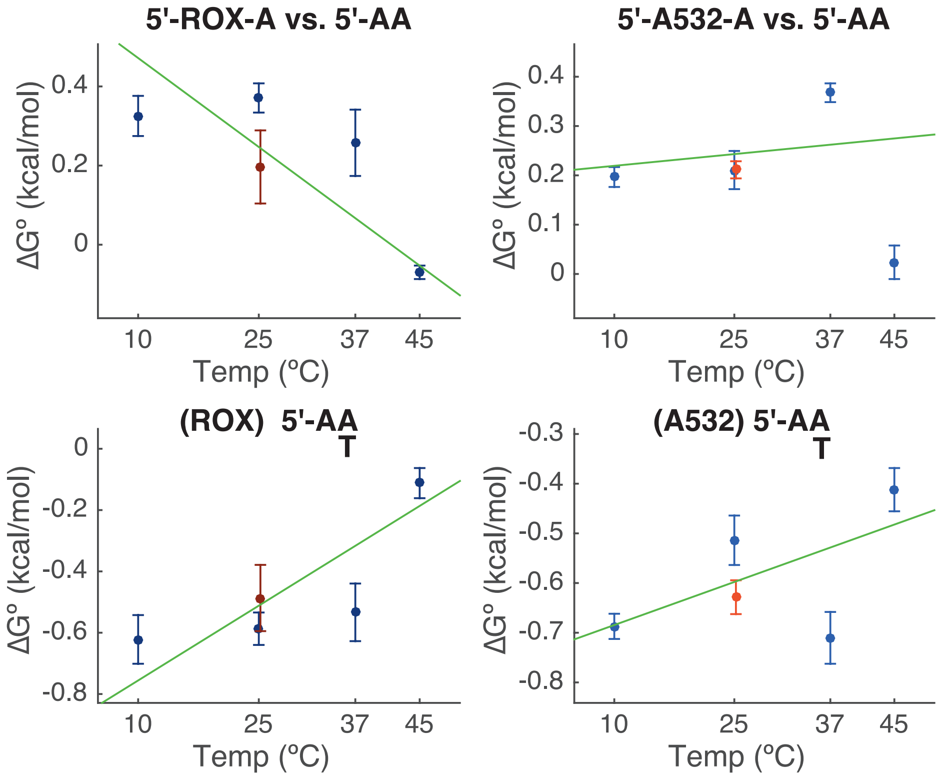

Supplementary Figure 26: Summary of best-fit-raw and real ΔG° values of dangle at four temperatures.

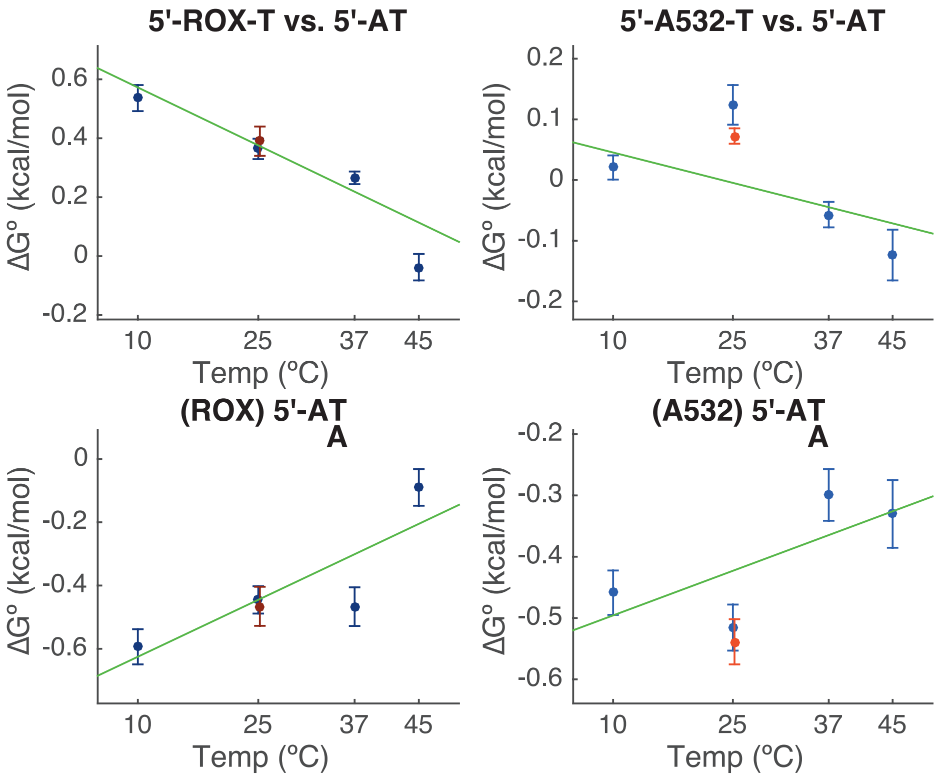

Supplementary Figure 27: Summary of best-fit-raw and real ΔG° values of dangle at four temperatures.

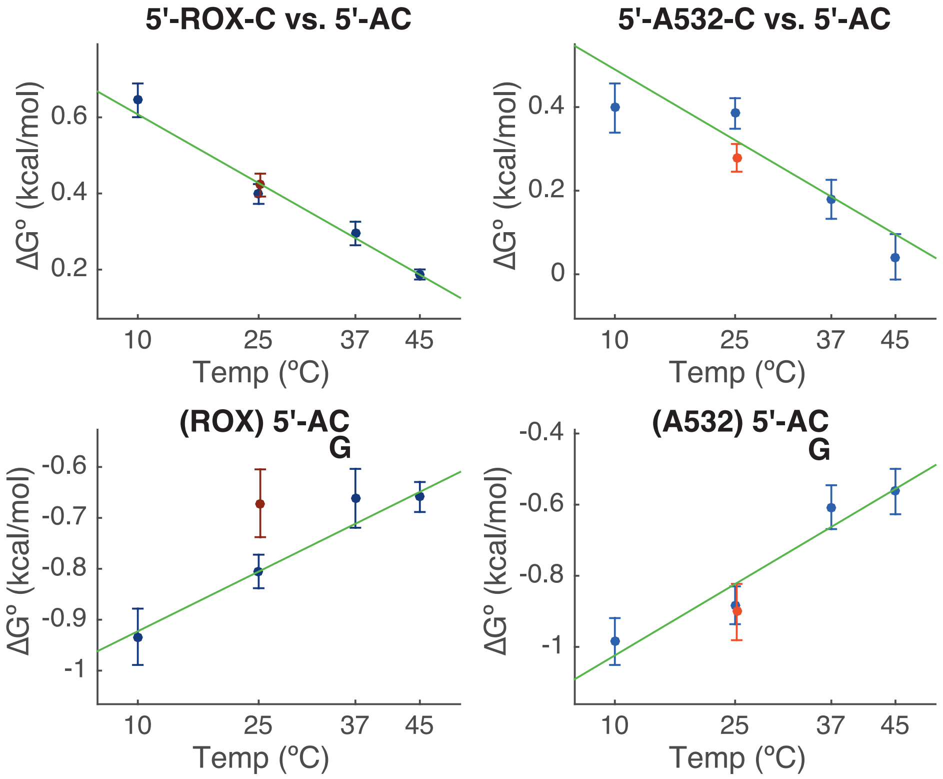

Supplementary Figure 28: Summary of best-fit-raw and real ΔG° values of dangle at four temperatures.

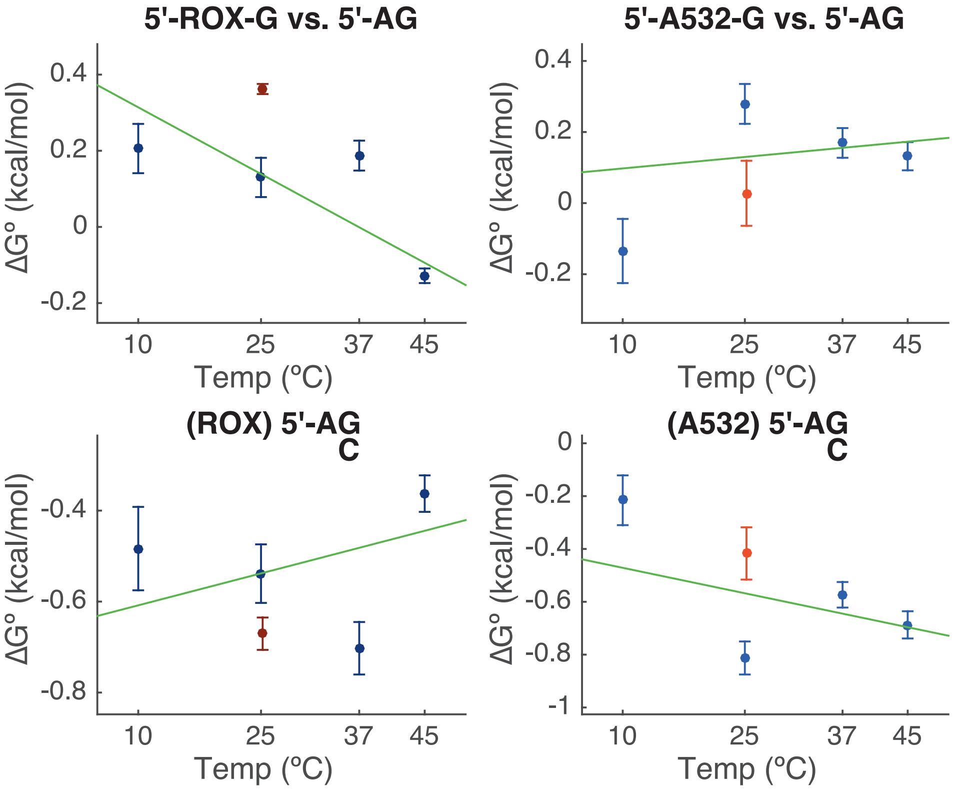

Supplementary Figure 29: Summary of best-fit-raw and real ΔG° values of dangle at four temperatures.

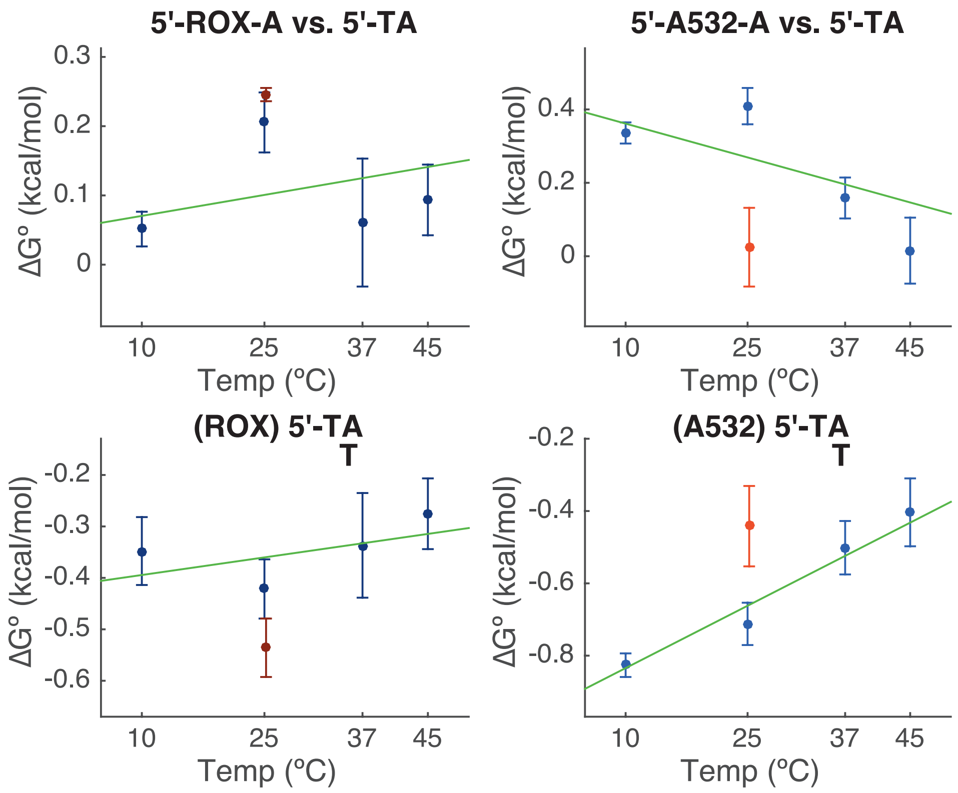

Supplementary Figure 30: Summary of best-fit-raw and real ΔG° values of dangle at four temperatures.

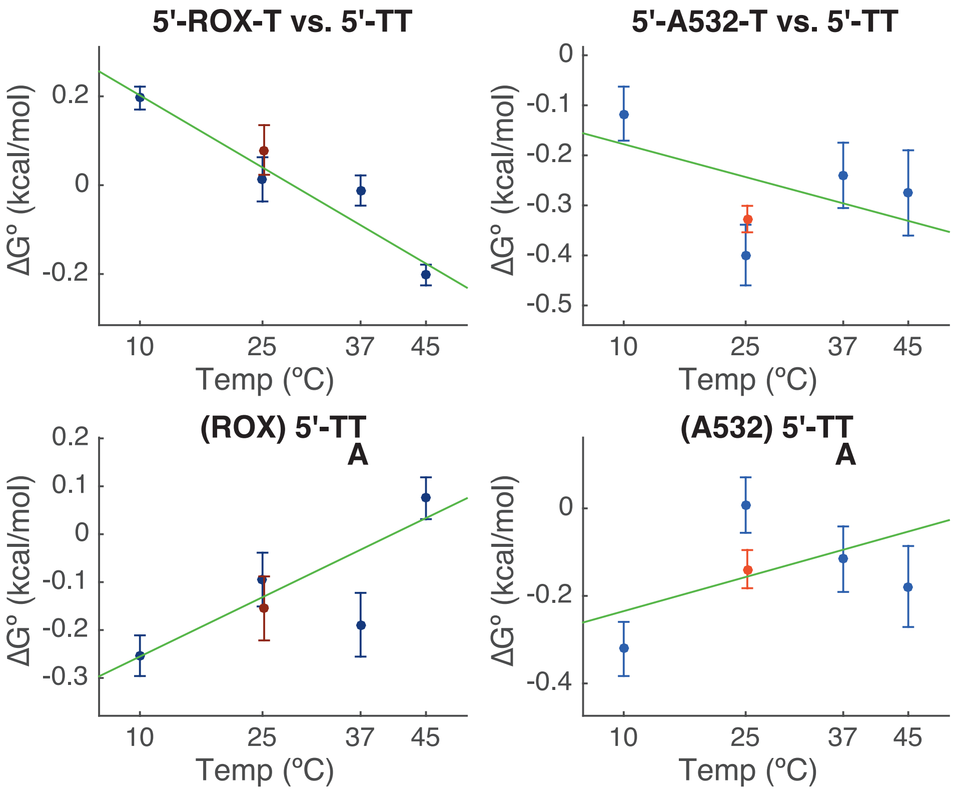

Supplementary Figure 31: Summary of best-fit-raw and real ΔG° values of dangle at four temperatures.

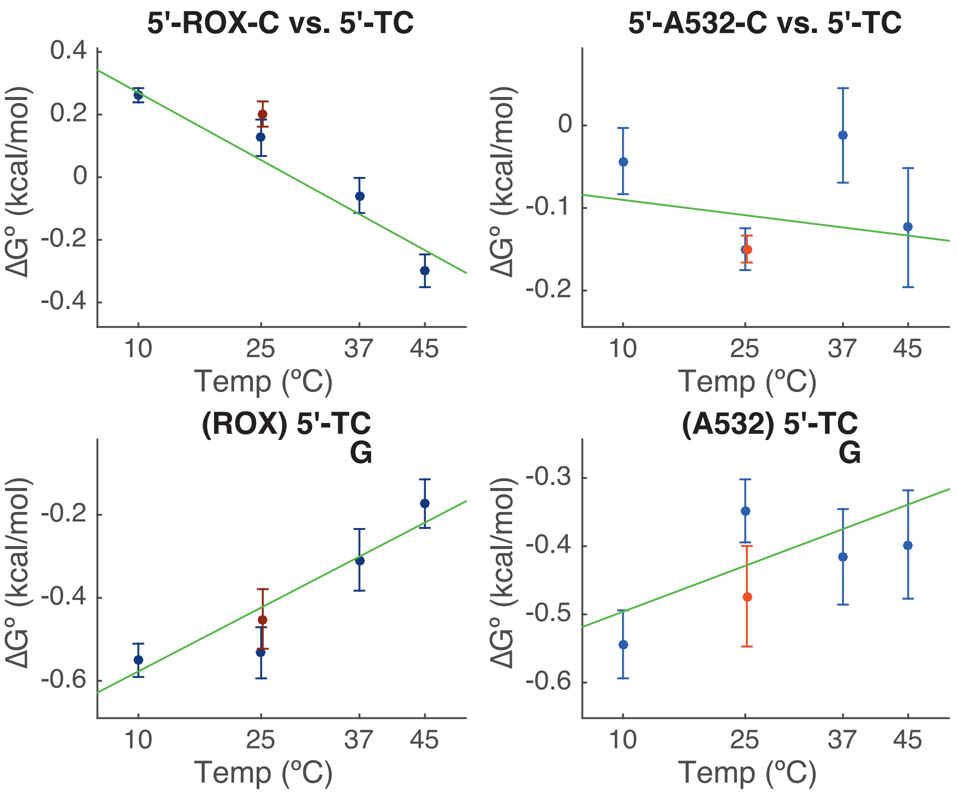

Supplementary Figure 32: Summary of best-fit-raw and real ΔG° values of dangle at four temperatures.

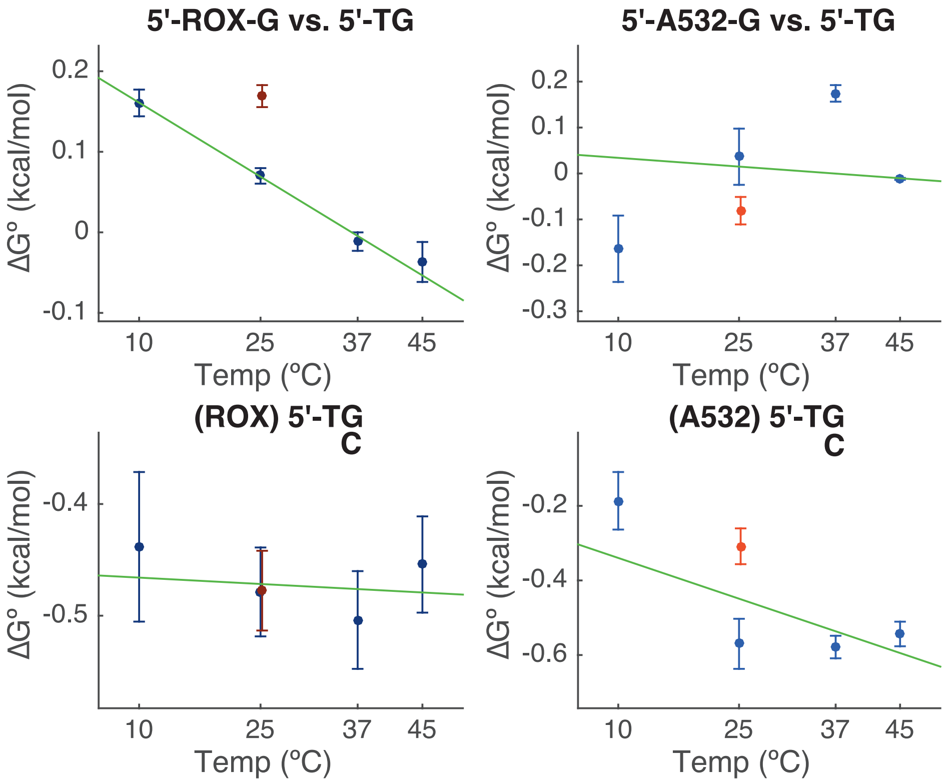

Supplementary Figure 33: Summary of best-fit-raw and real ΔG° values of dangle at four temperatures.

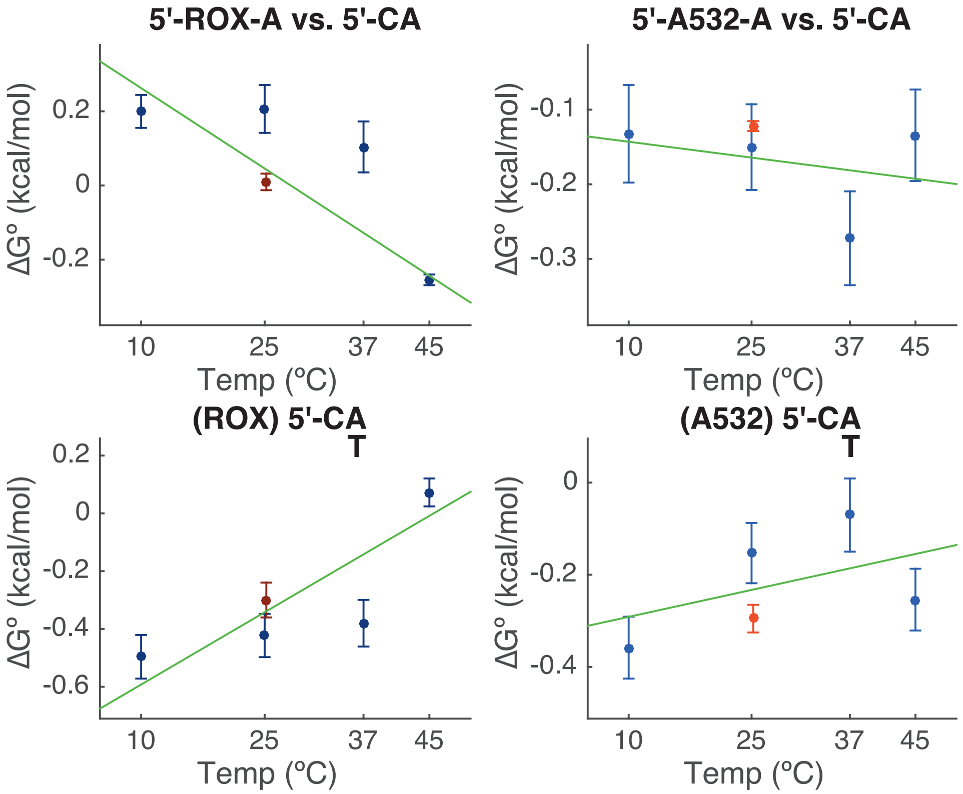

Supplementary Figure 34: Summary of best-fit-raw and real ΔG° values of dangle at four temperatures.

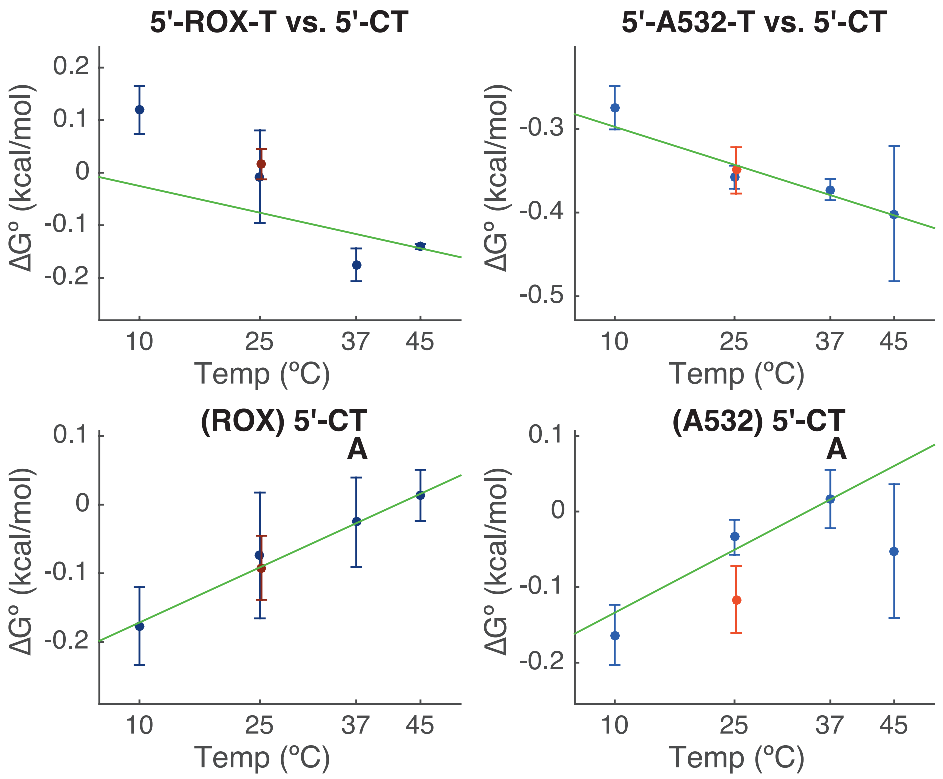

Supplementary Figure 35: Summary of best-fit-raw and real ΔG° values of dangle at four temperatures.

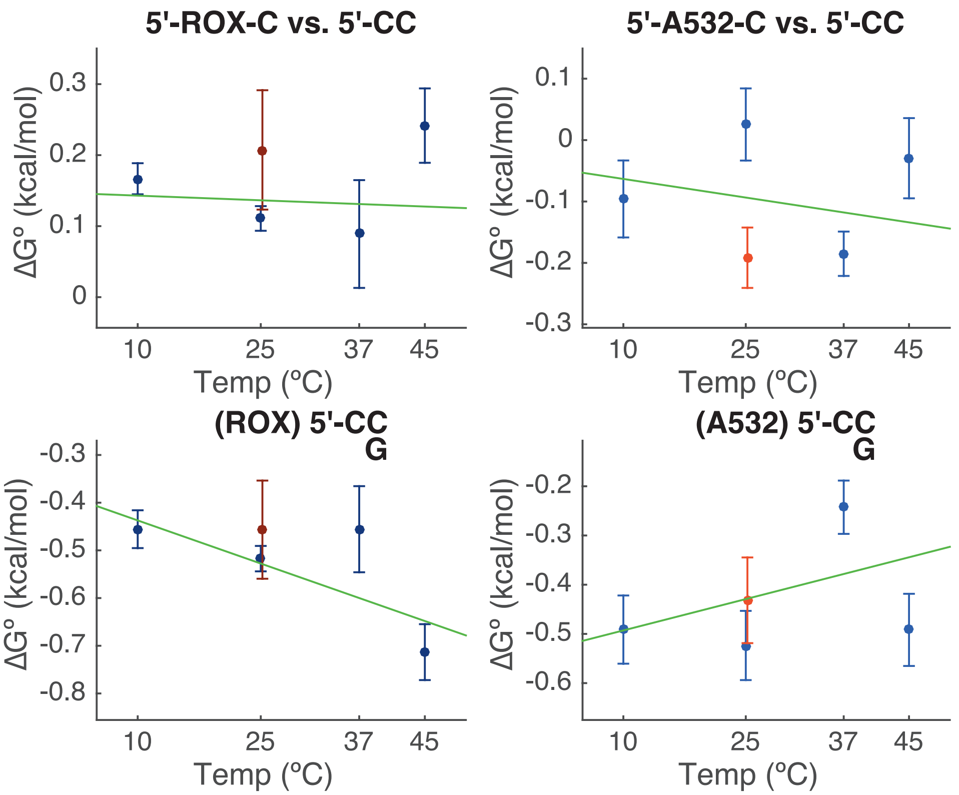

Supplementary Figure 36: Summary of best-fit-raw and real ΔG° values of dangle at four temperatures.

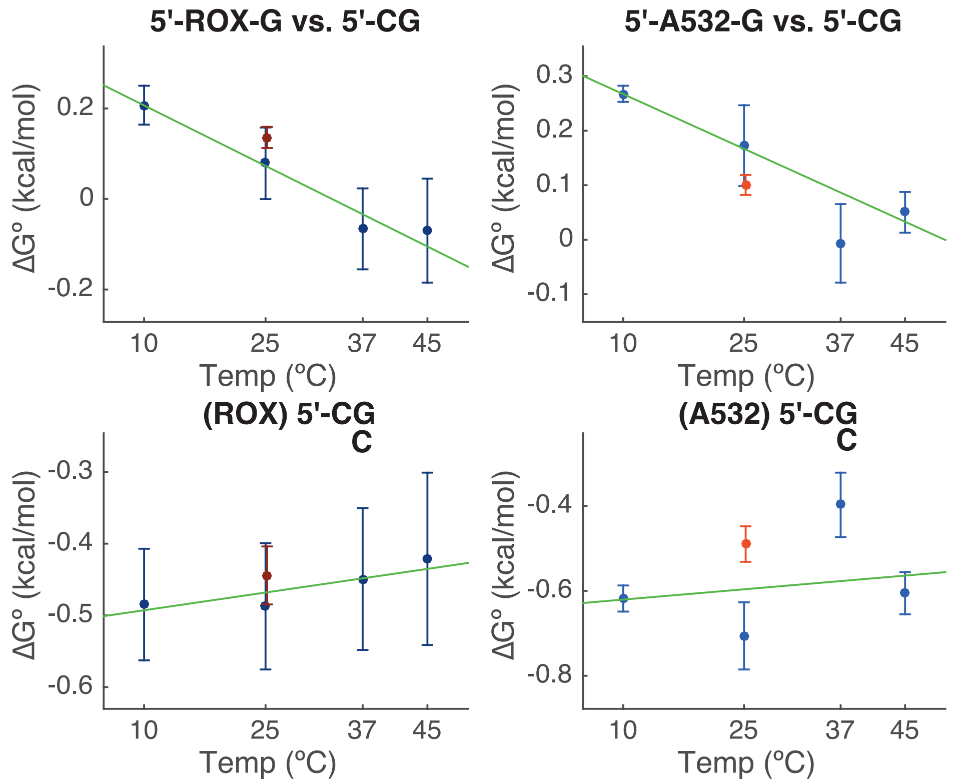

Supplementary Figure 37: Summary of best-fit-raw and real ΔG° values of dangle at four temperatures.

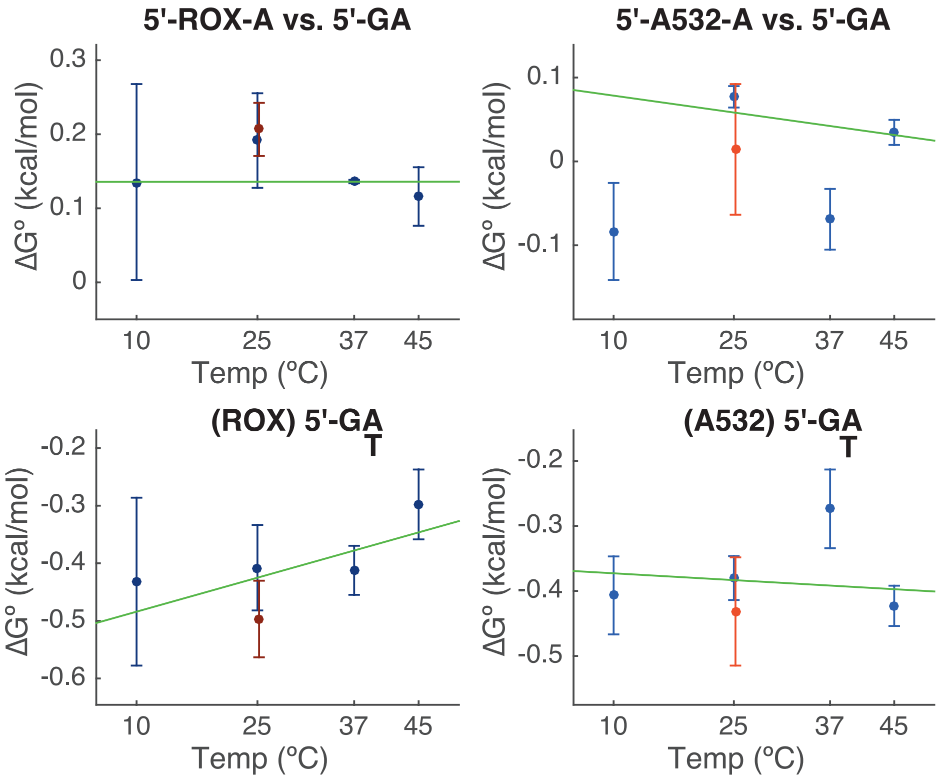

Supplementary Figure 38: Summary of best-fit-raw and real ΔG° values of dangle at four temperatures.

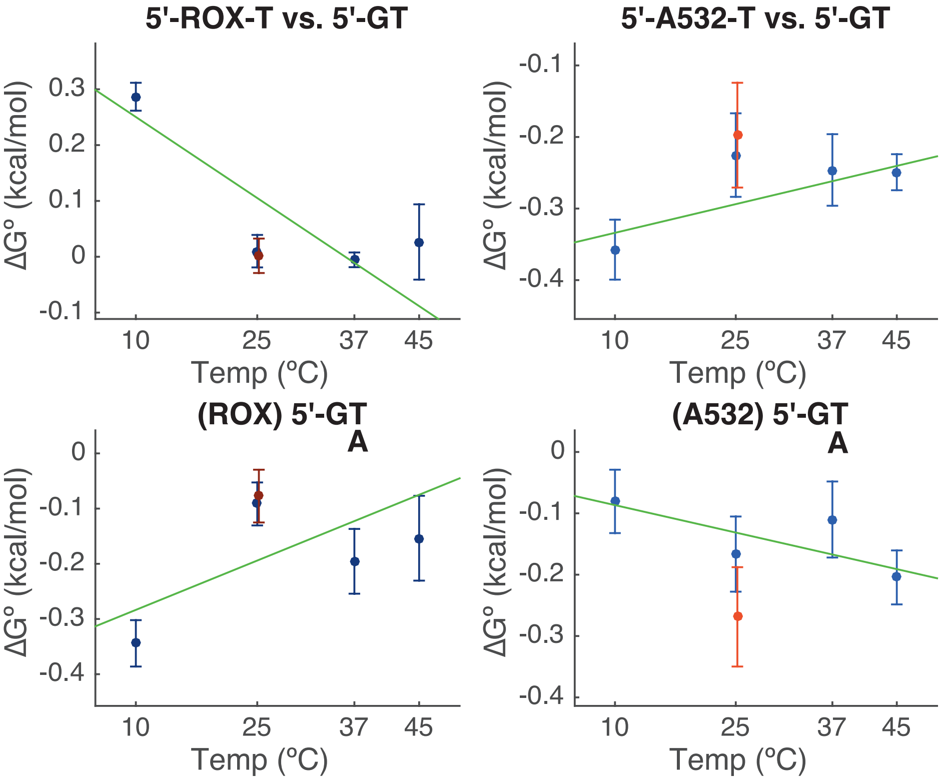

Supplementary Figure 39: Summary of best-fit-raw and real ΔG° values of dangle at four temperatures.

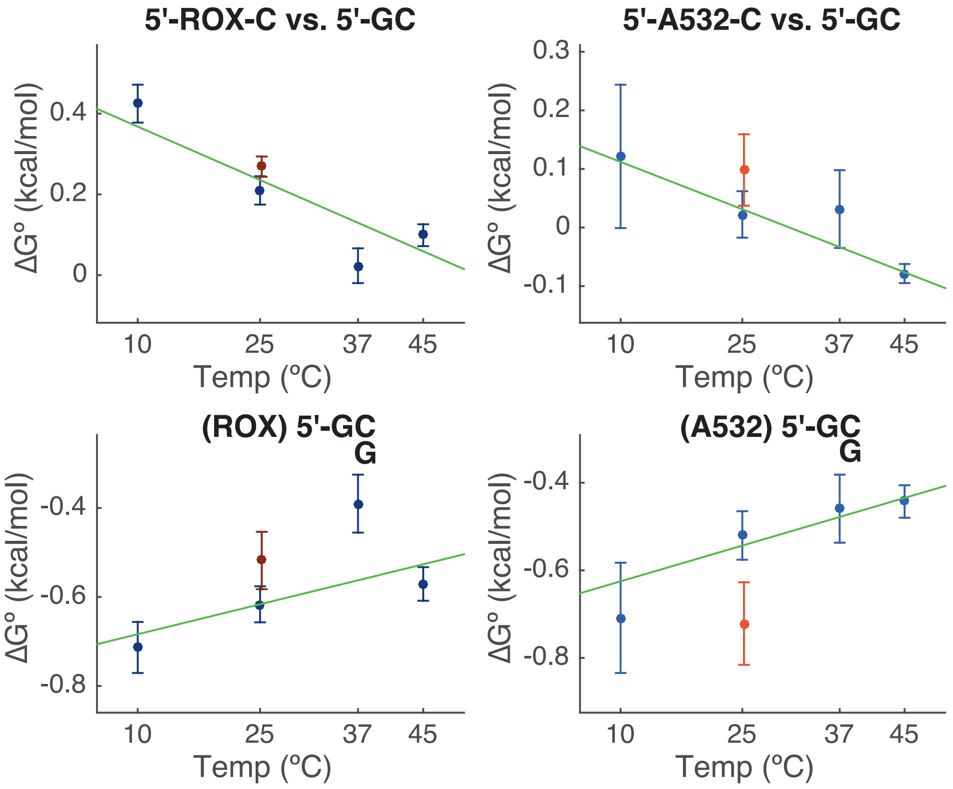

Supplementary Figure 40: Summary of best-fit-raw and real ΔG° values of dangle at four temperatures.

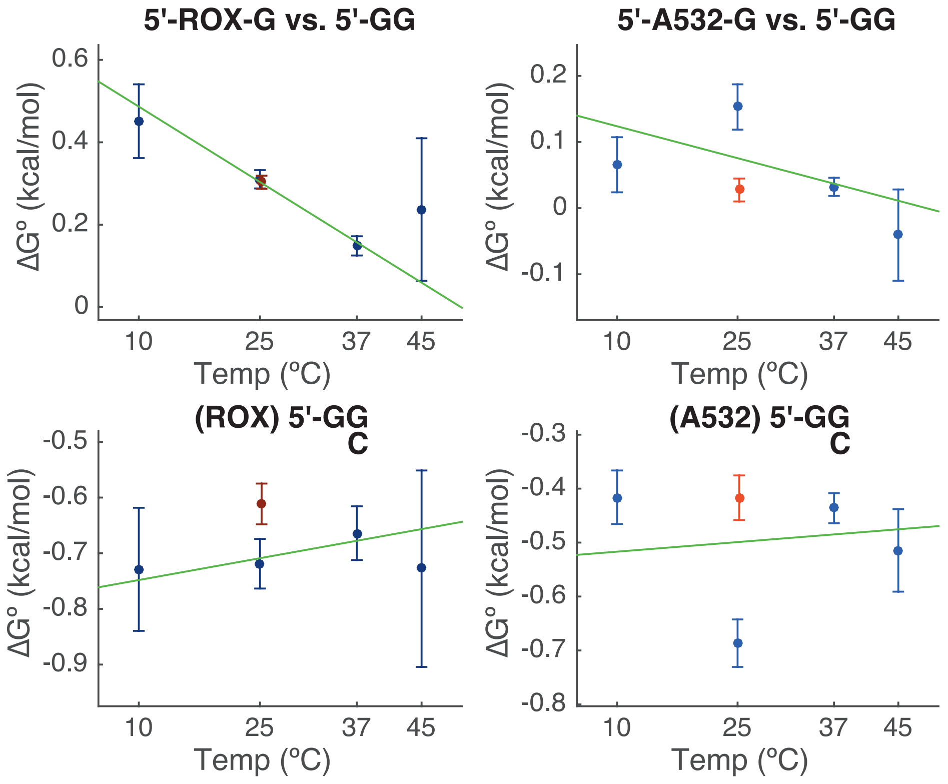

Supplementary Figure 41: Summary of best-fit-raw and real ΔG° values of dangle at four temperatures.

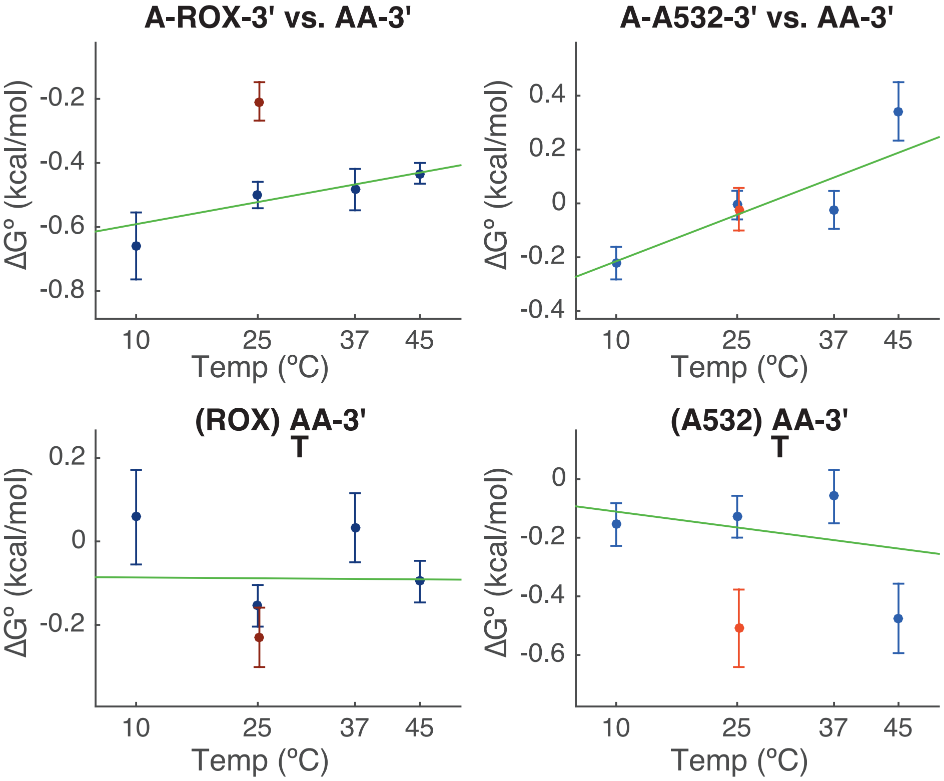

Supplementary Figure 42: Summary of best-fit-raw and real ΔG° values of dangle at four temperatures.

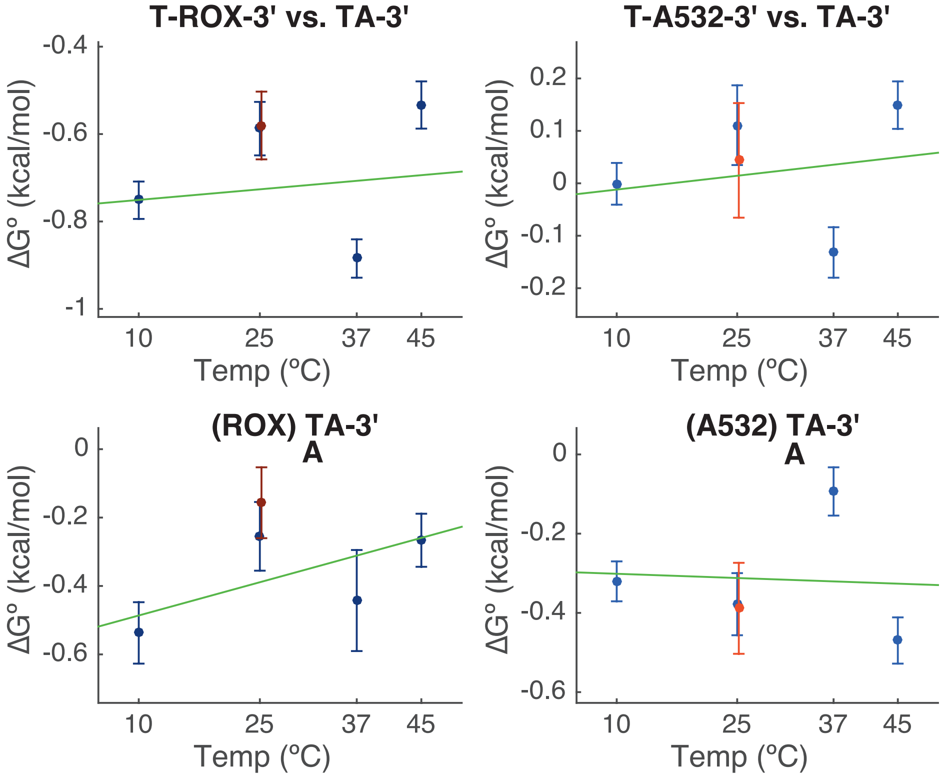

Supplementary Figure 43: Summary of best-fit-raw and real ΔG° values of dangle at four temperatures.

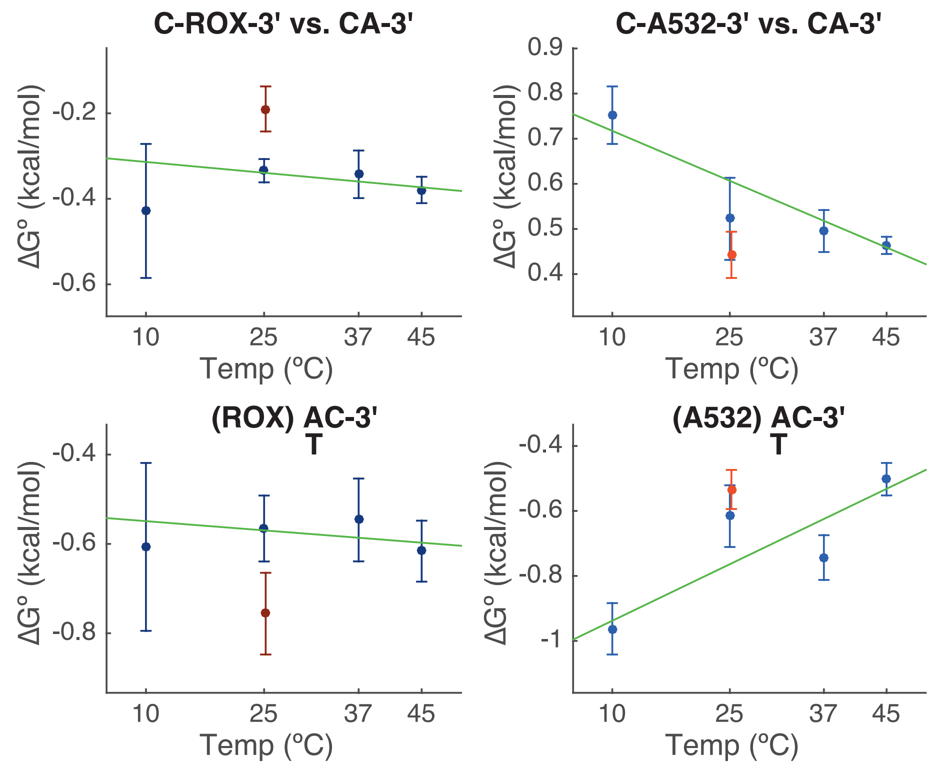

Supplementary Figure 44: Summary of best-fit-raw and real ΔG° values of dangle at four temperatures.

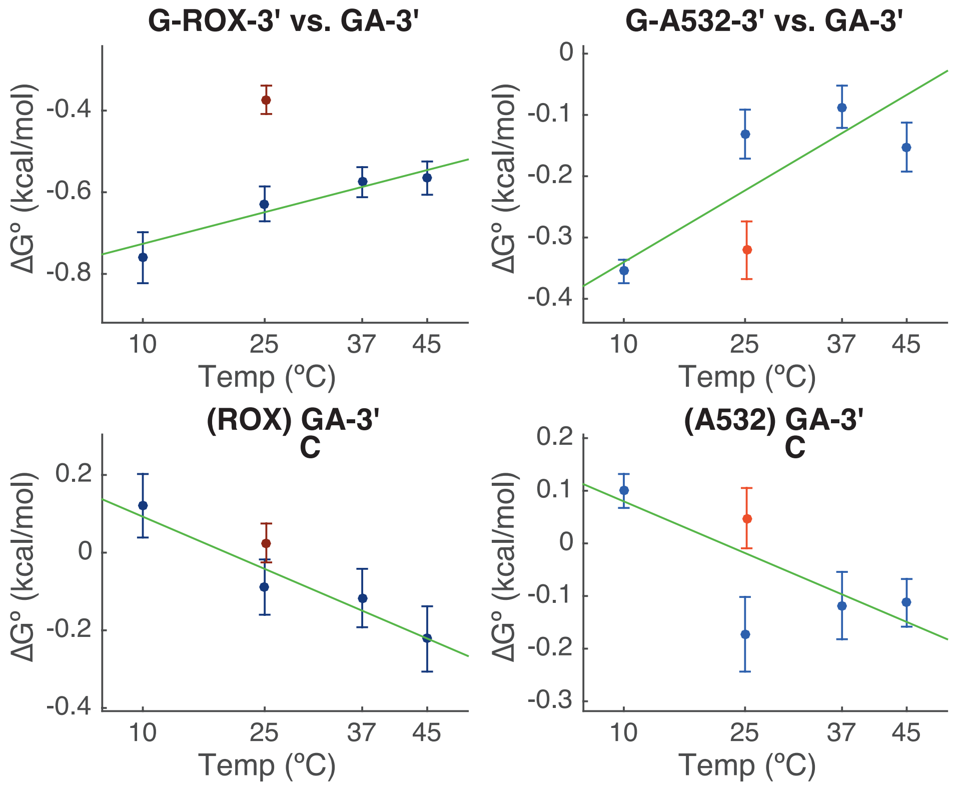

Supplementary Figure 45: Summary of best-fit-raw and real ΔG° values of dangle at four temperatures.

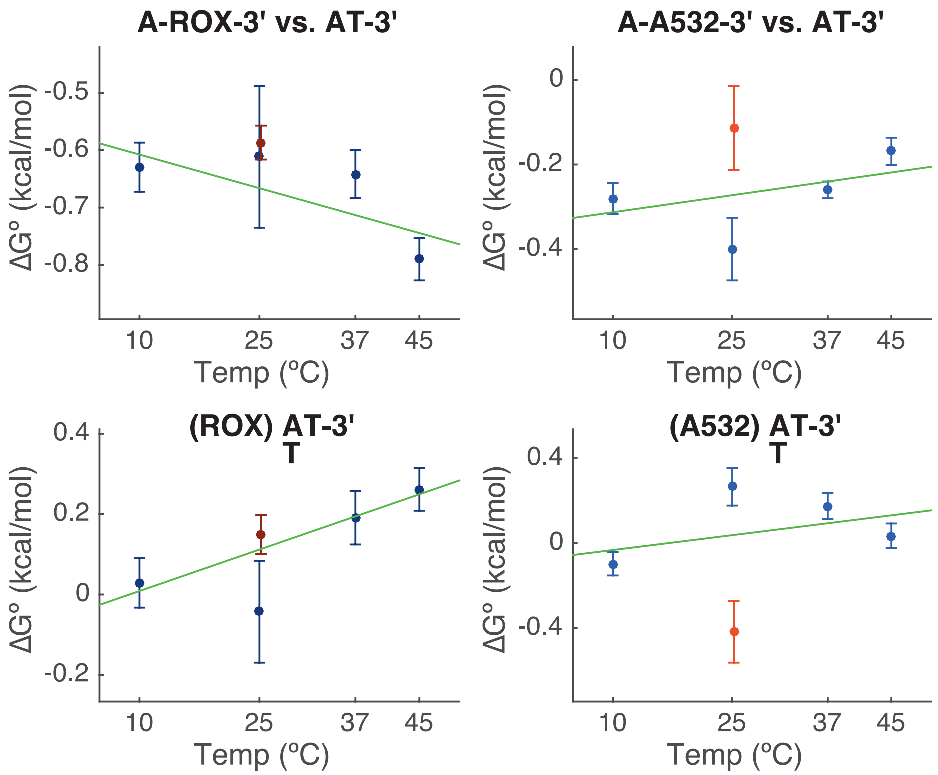

Supplementary Figure 46: Summary of best-fit-raw and real ΔG° values of dangle at four temperatures.

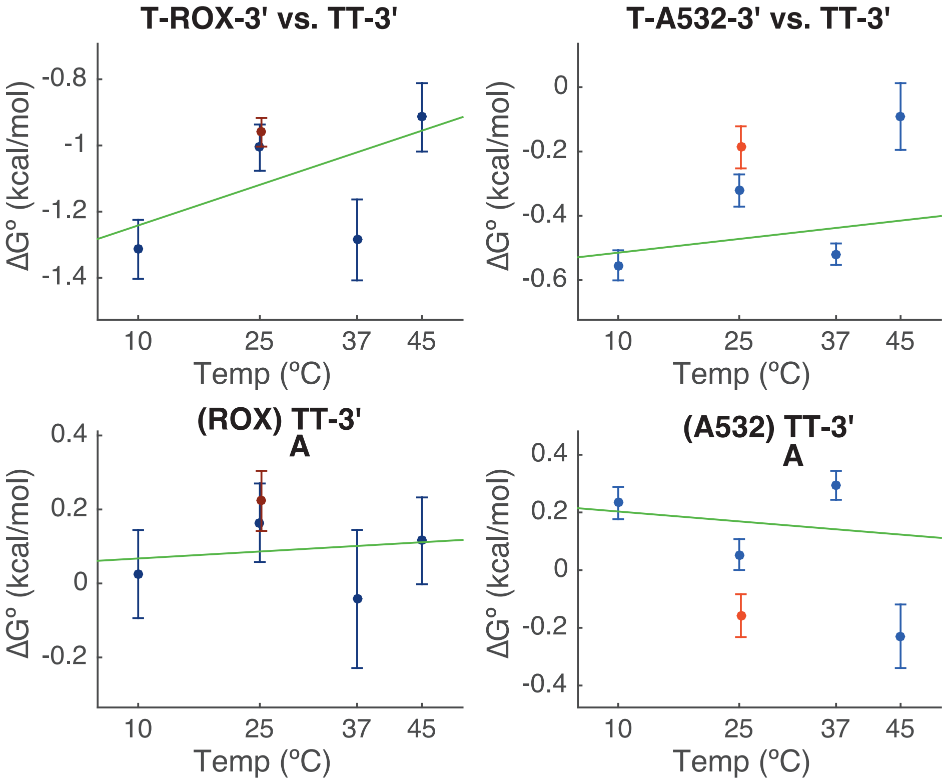

Supplementary Figure 47: Summary of best-fit-raw and real ΔG° values of dangle at four temperatures.

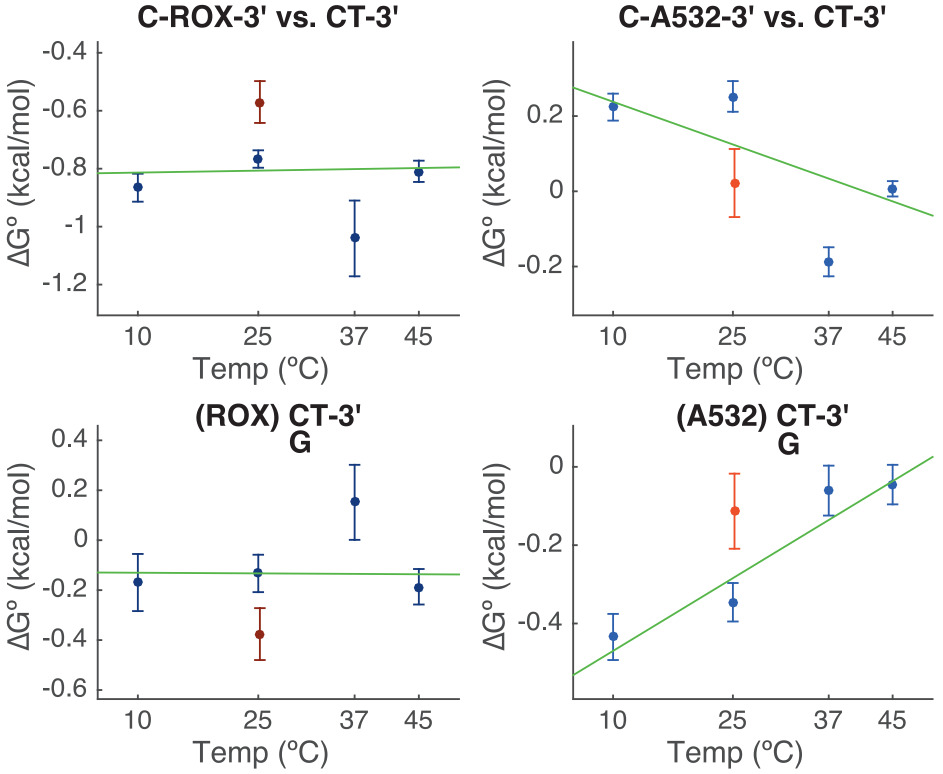

Supplementary Figure 48: Summary of best-fit-raw and real ΔG° values of dangle at four temperatures.

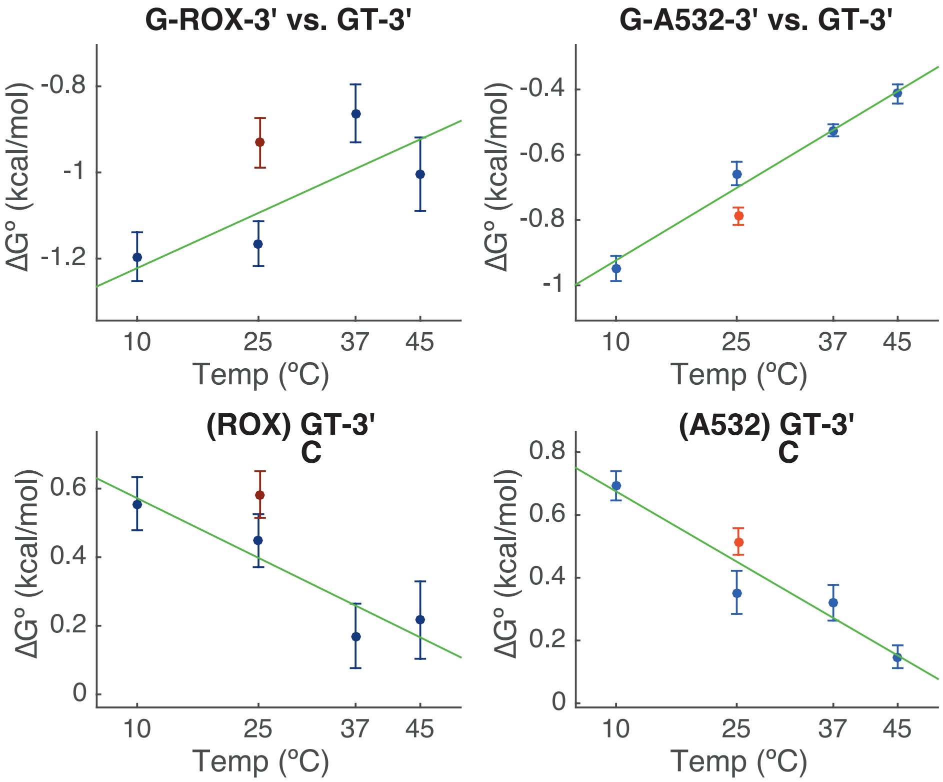

Supplementary Figure 49: Summary of best-fit-raw and real ΔG° values of dangle at four temperatures.

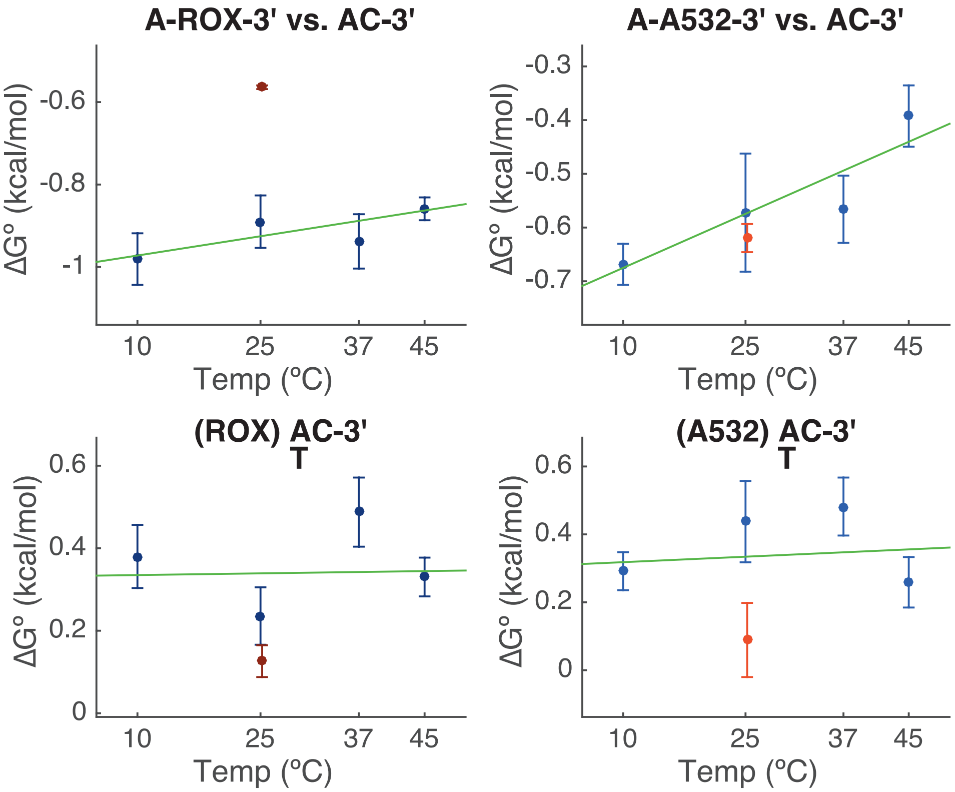

Supplementary Figure 50: Summary of best-fit-raw and real ΔG° values of dangle at four temperatures.

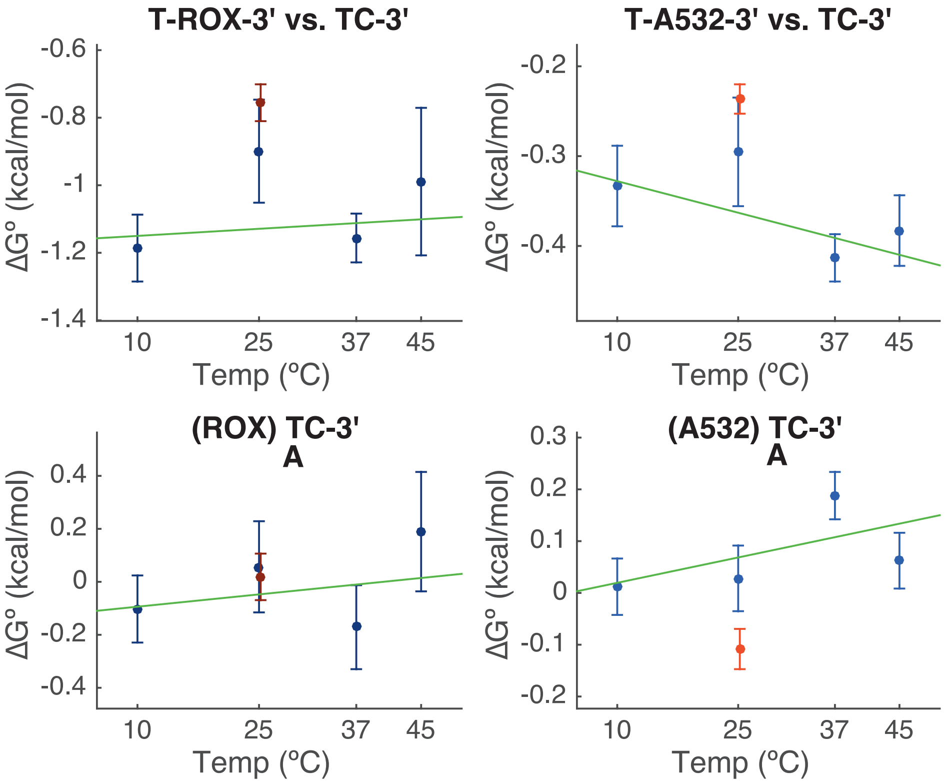

Supplementary Figure 51: Summary of best-fit-raw and real ΔG° values of dangle at four temperatures.

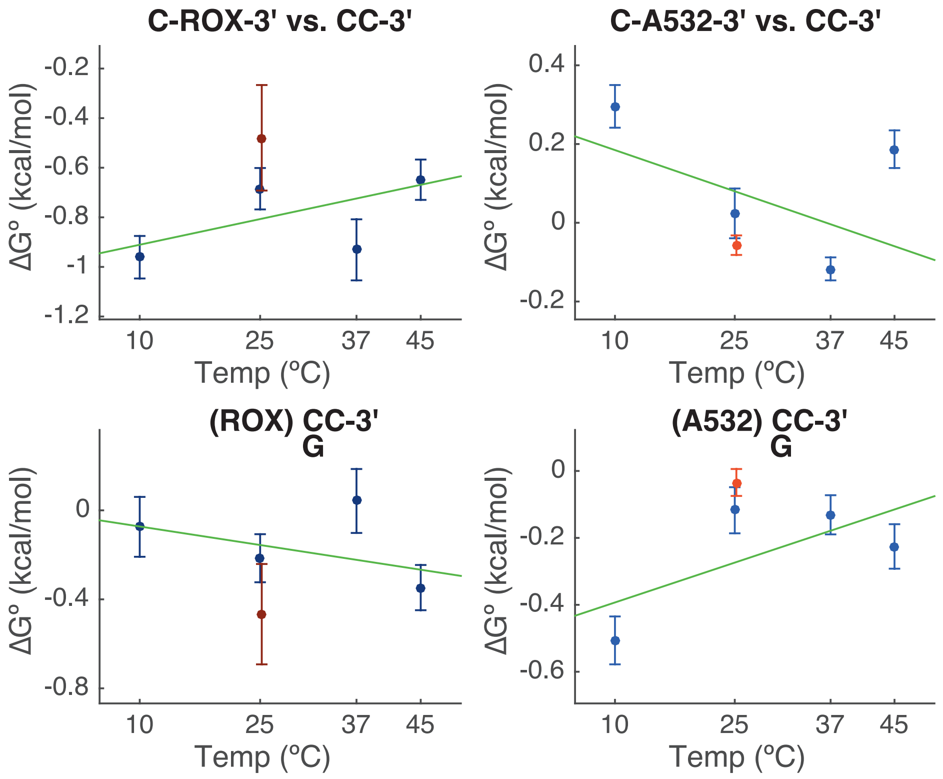

Supplementary Figure 52: Summary of best-fit-raw and real ΔG° values of dangle at four temperatures.

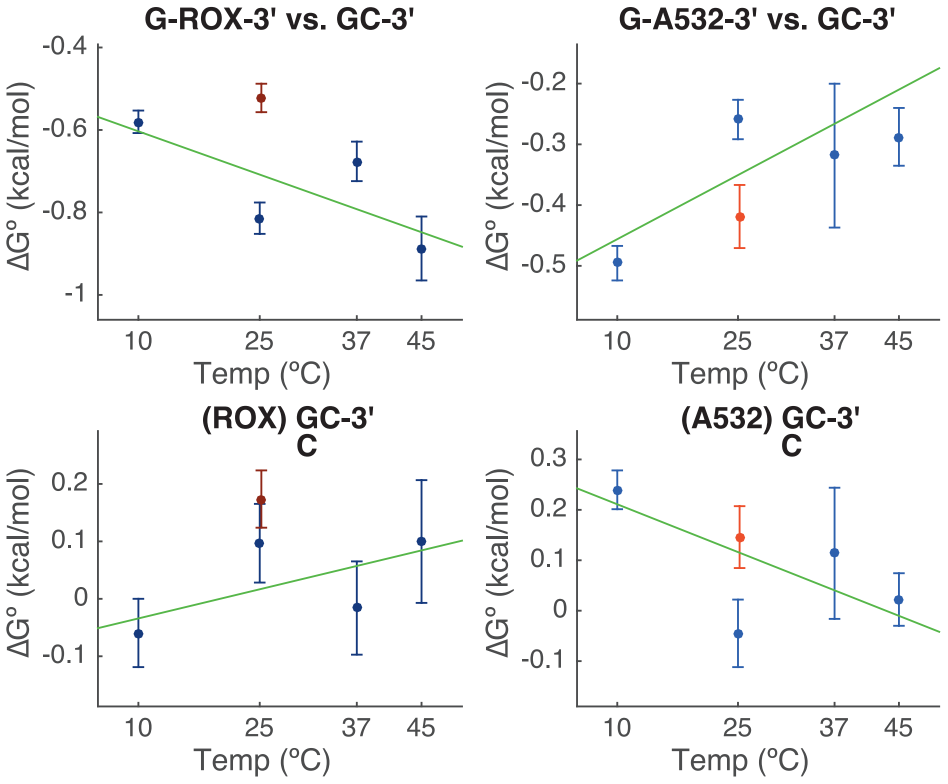

Supplementary Figure 53: Summary of best-fit-raw and real ΔG° values of dangle at four temperatures.

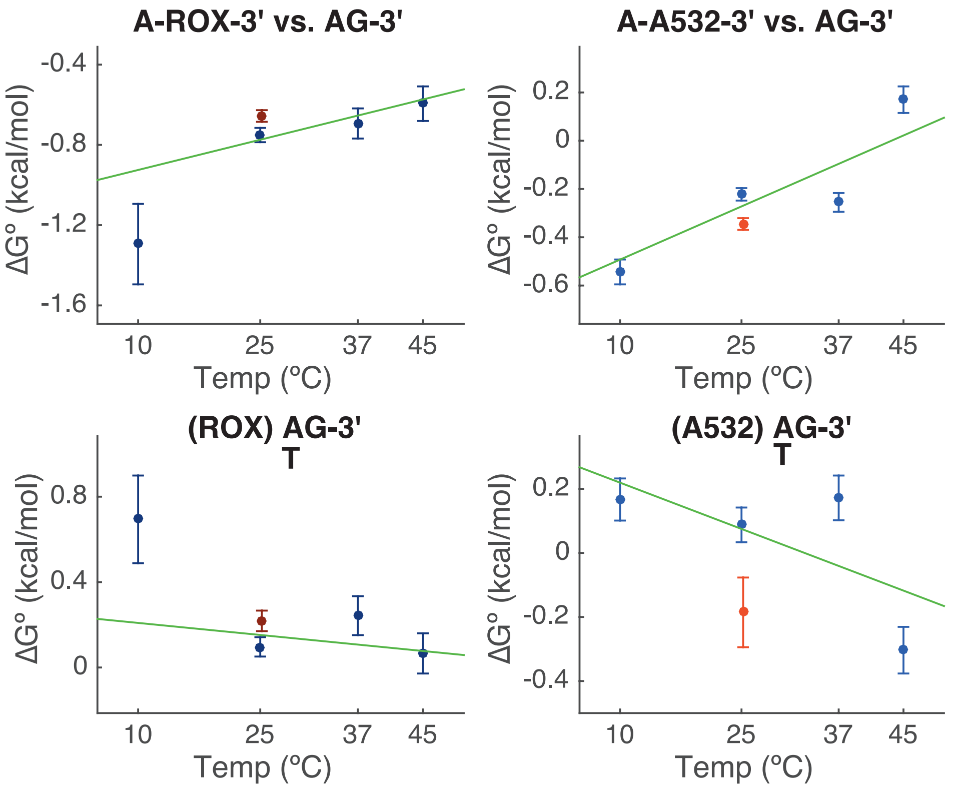

Supplementary Figure 54: Summary of best-fit-raw and real ΔG° values of dangle at four temperatures.

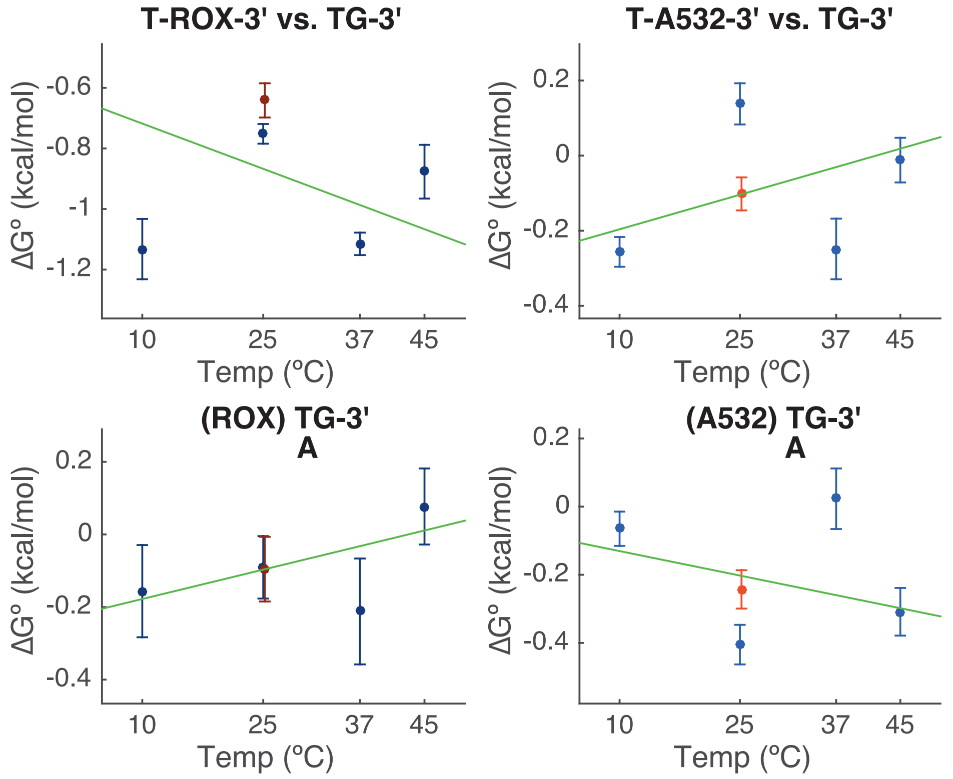

Supplementary Figure 55: Summary of best-fit-raw and real ΔG° values of dangle at four temperatures.

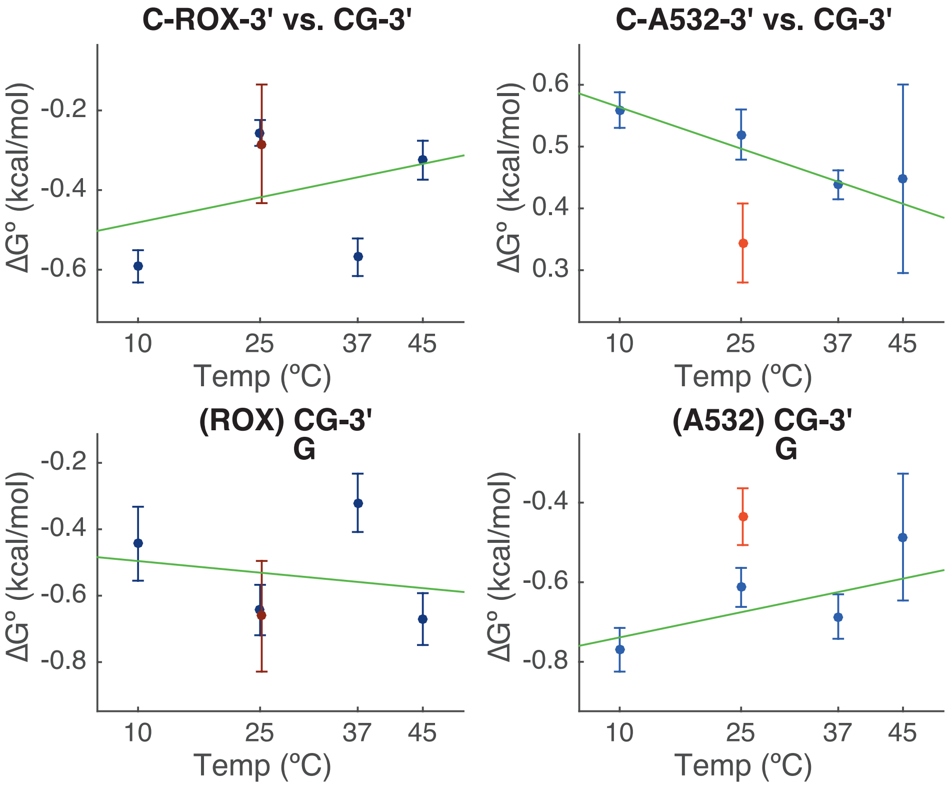

Supplementary Figure 56: Summary of best-fit-raw and real ΔG° values of dangle at four temperatures.

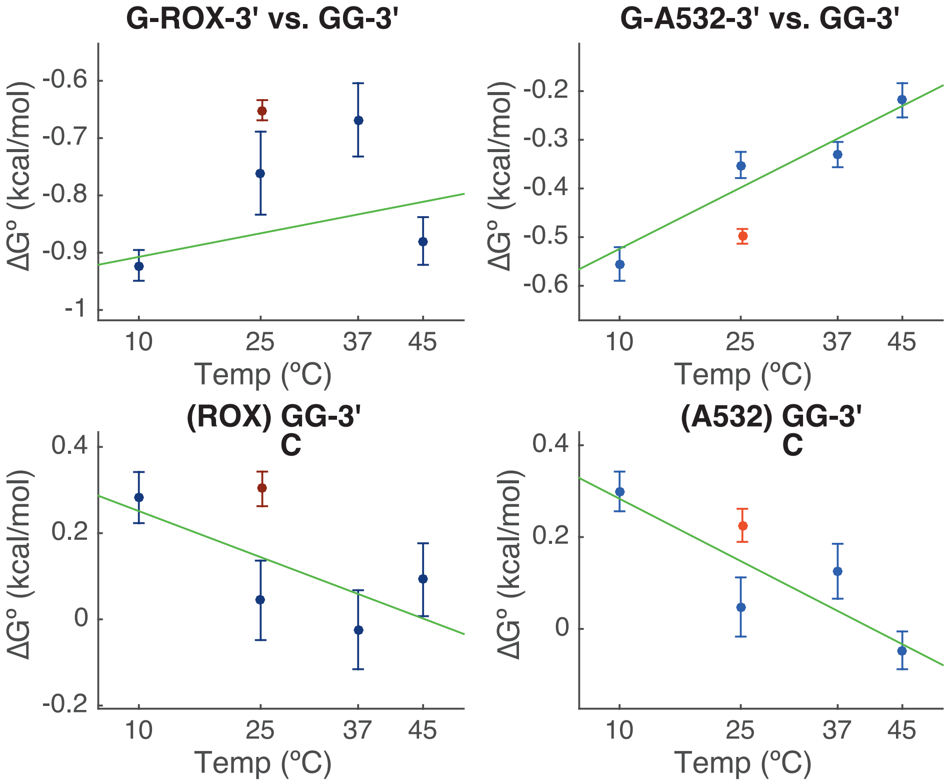

Supplementary Figure 57: Summary of best-fit-raw and real ΔG° values of dangle at four temperatures.

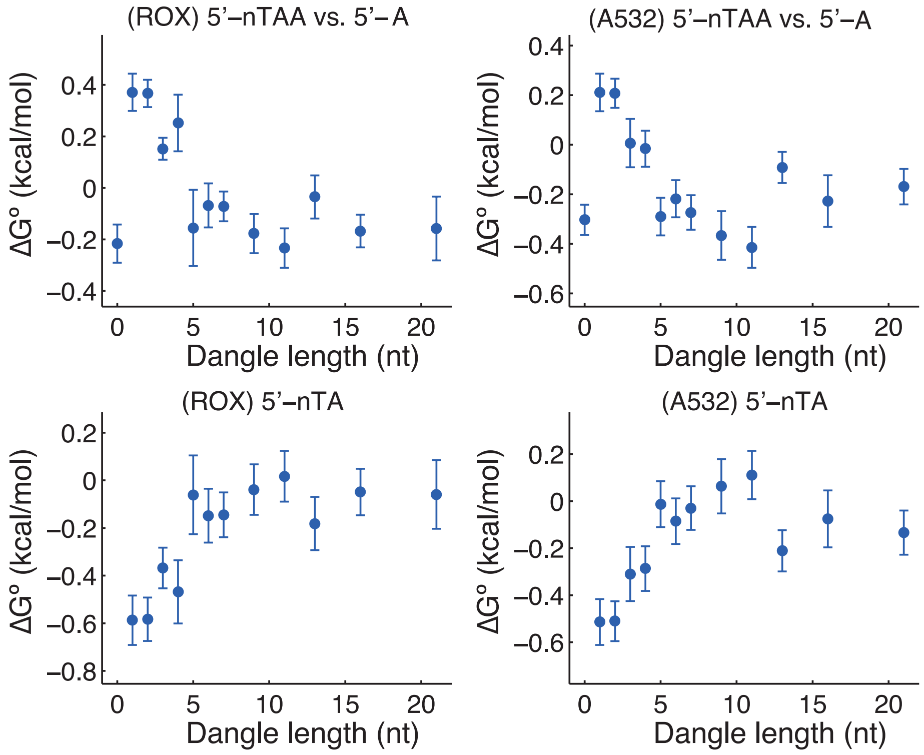

Supplementary Figure 58: Measured raw ΔG° (top panels) and calculated real ΔG° of multibase dangles (bottom panels).

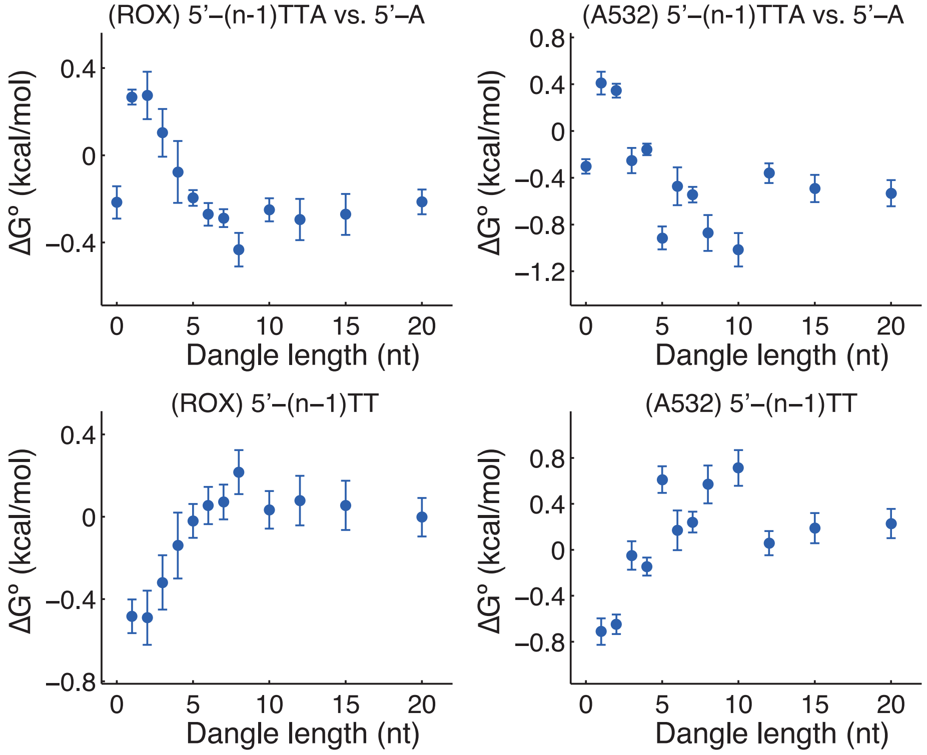

Supplementary Figure 59: Measured raw ΔG° (top panels) and calculated real ΔG° of multibase dangles (bottom panels).

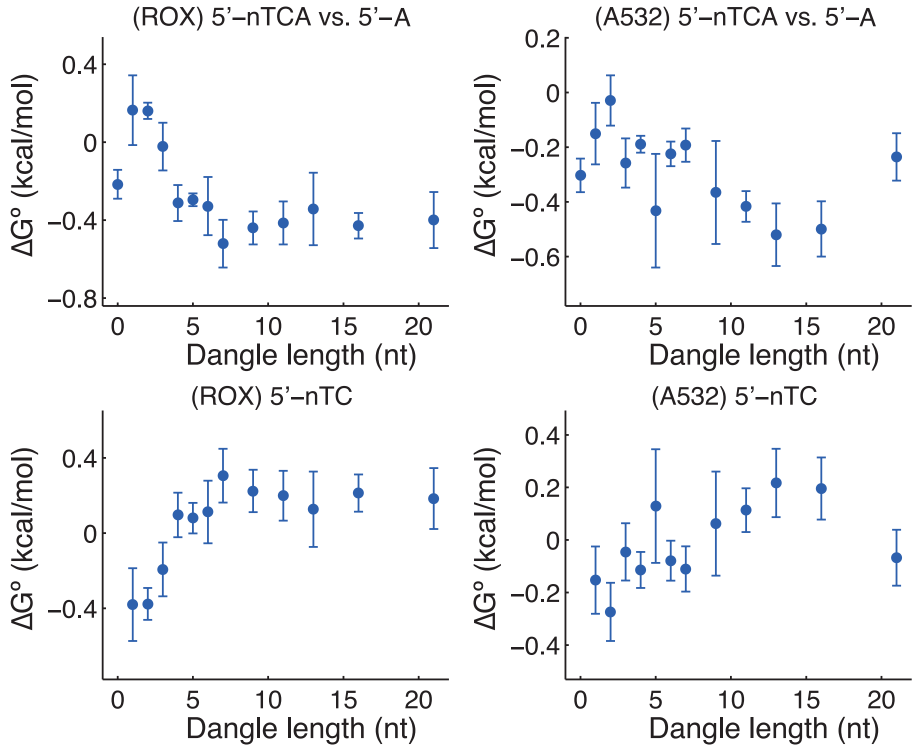

Supplementary Figure 60: Measured raw ΔG° (top panels) and calculated real ΔG° of multibase dangles (bottom panels).

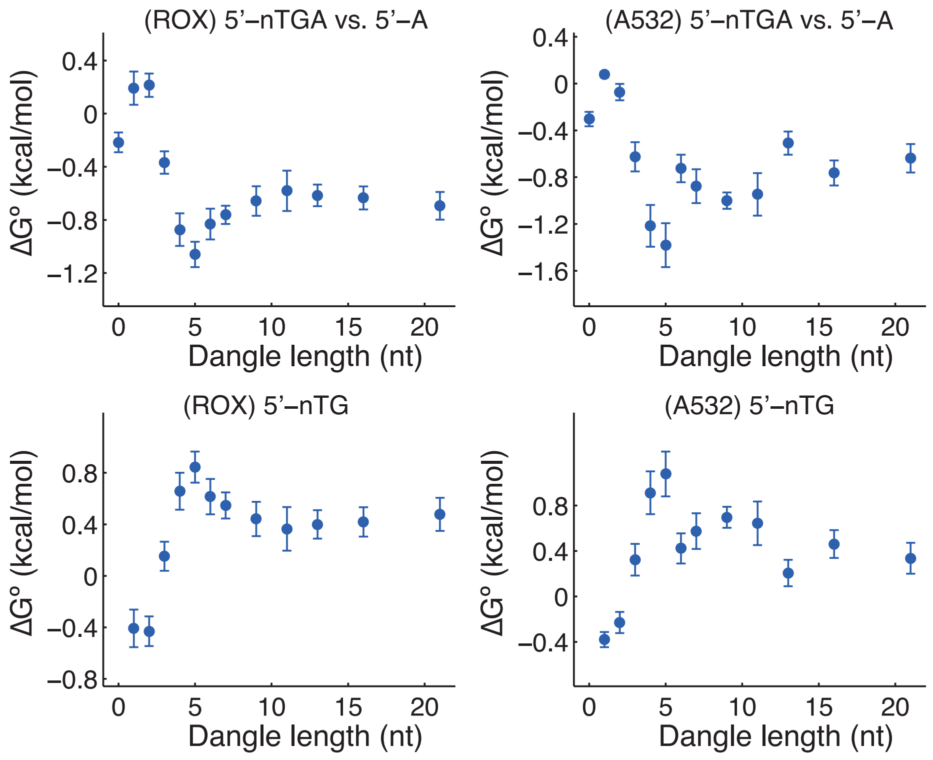

Supplementary Figure 61: Measured raw ΔG° (top panels) and calculated real ΔG° of multibase dangles (bottom panels).

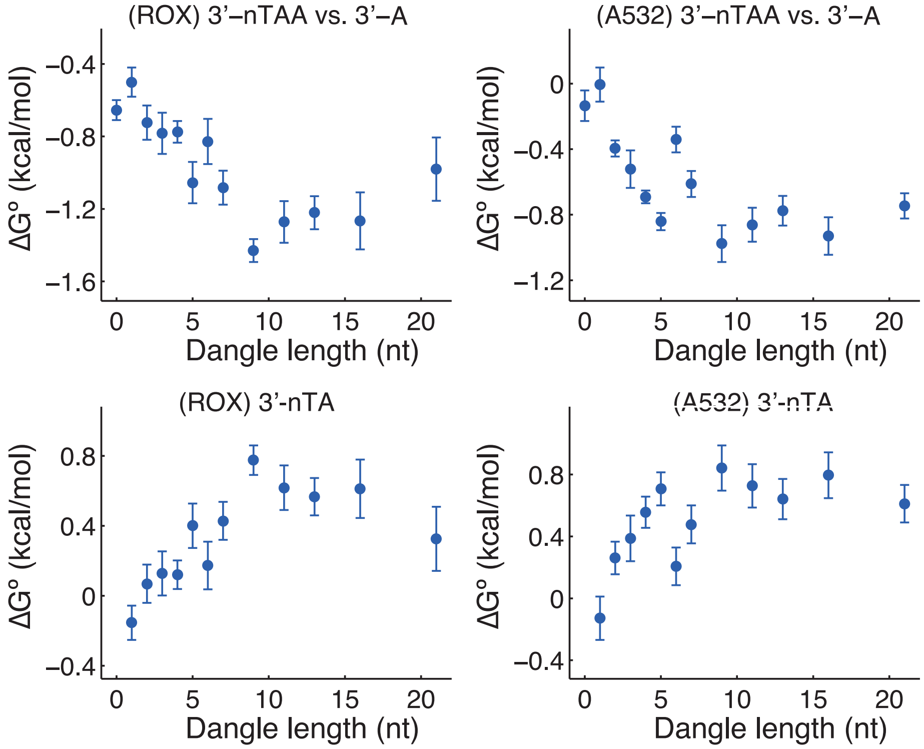

Supplementary Figure 62: Measured raw $\Delta G°$ (top panels) and calculated real $\Delta G°$ of multibase dangles (bottom panels).

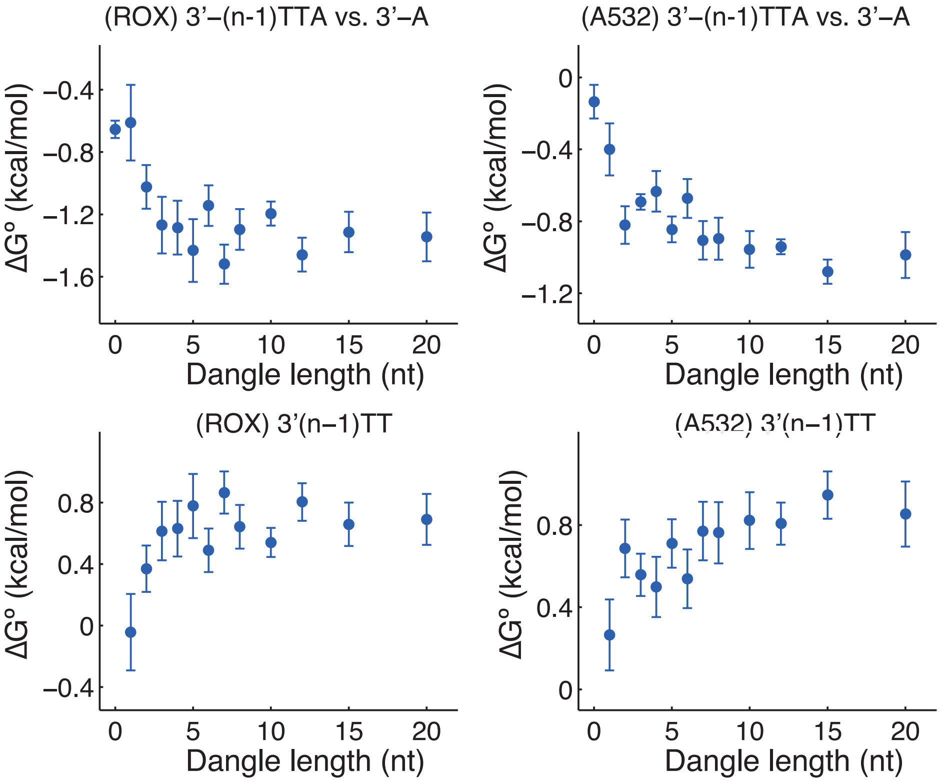

Supplementary Figure 63: Measured raw $\Delta G°$ (top panels) and calculated real $\Delta G°$ of multibase dangles (bottom panels).

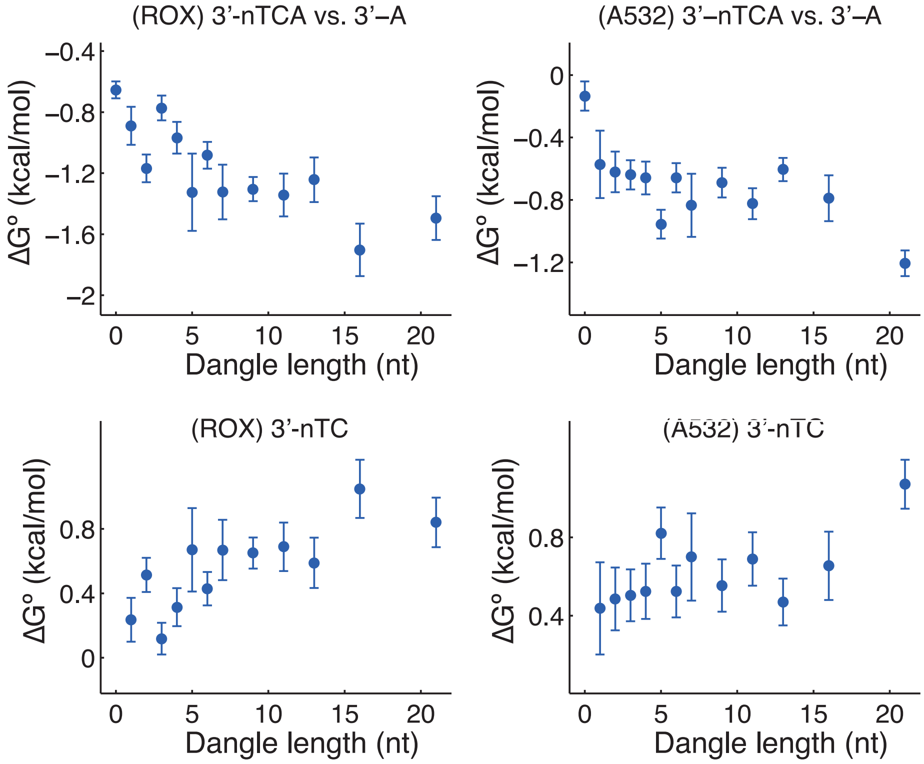

Supplementary Figure 64: Measured raw ΔG° (top panels) and calculated real ΔG° of multibase dangles (bottom panels).

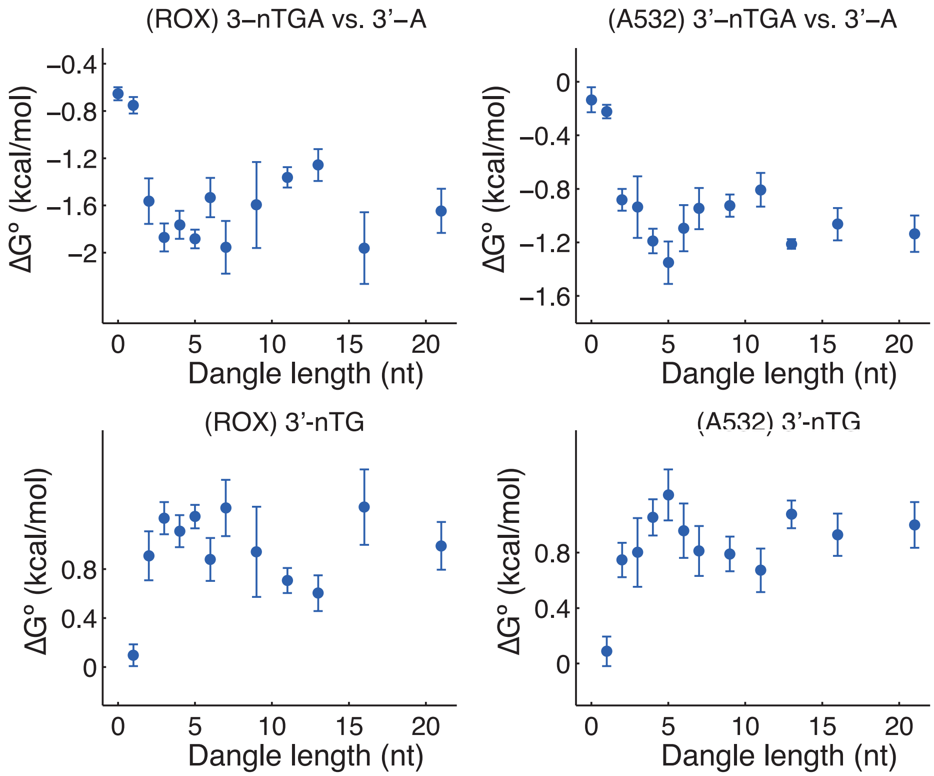

Supplementary Figure 65: Measured raw ΔG° (top panels) and calculated real ΔG° of multibase dangles (bottom panels).

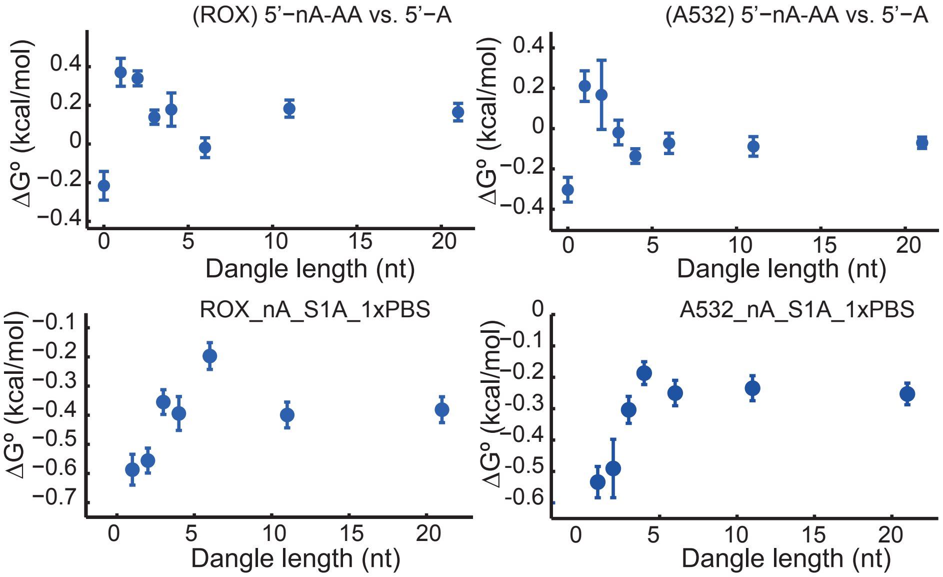

Supplementary Figure 66: Measured raw ΔG° (top panels) and calculated real ΔG° of multibase dangles (bottom panels). Multibase dangles were chose to be adenines in this group of experiments.

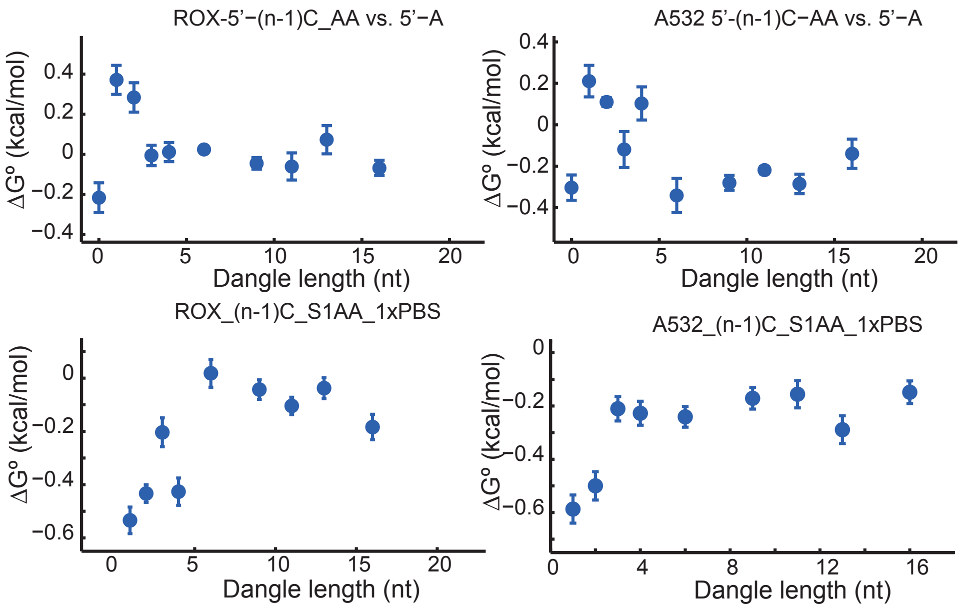

Supplementary Figure 67: Measured raw ΔG° (top panels) and calculated real ΔG° of multibase dangles (bottom panels). Multibase dangles were chose to be cytosine bases adjacent to one adenine in this group of experiments.

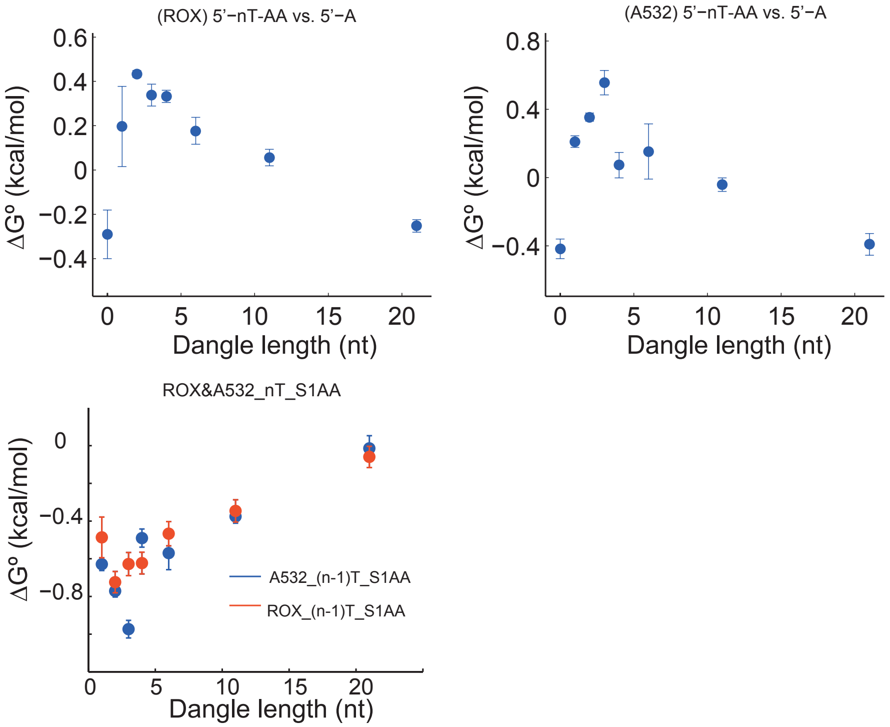

Supplementary Figure 68: Measured raw ΔG° (top panels) and calculated real ΔG° of multibase dangles (bottom panels) in Tris-Mg buffer at 25°C with thymine bases adjacent one adenine as dangles.

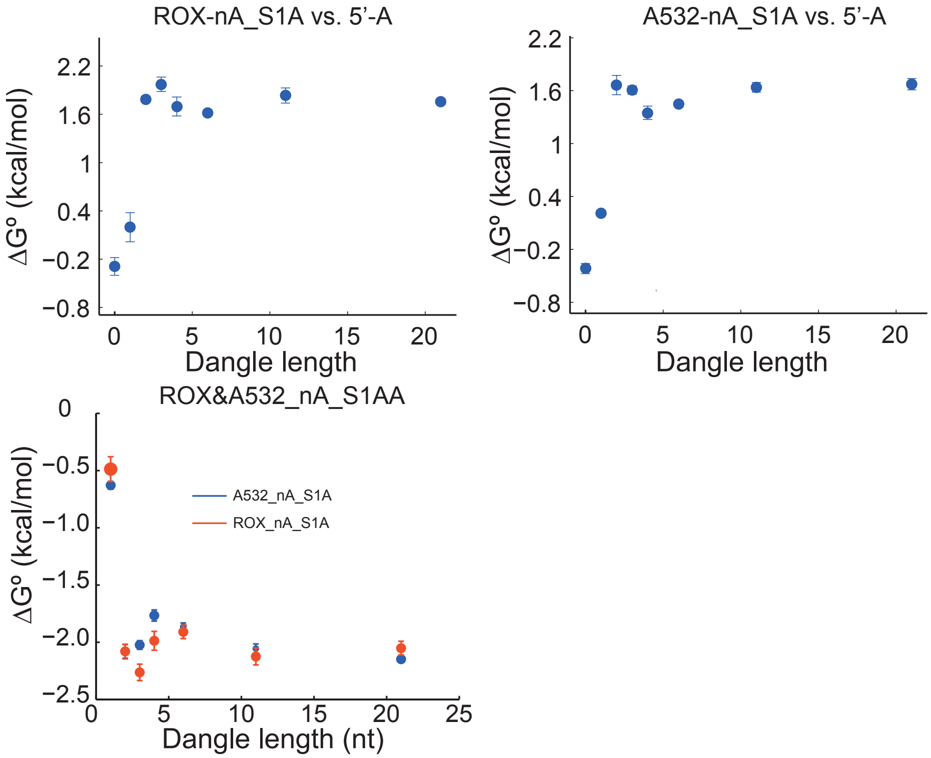

Supplementary Figure 69: Measured raw ΔG° (top panels) and calculated real ΔG° of multibase dangles (bottom panels) in Tris-Mg buffer at 25°C with adenine bases as dangles.

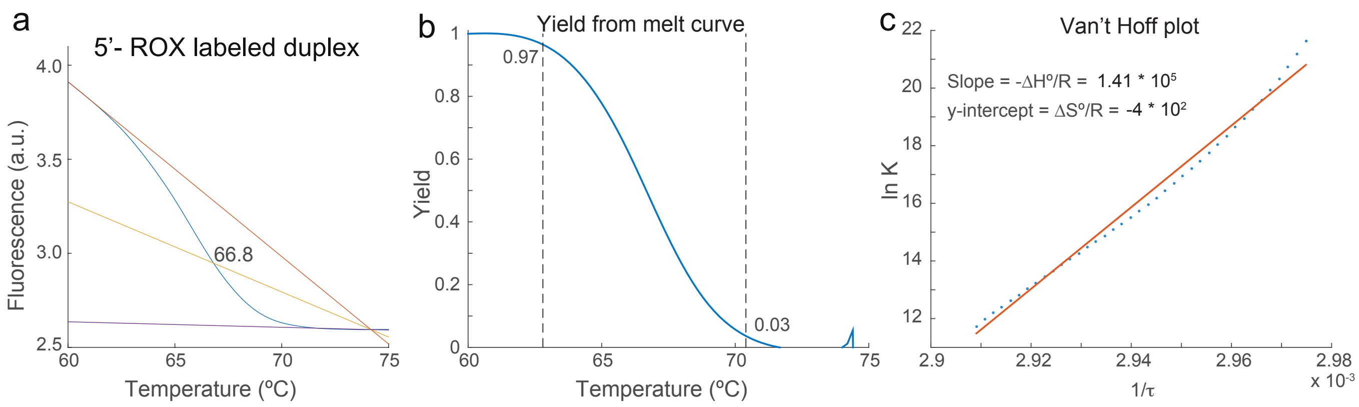

Supplementary Figure 70: Thermodynamic parameter inference from melt curves. **(a)** Melt curve (blue), upper baseline (red), and lower baseline (purple). The yellow line is the average of the upper and lower baselines; where the blue melt curve intersects the yellow line is taken to be the melting temperature of the duplex. **(b)** Yield is calculated from a linear transformation of the observed fluorescence, based on the upper and lower baselines. **(c)** Equilibrium constant K is calculated from yield, and the natural logarithm of K is plotted against the inverse of the temperature τ (in Kelvin). The slope and intercept of the best linear fit to the data gives the inferred ΔH° and ΔS°, respectively.

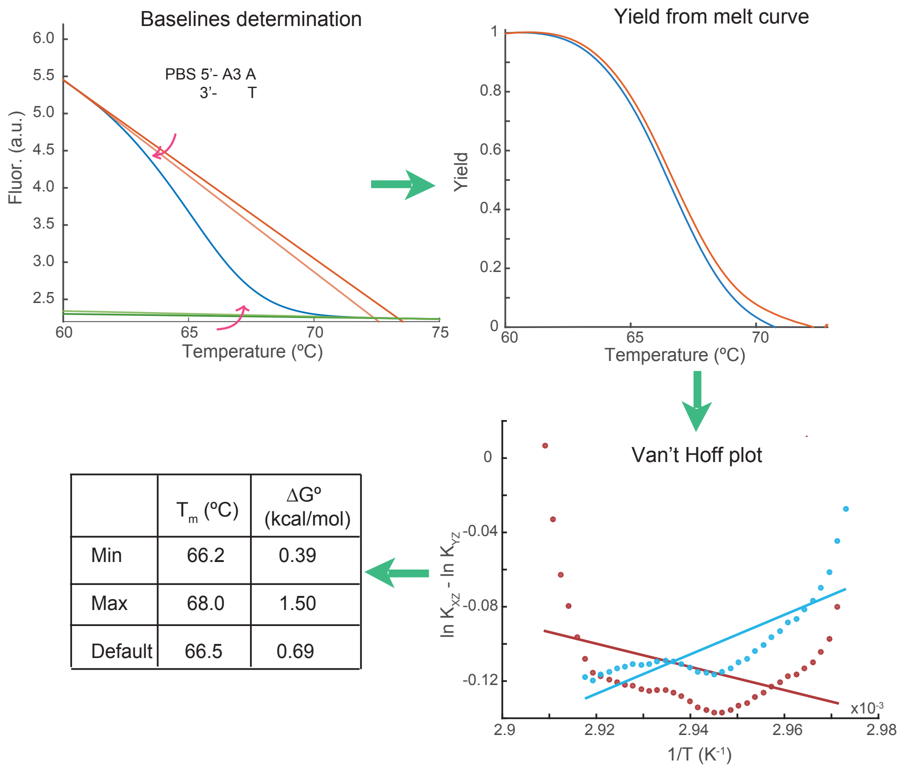

	T_m (ºC)	ΔGº (kcal/mol)
Min	66.2	0.39
Max	68.0	1.50
Default	66.5	0.69

Supplementary Figure 71: Details of fitting the upper and lower baselines affect inferred ΔH° and ΔS° values, here, of a 3nt AAA 5′ dangle. **(a)** Reasonable fits for upper and lower baselines can vary slightly, depending on the number of data points used to fit the baselines. **(b)** The slight changes in upper and lower baselines cause a larger change in the yield vs. temperature plot. **(c)** The Van't Hoff plot is similarly affected. Thus, for the same exact melt curve, reasonable assumptions on upper and lower baselines can result in inferred motif thermodynamics differing by up to 1.12 kcal/mol at 25 °C.

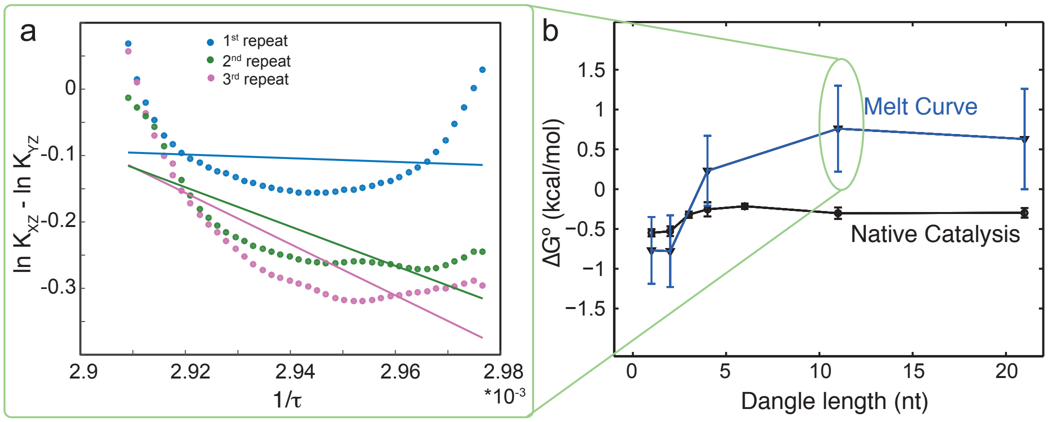

Supplementary Figure 72: Motif ΔG° inferred from melt curves generally show high variability, resulting in wide error bars. **(a)** Van't Hoff plots for triplicate melt curves. **(b)** Comparing iferred ΔG° values of multinucleotide dangles at 25 °C. Native catalysis technique produces much smaller standard deviations. Error bars show 1 standard deviation.

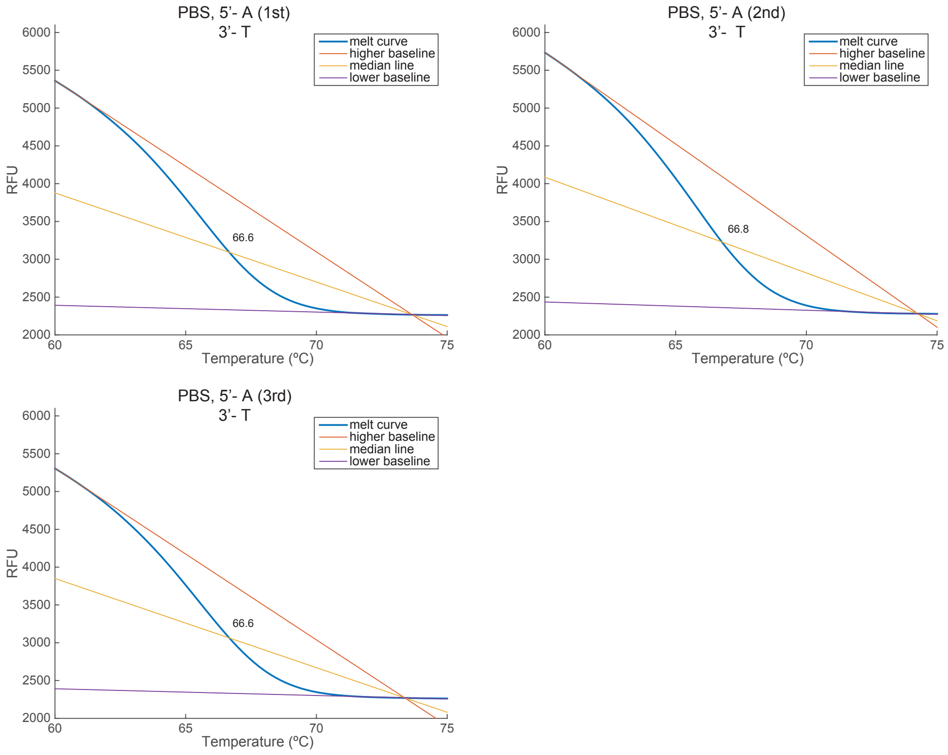

Supplementary Figure 73: Melt curves of the reference duplex with an A/T closing base pair and no dangle, in 1x PBS buffer.

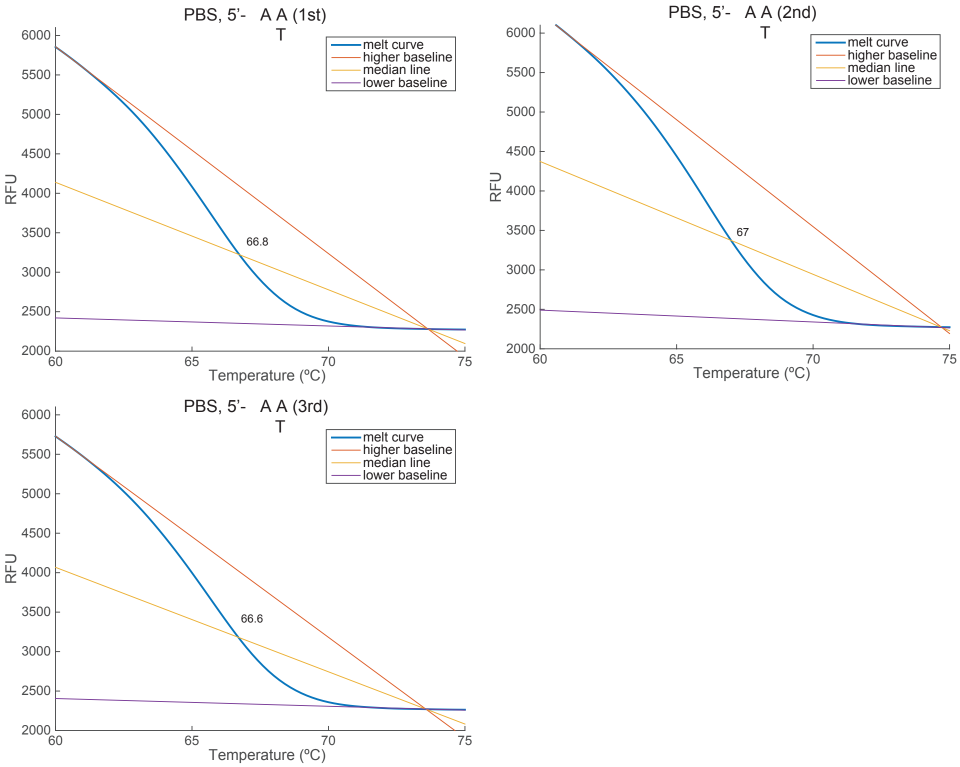

Supplementary Figure 74: Melt curves of 5′ 1nt A dangle next to closing base A in 1x PBS buffer.

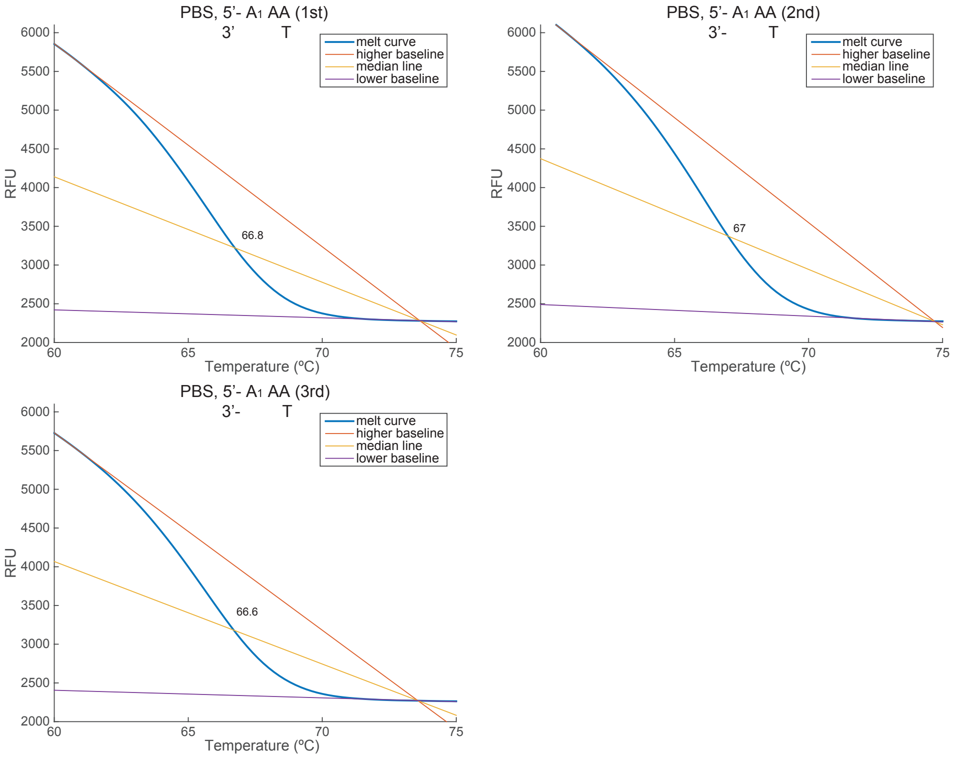

Supplementary Figure 75: Melt curves of 5′ 2nt AA dangle next to closing base A in 1x PBS buffer.

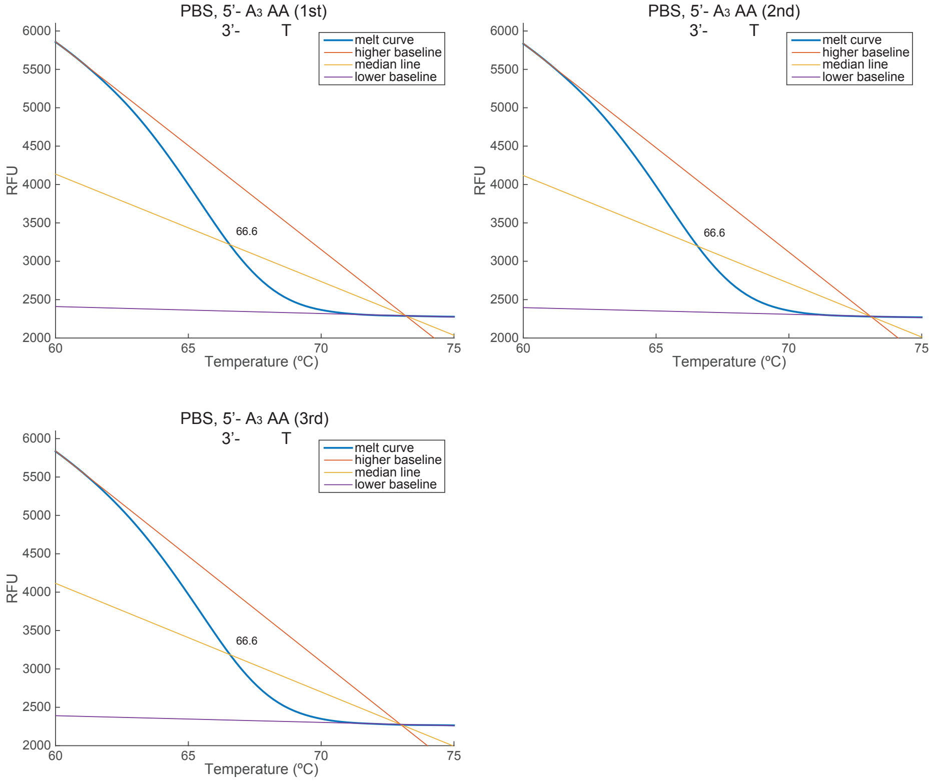

Supplementary Figure 76: Melt curves of 5′ 4nt A_4 dangle next to closing base A in 1x PBS buffer.

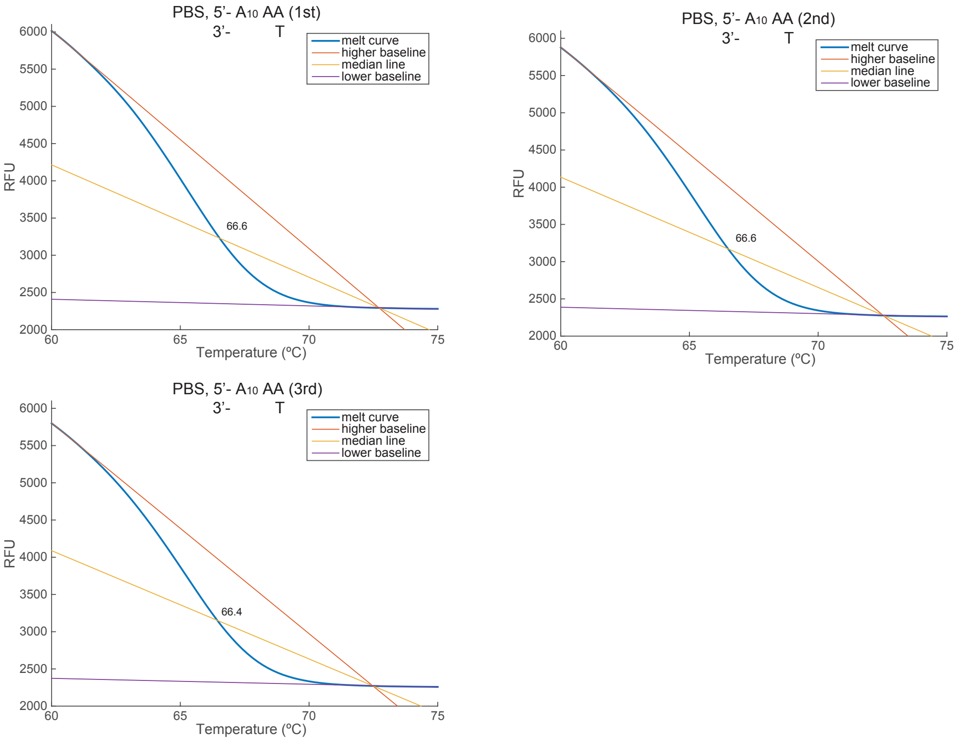

Supplementary Figure 77: Melt curves of 5′ 10nt A_{10} dangle next to closing base A in 1x PBS buffer.

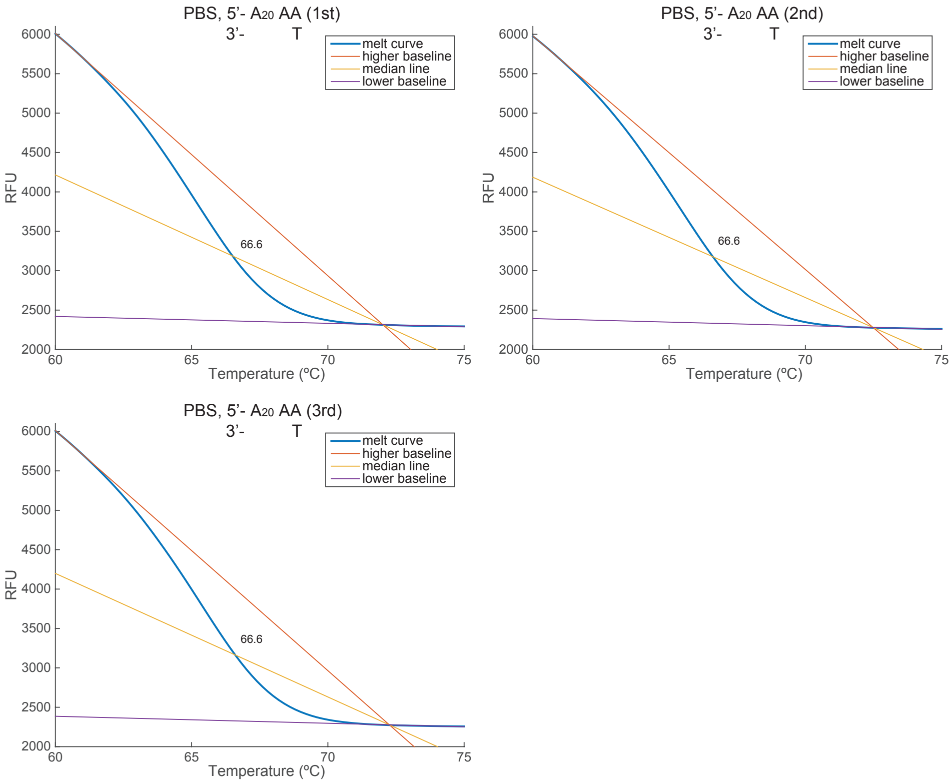

Supplementary Figure 78: Melt curves of 5′ 20nt A_{20} dangle next to closing base A in 1x PBS buffer.

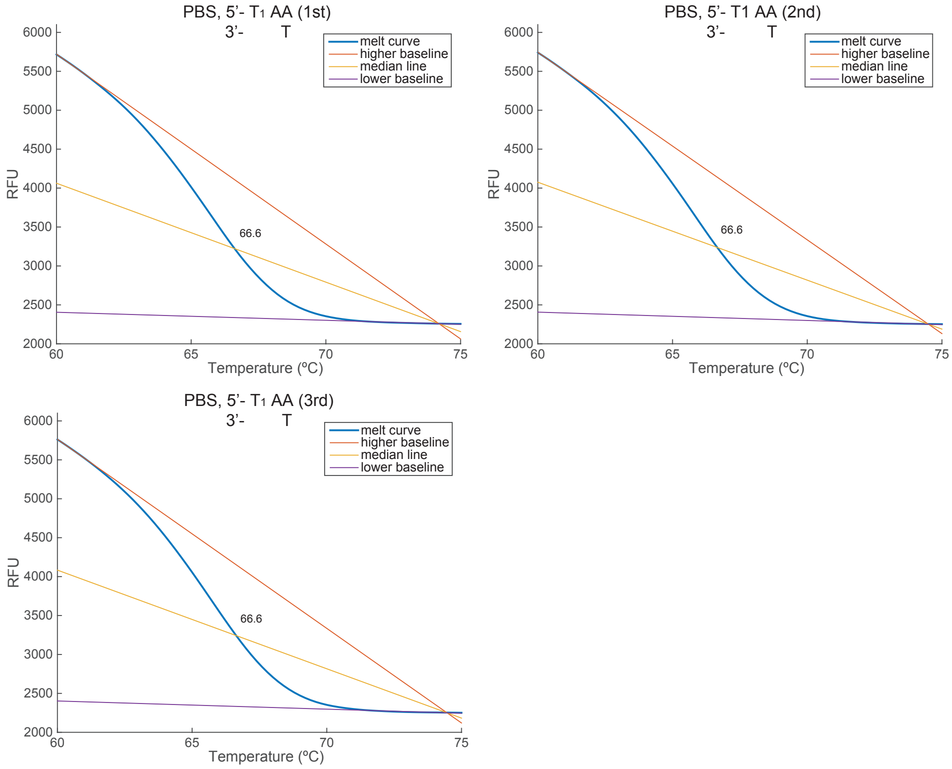

Supplementary Figure 79: Melt curves of 5′ 2nt TA dangle next to closing base A in 1x PBS buffer.

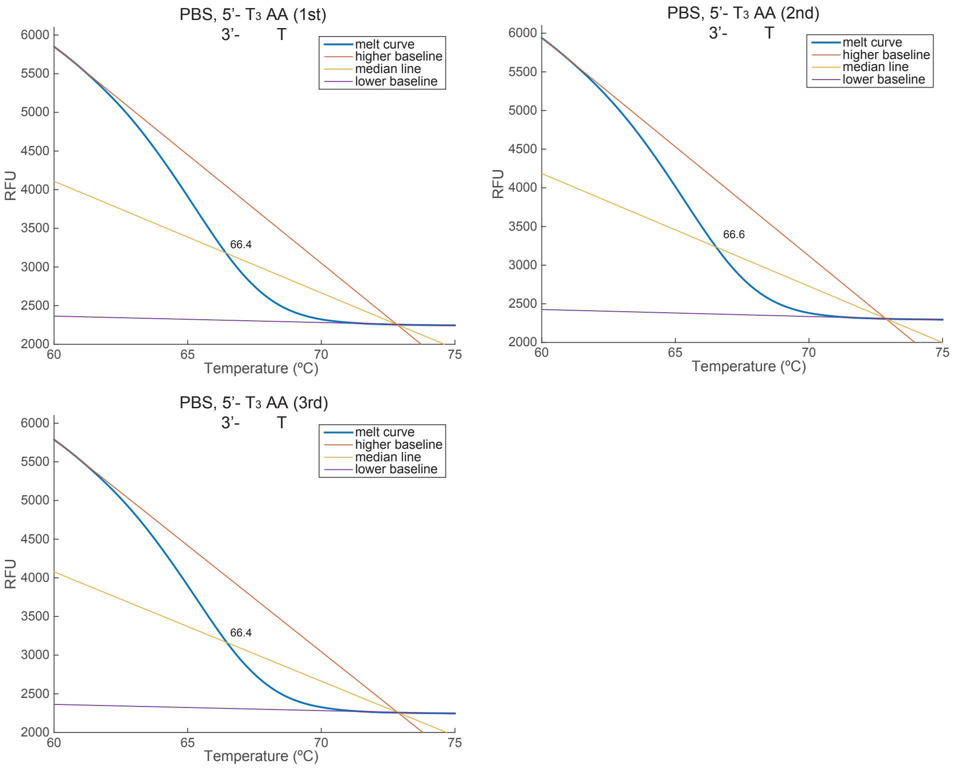

Supplementary Figure 80: Melt curves of 5′ 4nt T_3A dangle adjacent to closing base A in 1x PBS buffer.

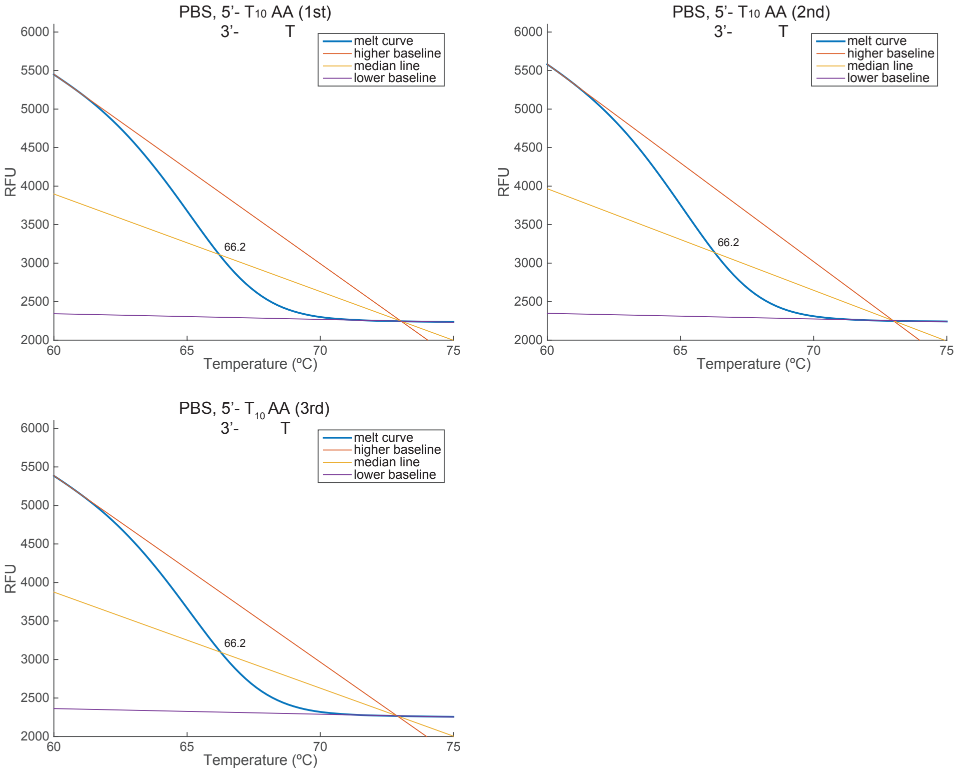

Supplementary Figure 81: Melt curves of 5′ 11nt $T_{10}A$ dangle adjacent to closing base A in 1x PBS buffer.

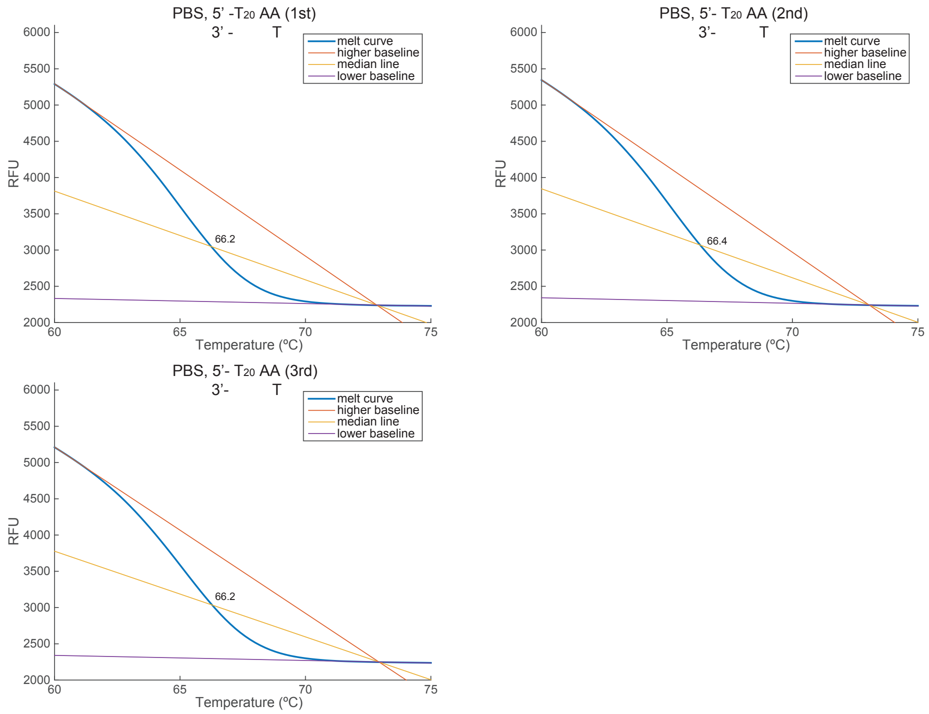

Supplementary Figure 82: Melt curves of 5′ 21nt T_{20}A dangle adjacent to closing base A in 1x PBS buffer.

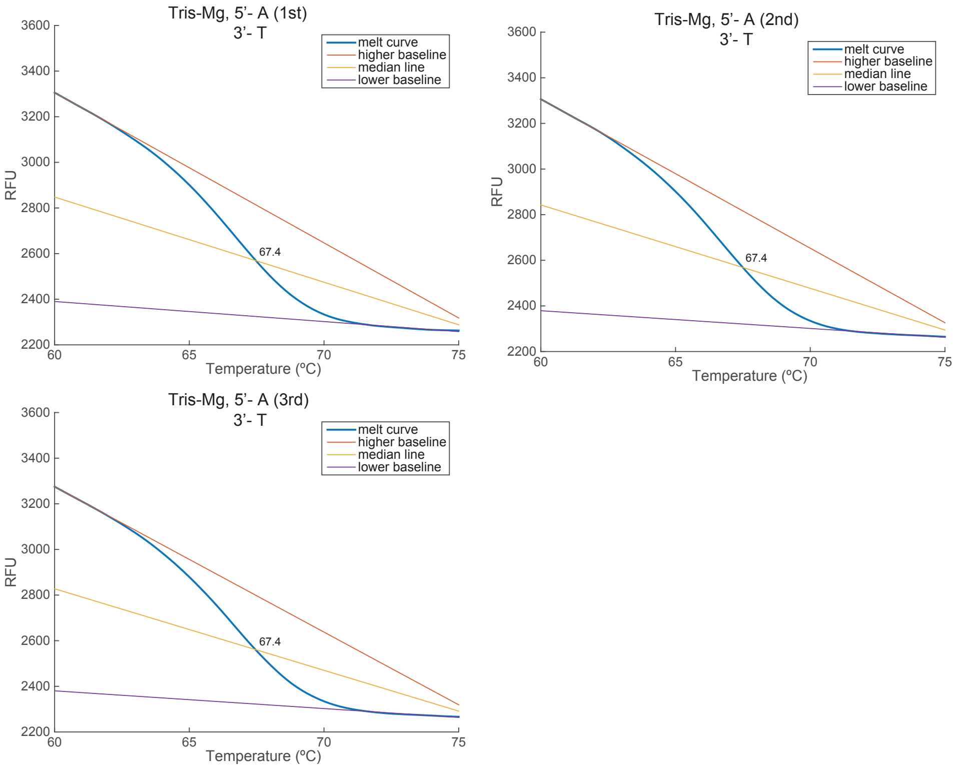

Supplementary Figure 83: Melt curves of the reference duplex with an A/T closing base pair and no dangle, in Tris-$MgCl_2$ buffer.

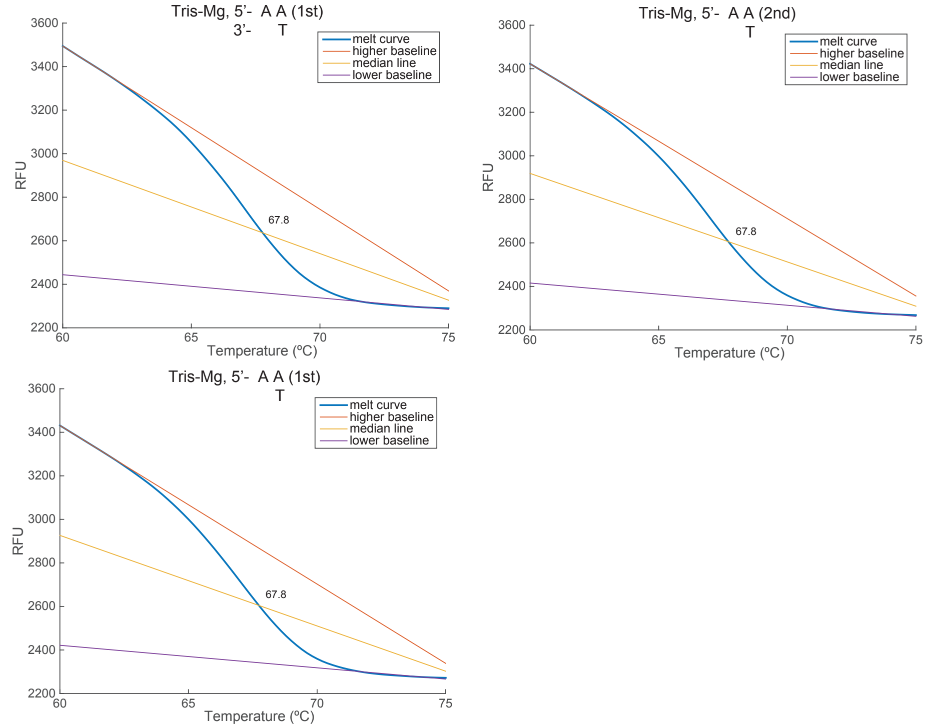

Supplementary Figure 84: Melt curves of 5′ 1nt A dangle adjacent to closing base adenine in Tris-$MgCl_2$ buffer.

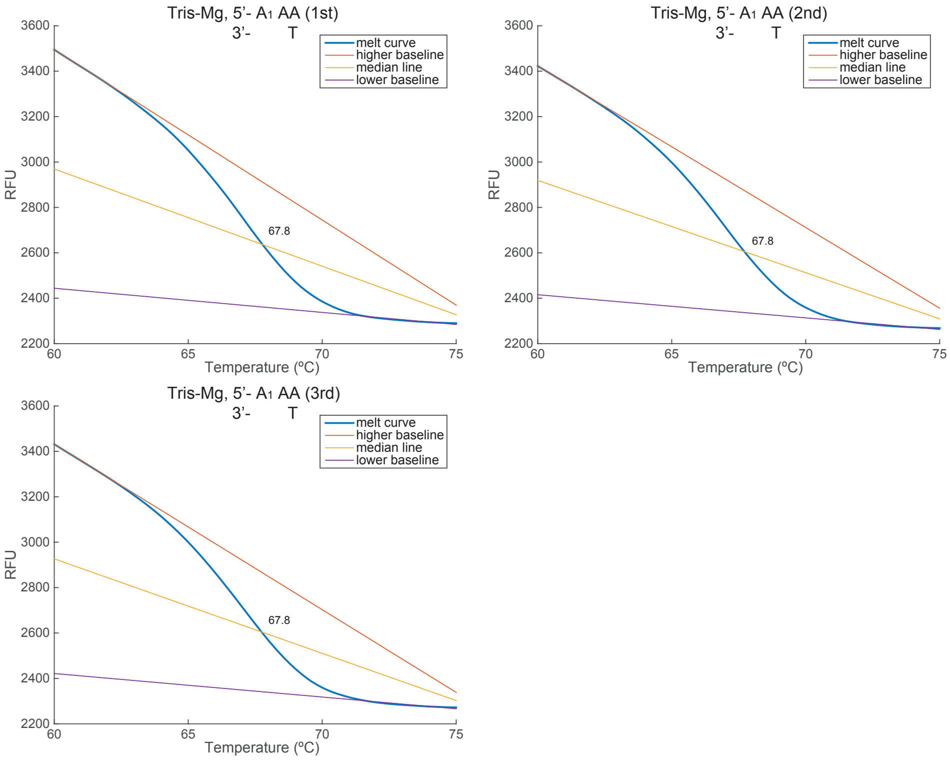

Supplementary Figure 85: Melt curves of 5′ 2nt AA dangle adjacent to closing base adenine in Tris-$MgCl_2$ buffer.

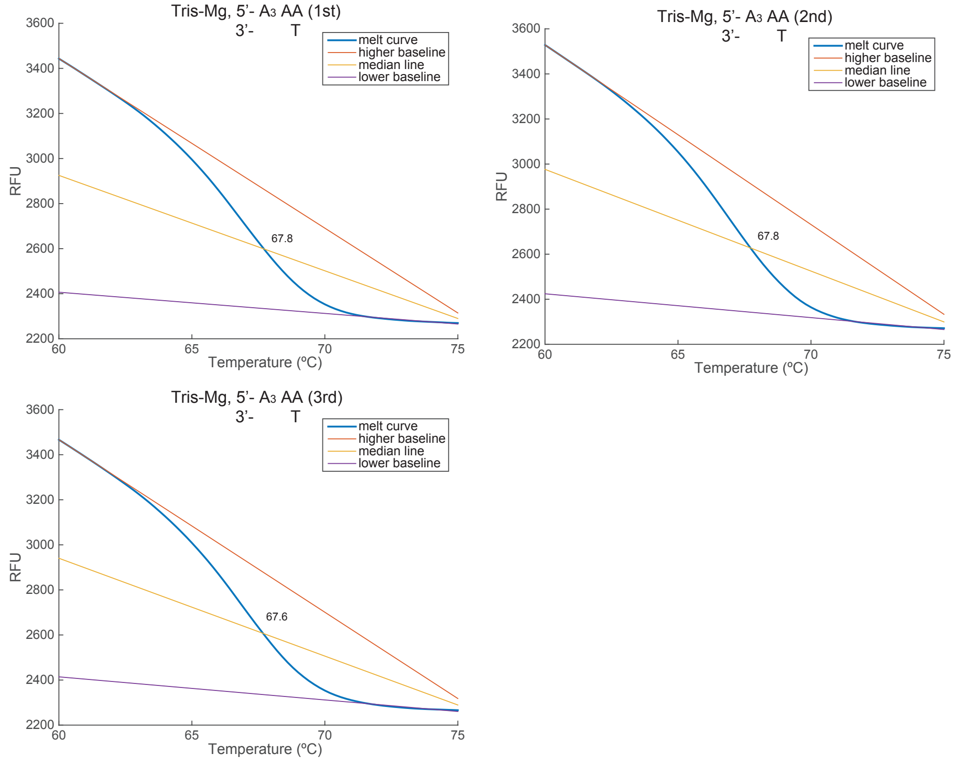

Supplementary Figure 86: Melt curves of 5′ 4nt A_4 dangle adjacent to closing base adenine in Tris-$MgCl_2$ buffer.

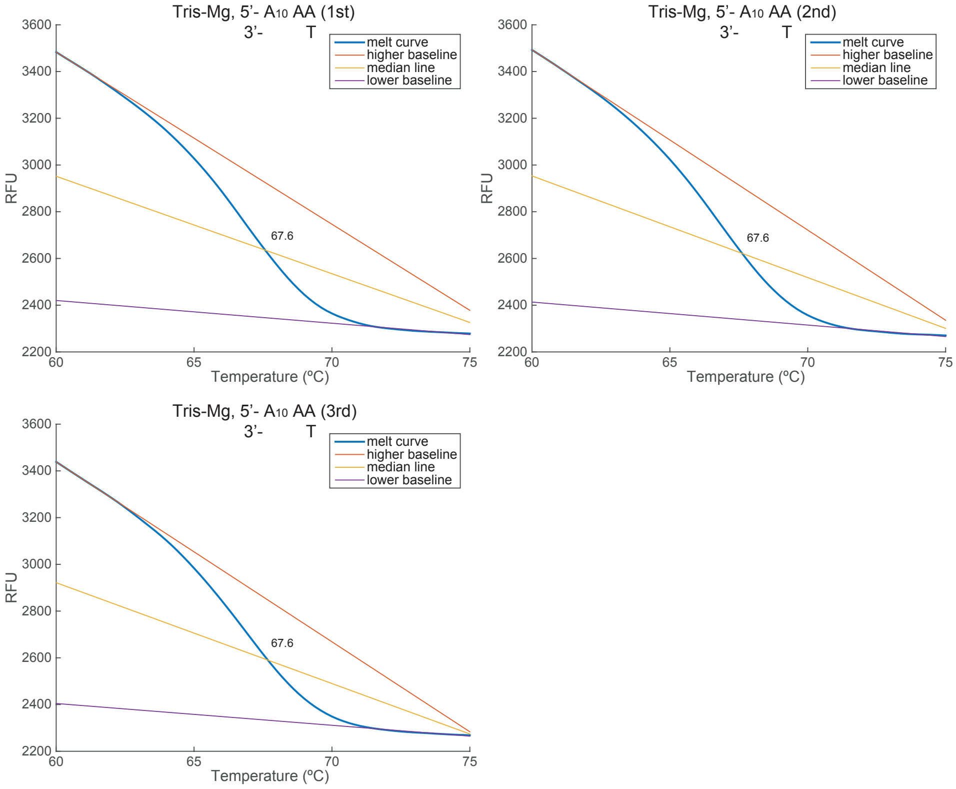

Supplementary Figure 87: Melt curves of 5′ 10nt A_{10} dangle adjacent to closing base adenine in Tris-$MgCl_2$ buffer.

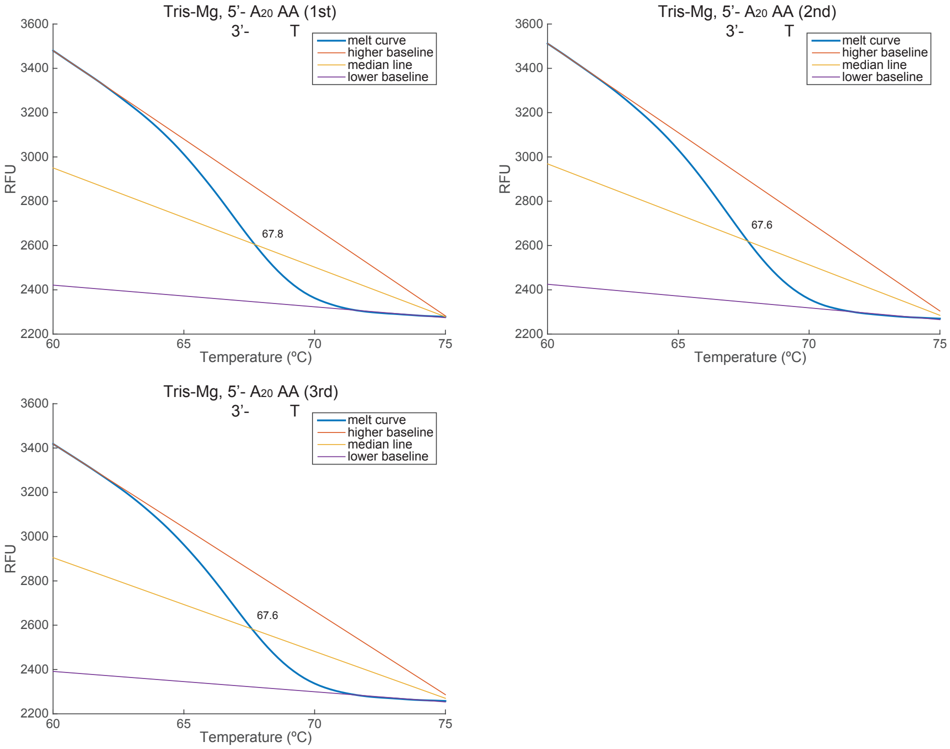

Supplementary Figure 88: Melt curves of 5′ 20nt A_{20} dangle adjacent to closing base adenine in Tris-$MgCl_2$ buffer.

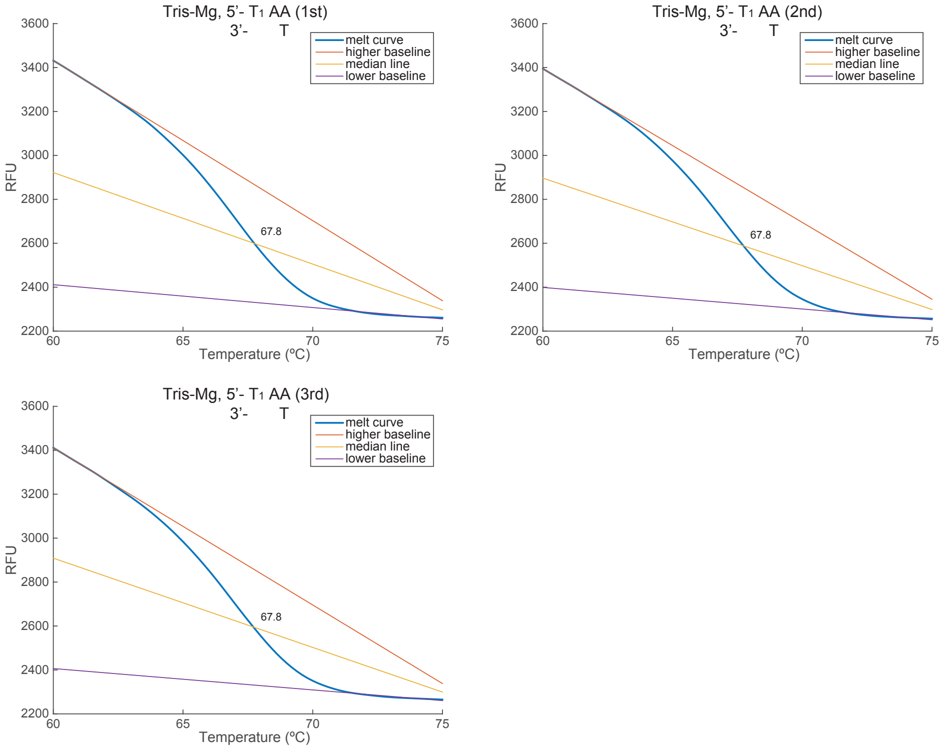

Supplementary Figure 89: Melt curves of 5′ TA dangle adjacent to closing base adenine in Tris-$MgCl_2$ buffer..

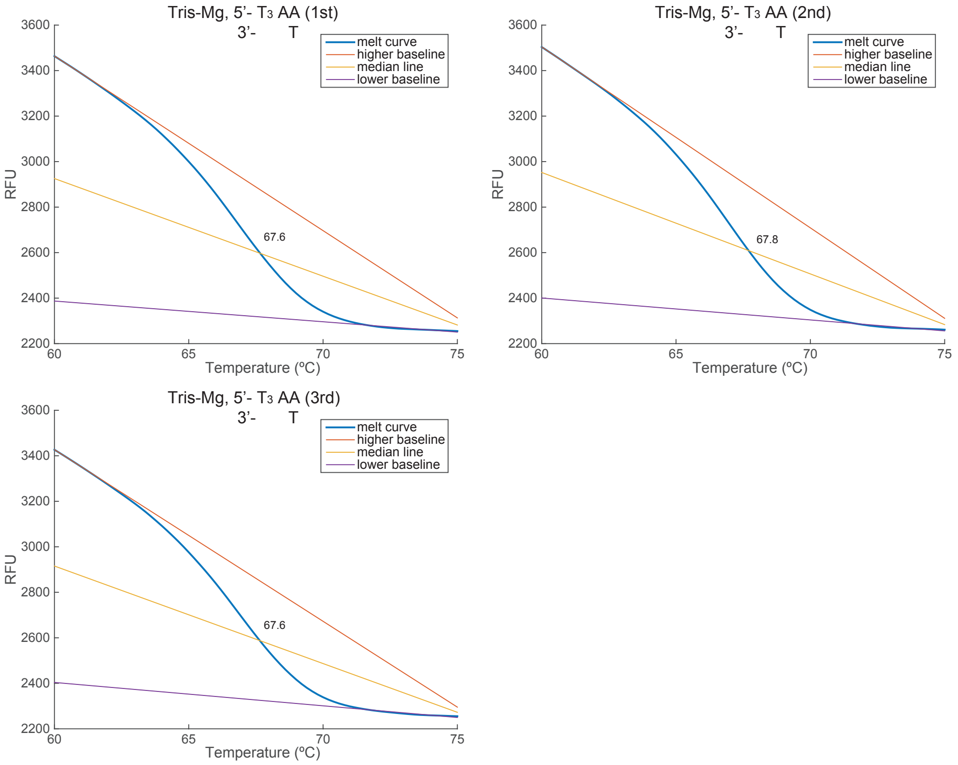

Supplementary Figure 90: Melt curves of 5′ 4nt T_3A dangle adjacent to closing base adenine in Tris-$MgCl_2$ buffer.

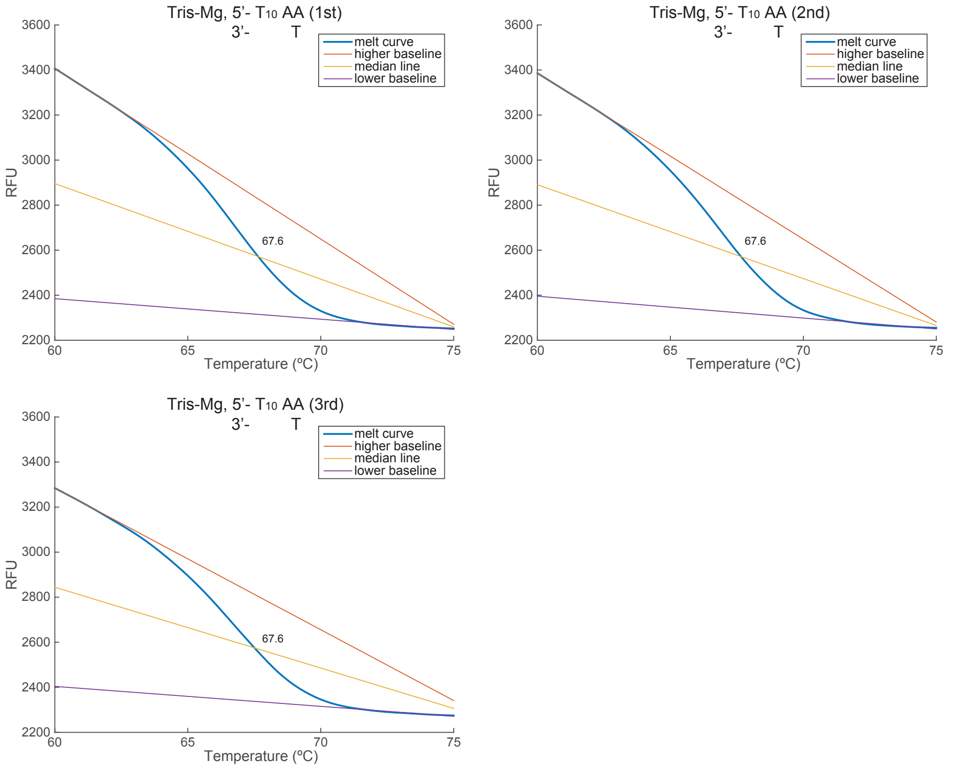

Supplementary Figure 91: Melt curves of 5′ 11nt T_{10}A dangle adjacent to closing base adenine in Tris-$MgCl_2$ buffer.

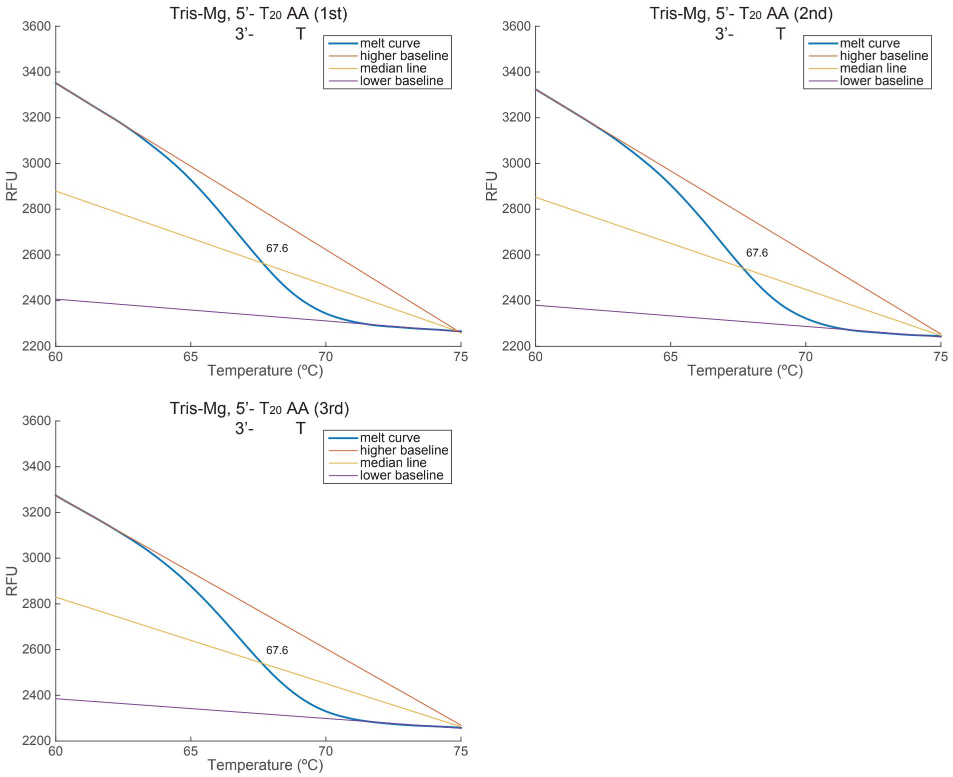

Supplementary Figure 92: Melt curves of 5′ 21nt T_{20}A dangle adjacent to closing base adenine in Tris-$MgCl_2$ buffer.

Oligo Name	ϵ	A_{260}	Conc. (μM)	Oligo Name	ϵ	A_{260}	Conc. (μM)
5p-Comp-A	308700	29.167	94.48	3p-Comp-A	308100	25.278	82.04
5p-Comp-T	315300	35.190	111.61	3p-Comp-T	314100	28.007	89.17
5p-Comp-C	310900	33.015	106.19	3p-Comp-C	311300	23.668	76.03
5p-Comp-G	308100	29.30	95.11	3p-Comp-G	306500	30.055	98.06

Supplementary Table 1: Concentrations inferred via absorbance at 260 nm and literature-based extinction coefficients.

Oligo Name	ratio	Oligos Name	ratio	Oligo Name	ratio	Oligos name	ratio
5p-Ref-A/comp-A	2.304	5p-Ref-T/comp-AT	2.230	5p-Ref-C/comp-C	2.034	5p-Ref-G/comp-G	2.008
5p-Ref-AA/comp-A	2.266	5p-Ref-AT/comp-T	2.118	5p-Ref-AC/comp-C	2.166	5p-Ref-AG/comp-G	2.104
5p-Ref-TA/comp-A	2.240	5p-Ref-TT/comp-T	2.266	5p-Ref-TC/comp-C	2.154	5p-Ref-TG/comp-G	2.050
5p-Ref-CA/comp-A	2.186	5p-Ref-CT/comp-T	2.304	5p-Ref-CC/comp-C	2.030	5p-Ref-CG/comp-G	1.858
5p-Ref-GA/comp-A	2.192	5p-Ref-GT/comp-T	2.130	5p-Ref-GC/comp-C	1.990	5p-Ref-GG/comp-G	1.934

Supplementary Table 2: Stoichiometry between reference strand and complementary strand determined by PAGE.

Oligo Name	ratio	Oligos Name	ratio	Oligo Name	ratio	Oligos name	ratio
3p-Ref-A/comp-A	1.700	3p-Ref-T/comp-AT	1.896	3p-Ref-C/comp-C	1.662	3p-Ref-G/comp-G	2.080
3p-Ref-AA/comp-A	1.614	3p-Ref-AT/comp-T	2.214	3p-Ref-AC/comp-C	2.054	3p-Ref-AG/comp-G	2.160
3p-Ref-TA/comp-A	1.672	3p-Ref-TT/comp-T	2.312	3p-Ref-TC/comp-C	2.068	3p-Ref-TG/comp-G	2.246
3p-Ref-CA/comp-A	1.964	3p-Ref-CT/comp-T	2.42	3p-Ref-CC/comp-C	1.758	3p-Ref-CG/comp-G	2.184
3p-Ref-GA/comp-A	1.748	3p-Ref-GT/comp-T	2.350	3p-Ref-GC/comp-C	1.870	3p-Ref-GG/comp-G	2.24

Supplementary Table 3: Stoichiometry between reference strand and complementary strand determined by PAGE.

	ΔG°_{10} (kcal/mol)	ΔG°_{25} (kcal/mol)	ΔG°_{37} (kcal/mol)	ΔG°_{45} (kcal/mol)	$\Delta G^\circ_{\mathrm{Mg},25}$ (kcal/mol)
5′-ROX-A	-0.296 ±0.061	-0.216 ±0.038	-0.276 ±0.043	-0.182 ±0.046	-0.290 ±0.056
5′-ROX-T	-0.057 ±0.034	-0.082 ±0.026	-0.201 ±0.057	-0.127 ±0.037	-0.076 ±0.037
5′-ROX-C	-0.289 ±0.033	-0.407 ±0.020	-0.367 ±0.049	-0.472 ±0.026	-0.249 ±0.059
5′-ROX-G	-0.278 ±0.065	-0.409 ±0.039	-0.515 ±0.042	-0.491 ±0.035	-0.308 ±0.033
5′-A532-A	-0.491 ±0.015	-0.303 ±0.031	-0.343 ±0.049	-0.388 ±0.027	-0.417 ±0.029
5′-A532-T	-0.438 ±0.030	-0.392 ±0.019	-0.356 ±0.037	-0.454 ±0.036	-0.466 ±0.035
5′-A532-C	-0.587 ±0.030	-0.498 ±0.039	-0.428 ±0.040	-0.521 ±0.033	-0.623 ±0.072
5′-A532-G	-0.350 ±0.027	-0.533 ±0.028	-0.404 ±0.024	-0.555 ±0.033	-0.389 ±0.038
A-ROX-3′	-0.601 ±0.044	-0.654 ±0.028	-0.450 ±0.052	-0.529 ±0.038	-0.438 ±0.038
T-ROX-3′	-1.289 ±0.079	-0.842 ±0.080	-1.327 ±0.141	-0.800 ±0.056	-0.737 ±0.069
C-ROX-3′	-1.035 ±0.104	-0.900 ±0.069	-0.889 ±0.074	-0.996 ±0.061	-0.946 ±0.075
G-ROX-3′	-0.640 ±0.053	-0.717 ±0.057	-0.692 ±0.066	-0.788 ±0.074	-0.349 ±0.036
A-A532-3′	-0.377 ±0.041	-0.135 ±0.048	-0.084 ±0.058	-0.134 ±0.048	-0.531 ±0.106
T-A532-3′	-0.321 ±0.031	-0.267 ±0.019	-0.225 ±0.037	-0.321 ±0.037	-0.345 ±0.035
C-A532-3′	-0.211 ±0.047	-0.094 ±0.027	-0.248 ±0.051	-0.039 ±0.046	-0.091 ±0.032
G-A532-3′	-0.256 ±0.026	-0.304 ±0.059	-0.205 ±0.054	-0.265 ±0.021	-0.273 ±0.033

Supplementary Table 4: Summary of ΔG° values of fluorophores at 5′ and 3′ of given strands, detailed strand were listed in section 12.

	ΔG°_{37} (kcal/mol)	ΔH° (kcal/mol)	ΔS° (cal/mol·K)
5′-ROX-A	-0.276 ±0.043	-0.81 ±0.84	-1.89 ±2.78
5′-ROX-T	-0.201 ±0.057	0.67 ±0.57	2.55 ±1.87
5′-ROX-C	-0.367 ±0.049	0.95 ±0.49	4.47 ±1.61
5′-ROX-G	-0.515 ±0.042	1.33 ±0.76	5.80 ±2.50
5′-A532-A	-0.343 ±0.049	-1.54 ±0.30	-3.75 ±0.99
5′-A532-T	-0.356 ±0.037	-0.51 ±0.51	-0.35 ±1.67
5′-A532-C	-0.428 ±0.040	-1.32 ±0.51	-2.67 ±1.68
5′-A532-G	-0.404 ±0.024	0.64 ±0.46	3.60 ±1.51
A-ROX-3′	-0.450 ±0.052	-1.72 ±0.67	-3.76 ±2.20
T-ROX-3′	-1.327 ±0.141	-4.44 ±1.13	-11.40 ±3.74
C-ROX-3′	-0.889 ±0.074	-0.93 ±1.28	0.05 ±4.22
G-ROX-3′	-0.692 ±0.066	0.31 ±0.95	3.35 ±3.13
A-A532-3′	-0.084 ±0.058	-2.47 ±0.71	-7.53 ±2.34
T-A532-3′	-0.225 ±0.037	-0.53 ±0.52	-0.84 ±1.70
C-A532-3′	-0.248 ±0.051	-0.89 ±0.73	-2.55 ±2.42
G-A532-3′	-0.205 ±0.054	-0.23 ±0.41	0.10 ±1.34

Supplementary Table 5: Summary of ΔG° at 37°C, and fitted ΔH° and ΔS° values of fluorophores at 5′ and 3′ of given strands.

	ΔG°_{10} (kcal/mol)	ΔG°_{25} (kcal/mol)	ΔG°_{37} (kcal/mol)	ΔG°_{45} (kcal/mol)	$\Delta G^{\circ}_{\mathrm{Mg},25}$ (kcal/mol)
AA<br>T	-0.671 ±0.049 (9.2)	-0.549 ±0.045 (9.0)	-0.647 ±0.084 (7.1)	-0.271 ±0.111 (-11.0)	-0.595 ±0.080 (8.0)
AT<br>A	-0.512 ±0.059 (5.7)	-0.483 ±0.038 (8.5)	-0.368 ±0.071 (4.7)	-0.213 ±0.094 (1.0)	-0.511 ±0.045 (8.9)
AC<br>G	-0.957 ±0.047 (9.6)	-0.835 ±0.042 (8.4)	-0.635 ±0.046 (9.6)	-0.629 ±0.050 (7.9)	-0.777 ±0.097 (5.0)
AG<br>C	-0.351 ±0.115 (5.8)	-0.678 ±0.107 (0.7)	-0.632 ±0.059 (7.0)	-0.505 ±0.120 (-15.1)	-0.604 ±0.112 (2.9)
TA<br>T	-0.668 ±0.182 (-37.1)	-0.566 ±0.111 (-2.5)	-0.432 ±0.086 (8.2)	-0.330 ±0.074 (8.8)	-0.504 ±0.072 (9.4)
TT<br>A	-0.281 ±0.045 (9.2)	-0.047 ±0.056 (8.5)	-0.155 ±0.056 (9.5)	-0.006 ±0.108 (3.1)	-0.145 ±0.040 (10.0)
TC<br>G	-0.548 ±0.032 (10.0)	-0.427 ±0.076 (4.2)	-0.364 ±0.064 (8.9)	-0.268 ±0.094 (4.7)	-0.462 ±0.052 (10.0)
TG<br>C	-0.321 ±0.103 (3.9)	-0.513 ±0.051 (8.5)	-0.548 ±0.038 (8.0)	-0.505 ±0.042 (7.2)	-0.405 ±0.067 (1.8)
CA<br>T	-0.423 ±0.070 (8.1)	-0.279 ±0.108 (2.6)	-0.224 ±0.123 (2.5)	-0.064 ±0.124 (-6.0)	-0.297 ±0.034 (10.0)
CT<br>A	-0.169 ±0.035 (10.0)	-0.042 ±0.050 (9.8)	0.001 ±0.041 (9.7)	-0.006 ±0.054 (9.4)	-0.105 ±0.033 (9.9)
CC<br>G	-0.469 ±0.042 (9.8)	-0.519 ±0.038 (10.0)	-0.322 ±0.094 (5.6)	-0.615 ±0.092 (4.4)	-0.443 ±0.068 (10.0)
CG<br>C	-0.580 ±0.066 (7.0)	-0.603 ±0.097 (6.6)	-0.420 ±0.065 (9.8)	-0.552 ±0.096 (7.6)	-0.467 ±0.033 (9.4)
GA<br>T	-0.414 ±0.079 (10.0)	-0.389 ±0.042 (9.9)	-0.355 ±0.062 (6.4)	-0.381 ±0.058 (6.3)	-0.468 ±0.058 (9.6)
GT<br>A	-0.226 ±0.099 (-5.8)	-0.121 ±0.045 (8.9)	-0.154 ±0.052 (9.0)	-0.186 ±0.048 (9.6)	-0.148 ±0.084 (5.6)
GC<br>G	-0.712 ±0.069 (10.0)	-0.576 ±0.049 (8.0)	-0.422 ±0.056 (9.5)	-0.506 ±0.052 (4.2)	-0.601 ±0.093 (6.7)
GG<br>C	-0.513 ±0.133 (2.4)	-0.703 ±0.033 (9.7)	-0.520 ±0.088 (-7.9)	-0.579 ±0.126 (8.6)	-0.520 ±0.074 (-2.5)
AA<br>T	-0.072 ±0.102 (7.4)	-0.144 ±0.045 (9.9)	-0.011 ±0.070 (9.4)	-0.209 ±0.158 (-0.1)	-0.328 ±0.127 (6.2)
TA<br>A	-0.398 ±0.095 (5.2)	-0.324 ±0.077 (9.1)	-0.195 ±0.156 (4.4)	-0.382 ±0.087 (5.5)	-0.267 ±0.113 (7.7)
CA<br>G	-0.857 ±0.170 (6.4)	-0.588 ±0.063 (9.8)	-0.659 ±0.091 (7.0)	-0.550 ±0.059 (8.1)	-0.622 ±0.097 (5.7)
GA<br>C	0.106 ±0.045 (9.9)	-0.131 ±0.058 (9.3)	-0.118 ±0.049 (10.0)	-0.151 ±0.063 (8.6)	0.036 ±0.039 (9.9)
AT<br>T	-0.037 ±0.061 (7.7)	0.139 ±0.135 (5.9)	0.183 ±0.046 (10.0)	0.153 ±0.089 (1.6)	0.008 ±0.237 (-7.1)
TT<br>A	0.166 ±0.102 (7.2)	0.091 ±0.072 (9.0)	0.223 ±0.168 (6.0)	-0.062 ±0.146 (5.4)	0.024 ±0.146 (-2.0)
CT<br>G	-0.344 ±0.118 (5.3)	-0.261 ±0.089 (4.1)	0.003 ±0.115 (8.0)	-0.104 ±0.067 (7.3)	-0.239 ±0.117 (6.5)
GT<br>C	0.642 ±0.067 (7.6)	0.398 ±0.062 (9.2)	0.264 ±0.077 (8.0)	0.165 ±0.065 (9.6)	0.541 ±0.047 (9.3)
AC<br>T	0.329 ±0.057 (9.1)	0.310 ±0.101 (7.7)	0.485 ±0.060 (10.0)	0.303 ±0.051 (9.3)	0.117 ±0.060 (9.9)
TC<br>A	-0.022 ±0.082 (9.2)	0.036 ±0.092 (10.0)	0.107 ±0.167 (3.8)	0.087 ±0.127 (9.6)	-0.069 ±0.068 (8.0)
CC<br>G	-0.356 ±0.177 (1.2)	-0.155 ±0.073 (9.4)	-0.081 ±0.102 (8.5)	-0.274 ±0.075 (9.0)	-0.100 ±0.219 (4.7)
GC<br>C	0.122 ±0.114 (-8.7)	0.025 ±0.069 (7.8)	0.034 ±0.090 (9.2)	0.048 ±0.066 (9.5)	0.161 ±0.041 (9.9)
AG<br>T	0.295 ±0.236 (2.4)	0.093 ±0.036 (10.0)	0.203 ±0.063 (9.6)	-0.142 ±0.145 (0.2)	0.095 ±0.164 (-3.3)
TG<br>A	-0.091 ±0.077 (9.5)	-0.278 ±0.125 (0.5)	-0.066 ±0.121 (8.0)	-0.154 ±0.153 (0.3)	-0.186 ±0.075 (7.9)
CG<br>G	-0.662 ±0.137 (2.3)	-0.625 ±0.046 (9.9)	-0.544 ±0.142 (-3.0)	-0.610 ±0.112 (8.8)	-0.503 ±0.125 (8.2)
GG<br>C	0.292 ±0.037 (9.9)	0.046 ±0.056 (10.0)	0.067 ±0.077 (8.0)	-0.001 ±0.070 (7.6)	0.262 ±0.038 (7.9)

Supplementary Table 6: Consensus ΔG° values for single-base dangle parameters. Shown in parentheses are Confidence Scores.

	ΔG°_{37} (kcal/mol)	ΔH° (kcal/mol)	ΔS° (cal/mol·K)
AA<br>T	-0.647 ±0.084 (7.1)	-3.30 ±1.28 (2.1)	-9.15 ±4.36 (1.3)
AT<br>A	-0.368 ±0.071 (4.7)	-2.77 ±0.96 (6.2)	-7.84 ±3.17 (6.1)
AC<br>G	-0.635 ±0.046 (9.6)	-3.78 ±0.87 (8.0)	-9.96 ±2.86 (8.0)
AG<br>C	-0.632 ±0.059 (7.0)	-0.41 ±1.37 (4.6)	0.46 ±4.62 (4.4)
TA<br>T	-0.432 ±0.086 (8.2)	-2.95 ±1.28 (4.0)	-8.04 ±3.97 (5.0)
TT<br>A	-0.155 ±0.056 (9.5)	-2.25 ±0.74 (9.5)	-7.06 ±2.46 (9.5)
TC<br>G	-0.364 ±0.064 (8.9)	-2.71 ±0.85 (7.9)	-7.67 ±2.83 (7.8)
TG<br>C	-0.548 ±0.038 (8.0)	0.75 ±0.92 (6.7)	4.06 ±3.06 (6.6)
CA<br>T	-0.224 ±0.123 (2.5)	-3.43 ±1.57 (3.1)	-10.53 ±5.14 (3.3)
CT<br>A	0.001 ±0.041 (9.7)	-1.71 ±0.53 (10.0)	-5.48 ±1.75 (10.0)
CC<br>G	-0.322 ±0.094 (5.6)	0.10 ±1.26 (4.8)	1.98 ±4.29 (4.3)
CG<br>C	-0.420 ±0.065 (9.8)	-1.04 ±0.77 (10.0)	-1.60 ±2.55 (10.0)
GA<br>T	-0.355 ±0.062 (6.4)	-0.68 ±0.90 (9.0)	-0.93 ±2.96 (9.0)
GT<br>A	-0.154 ±0.052 (9.0)	-0.61 ±1.11 (4.2)	-1.51 ±3.66 (4.2)
GC<br>G	-0.422 ±0.056 (9.5)	-2.06 ±0.62 (10.0)	-4.96 ±2.05 (9.9)
GG<br>C	-0.520 ±0.088 (-7.9)	-1.07 ±0.92 (9.9)	-1.67 ±2.98 (9.9)
AA<br>T	-0.011 ±0.070 (9.4)	0.40 ±0.95 (9.7)	1.75 ±3.16 (9.6)
TA<br>A	-0.195 ±0.156 (4.4)	-0.95 ±1.16 (8.1)	-2.04 ±3.79 (8.2)
CA<br>G	-0.659 ±0.091 (7.0)	-2.64 ±1.76 (5.3)	-6.53 ±5.68 (5.6)
GA<br>C	-0.118 ±0.049 (10.0)	2.15 ±0.77 (9.7)	7.28 ±2.56 (9.7)
AT<br>T	0.183 ±0.046 (10.0)	-1.64 ±0.70 (9.8)	-5.75 ±2.33 (9.7)
TT<br>A	0.223 ±0.168 (6.0)	0.45 ±1.18 (9.7)	1.05 ±3.87 (9.7)
CT<br>G	0.003 ±0.115 (8.0)	-2.51 ±1.67 (4.7)	-7.67 ±5.41 (5.0)
GT<br>C	0.264 ±0.077 (8.0)	4.56 ±0.87 (9.5)	13.83 ±2.86 (9.5)
AC<br>T	0.485 ±0.060 (10.0)	0.14 ±0.72 (10.0)	-0.66 ±2.37 (10.0)
TC<br>A	0.107 ±0.167 (3.8)	-0.92 ±1.27 (10.0)	-3.22 ±4.20 (10.0)
CC<br>G	-0.081 ±0.102 (8.5)	-1.14 ±1.89 (6.3)	-3.04 ±6.18 (6.4)
GC<br>C	0.034 ±0.090 (9.2)	0.84 ±1.27 (4.7)	2.54 ±4.15 (4.8)
AG<br>T	0.203 ±0.063 (9.6)	2.33 ±1.24 (9.4)	7.45 ±4.16 (9.3)
TG<br>A	-0.066 ±0.121 (8.0)	0.27 ±1.52 (7.8)	1.49 ±5.17 (7.6)
CG<br>G	-0.544 ±0.142 (-3.0)	-1.08 ±1.19 (8.7)	-1.54 ±3.85 (8.8)
GG<br>C	0.067 ±0.077 (8.0)	2.62 ±0.68 (9.8)	8.30 ±2.25 (9.8)

Supplementary Table 7: Consensus ΔG°, ΔH°, and ΔS° summary for single-base dangle in 1x PBS at 37°C. Shown in parentheses are Confidence Scores.

	ΔG°_{10} (kcal/mol)	ΔG°_{25} (kcal/mol)	ΔG°_{37} (kcal/mol)	ΔG°_{45} (kcal/mol)	$\Delta G^\circ_{\mathrm{Mg},25}$ (kcal/mol)
(ROX) $^{AA}_{\ \mathrm{T}}$	-0.622 ±0.079	-0.587 ±0.053	-0.534 ±0.094	-0.112 ±0.049	-0.487 ±0.108
(A532) $^{AA}_{\ \mathrm{T}}$	-0.687 ±0.025	-0.514 ±0.050	-0.710 ±0.052	-0.412 ±0.044	-0.628 ±0.034
(ROX) $^{AT}_{\ \mathrm{A}}$	-0.594 ±0.056	-0.445 ±0.043	-0.467 ±0.061	-0.090 ±0.058	-0.466 ±0.062
(A532) $^{AT}_{\ \mathrm{A}}$	-0.459 ±0.036	-0.516 ±0.038	-0.299 ±0.042	-0.330 ±0.055	-0.539 ±0.037
(ROX) $^{AC}_{\ \mathrm{G}}$	-0.934 ±0.055	-0.805 ±0.033	-0.662 ±0.058	-0.659 ±0.029	-0.671 ±0.067
(A532) $^{AC}_{\ \mathrm{G}}$	-0.985 ±0.066	-0.883 ±0.053	-0.607 ±0.062	-0.563 ±0.064	-0.902 ±0.079
(ROX) $^{AG}_{\ \mathrm{C}}$	-0.484 ±0.092	-0.539 ±0.064	-0.703 ±0.058	-0.363 ±0.040	-0.671 ±0.036
(A532) $^{AG}_{\ \mathrm{C}}$	-0.216 ±0.094	-0.813 ±0.063	-0.574 ±0.048	-0.687 ±0.052	-0.417 ±0.099
(ROX) $^{TA}_{\ \mathrm{T}}$	-0.348 ±0.066	-0.422 ±0.058	-0.337 ±0.102	-0.275 ±0.069	-0.536 ±0.057
(A532) $^{TA}_{\ \mathrm{T}}$	-0.826 ±0.033	-0.712 ±0.059	-0.501 ±0.074	-0.404 ±0.094	-0.442 ±0.111
(ROX) $^{TT}_{\ \mathrm{A}}$	-0.254 ±0.042	-0.095 ±0.056	-0.189 ±0.067	0.075 ±0.044	-0.155 ±0.067
(A532) $^{TT}_{\ \mathrm{A}}$	-0.321 ±0.062	0.008 ±0.063	-0.116 ±0.075	-0.179 ±0.093	-0.139 ±0.044
(ROX) $^{TC}_{\ \mathrm{G}}$	-0.551 ±0.040	-0.532 ±0.062	-0.308 ±0.074	-0.173 ±0.059	-0.451 ±0.072
(A532) $^{TC}_{\ \mathrm{G}}$	-0.544 ±0.050	-0.348 ±0.046	-0.416 ±0.070	-0.398 ±0.079	-0.473 ±0.074
(ROX) $^{TG}_{\ \mathrm{C}}$	-0.438 ±0.067	-0.479 ±0.040	-0.504 ±0.044	-0.454 ±0.043	-0.478 ±0.036
(A532) $^{TG}_{\ \mathrm{C}}$	-0.186 ±0.077	-0.570 ±0.067	-0.579 ±0.030	-0.543 ±0.033	-0.308 ±0.048
(ROX) $^{CA}_{\ \mathrm{T}}$	-0.496 ±0.075	-0.423 ±0.075	-0.380 ±0.081	0.072 ±0.048	-0.300 ±0.060
(A532) $^{CA}_{\ \mathrm{T}}$	-0.358 ±0.067	-0.153 ±0.065	-0.070 ±0.079	-0.254 ±0.067	-0.295 ±0.030
(ROX) $^{CT}_{\ \mathrm{A}}$	-0.177 ±0.057	-0.074 ±0.092	-0.026 ±0.065	0.014 ±0.037	-0.092 ±0.047
(A532) $^{CT}_{\ \mathrm{A}}$	-0.163 ±0.040	-0.034 ±0.023	0.017 ±0.039	-0.052 ±0.088	-0.117 ±0.044
(ROX) $^{CC}_{\ \mathrm{G}}$	-0.456 ±0.040	-0.517 ±0.027	-0.456 ±0.090	-0.713 ±0.059	-0.457 ±0.103
(A532) $^{CC}_{\ \mathrm{G}}$	-0.491 ±0.069	-0.524 ±0.070	-0.243 ±0.054	-0.492 ±0.073	-0.432 ±0.087
(ROX) $^{CG}_{\ \mathrm{C}}$	-0.485 ±0.078	-0.487 ±0.088	-0.449 ±0.099	-0.421 ±0.120	-0.444 ±0.040
(A532) $^{CG}_{\ \mathrm{C}}$	-0.618 ±0.031	-0.706 ±0.079	-0.398 ±0.076	-0.606 ±0.050	-0.490 ±0.042
(ROX) $^{GA}_{\ \mathrm{T}}$	-0.432 ±0.146	-0.408 ±0.074	-0.412 ±0.043	-0.298 ±0.061	-0.497 ±0.066
(A532) $^{GA}_{\ \mathrm{T}}$	-0.407 ±0.060	-0.380 ±0.034	-0.274 ±0.061	-0.423 ±0.031	-0.432 ±0.083
(ROX) $^{GT}_{\ \mathrm{A}}$	-0.344 ±0.042	-0.092 ±0.039	-0.196 ±0.059	-0.154 ±0.077	-0.077 ±0.048
(A532) $^{GT}_{\ \mathrm{A}}$	-0.081 ±0.052	-0.166 ±0.061	-0.110 ±0.062	-0.204 ±0.044	-0.269 ±0.081
(ROX) $^{GC}_{\ \mathrm{G}}$	-0.714 ±0.057	-0.616 ±0.041	-0.390 ±0.065	-0.571 ±0.038	-0.518 ±0.064
(A532) $^{GC}_{\ \mathrm{G}}$	-0.708 ±0.126	-0.520 ±0.055	-0.459 ±0.078	-0.443 ±0.037	-0.722 ±0.094
(ROX) $^{GG}_{\ \mathrm{C}}$	-0.729 ±0.111	-0.719 ±0.045	-0.664 ±0.048	-0.728 ±0.176	-0.611 ±0.037
(A532) $^{GG}_{\ \mathrm{C}}$	-0.416 ±0.050	-0.687 ±0.044	-0.436 ±0.028	-0.515 ±0.076	-0.417 ±0.041

Supplementary Table 8: Real ΔG° values for 5′ single-base dangles.

	ΔG°_{10} (kcal/mol)	ΔG°_{25} (kcal/mol)	ΔG°_{37} (kcal/mol)	ΔG°_{45} (kcal/mol)	$\Delta G^\circ_{\mathrm{Mg},25}$ (kcal/mol)
(ROX) $\genfrac{}{}{0pt}{}{AA}{\mathrm{T}}$	0.058 ±0.113	-0.154 ±0.050	0.033 ±0.083	-0.096 ±0.050	-0.230 ±0.071
(A532) $\genfrac{}{}{0pt}{}{AA}{\mathrm{T}}$	-0.155 ±0.073	-0.128 ±0.071	-0.060 ±0.091	-0.475 ±0.118	-0.509 ±0.132
(ROX) $\genfrac{}{}{0pt}{}{TA}{\mathrm{A}}$	-0.537 ±0.090	-0.255 ±0.100	-0.443 ±0.148	-0.266 ±0.078	-0.157 ±0.104
(A532) $\genfrac{}{}{0pt}{}{TA}{\mathrm{A}}$	-0.320 ±0.050	-0.378 ±0.078	-0.093 ±0.061	-0.470 ±0.058	-0.388 ±0.115
(ROX) $\genfrac{}{}{0pt}{}{CA}{\mathrm{G}}$	-0.607 ±0.188	-0.566 ±0.074	-0.546 ±0.093	-0.616 ±0.068	-0.756 ±0.091
(A532) $\genfrac{}{}{0pt}{}{CA}{\mathrm{G}}$	-0.963 ±0.079	-0.616 ±0.095	-0.743 ±0.069	-0.502 ±0.050	-0.534 ±0.060
(ROX) $\genfrac{}{}{0pt}{}{GA}{\mathrm{C}}$	0.121 ±0.082	-0.089 ±0.071	-0.117 ±0.075	-0.222 ±0.084	0.025 ±0.050
(A532) $\genfrac{}{}{0pt}{}{GA}{\mathrm{C}}$	0.100 ±0.032	-0.173 ±0.071	-0.118 ±0.064	-0.113 ±0.045	0.048 ±0.057
(ROX) $\genfrac{}{}{0pt}{}{AT}{\mathrm{T}}$	0.029 ±0.061	-0.043 ±0.127	0.191 ±0.067	0.261 ±0.053	0.149 ±0.048
(A532) $\genfrac{}{}{0pt}{}{AT}{\mathrm{T}}$	-0.097 ±0.055	0.265 ±0.088	0.176 ±0.061	0.035 ±0.058	-0.417 ±0.145
(ROX) $\genfrac{}{}{0pt}{}{TT}{\mathrm{A}}$	0.025 ±0.119	0.164 ±0.106	-0.042 ±0.187	0.115 ±0.117	0.223 ±0.081
(A532) $\genfrac{}{}{0pt}{}{TT}{\mathrm{A}}$	0.233 ±0.056	0.054 ±0.054	0.294 ±0.050	-0.229 ±0.110	-0.158 ±0.074
(ROX) $\genfrac{}{}{0pt}{}{CT}{\mathrm{G}}$	-0.169 ±0.114	-0.133 ±0.075	0.152 ±0.150	-0.186 ±0.071	-0.376 ±0.104
(A532) $\genfrac{}{}{0pt}{}{CT}{\mathrm{G}}$	-0.434 ±0.059	-0.346 ±0.049	-0.061 ±0.064	-0.045 ±0.050	-0.113 ±0.096
(ROX) $\genfrac{}{}{0pt}{}{GT}{\mathrm{C}}$	0.556 ±0.078	0.448 ±0.077	0.171 ±0.094	0.217 ±0.113	0.583 ±0.068
(A532) $\genfrac{}{}{0pt}{}{GT}{\mathrm{C}}$	0.693 ±0.046	0.354 ±0.069	0.320 ±0.057	0.148 ±0.036	0.516 ±0.042
(ROX) $\genfrac{}{}{0pt}{}{AC}{\mathrm{T}}$	0.380 ±0.077	0.236 ±0.070	0.488 ±0.084	0.330 ±0.047	0.126 ±0.039
(A532) $\genfrac{}{}{0pt}{}{AC}{\mathrm{T}}$	0.292 ±0.056	0.438 ±0.120	0.482 ±0.085	0.259 ±0.074	0.089 ±0.109
(ROX) $\genfrac{}{}{0pt}{}{TC}{\mathrm{A}}$	-0.103 ±0.127	0.057 ±0.172	-0.171 ±0.158	0.189 ±0.225	0.019 ±0.088
(A532) $\genfrac{}{}{0pt}{}{TC}{\mathrm{A}}$	0.012 ±0.054	0.028 ±0.063	0.188 ±0.046	0.062 ±0.054	-0.108 ±0.039
(ROX) $\genfrac{}{}{0pt}{}{CC}{\mathrm{G}}$	-0.074 ±0.135	-0.215 ±0.108	0.042 ±0.144	-0.347 ±0.102	-0.467 ±0.226
(A532) $\genfrac{}{}{0pt}{}{CC}{\mathrm{G}}$	-0.506 ±0.072	-0.117 ±0.069	-0.131 ±0.059	-0.226 ±0.066	-0.034 ±0.040
(ROX) $\genfrac{}{}{0pt}{}{GC}{\mathrm{C}}$	-0.059 ±0.059	0.097 ±0.068	-0.016 ±0.081	0.100 ±0.107	0.174 ±0.050
(A532) $\genfrac{}{}{0pt}{}{GC}{\mathrm{C}}$	0.240 ±0.038	-0.045 ±0.067	0.114 ±0.130	0.022 ±0.052	0.146 ±0.061
(ROX) $\genfrac{}{}{0pt}{}{AG}{\mathrm{T}}$	0.694 ±0.205	0.097 ±0.045	0.243 ±0.091	0.066 ±0.094	0.218 ±0.048
(A532) $\genfrac{}{}{0pt}{}{AG}{\mathrm{T}}$	0.167 ±0.066	0.088 ±0.054	0.172 ±0.070	-0.304 ±0.073	-0.186 ±0.109
(ROX) $\genfrac{}{}{0pt}{}{TG}{\mathrm{A}}$	-0.156 ±0.127	-0.091 ±0.086	-0.212 ±0.146	0.077 ±0.105	-0.096 ±0.089
(A532) $\genfrac{}{}{0pt}{}{TG}{\mathrm{A}}$	-0.065 ±0.050	-0.405 ±0.058	0.023 ±0.089	-0.309 ±0.070	-0.243 ±0.056
(ROX) $\genfrac{}{}{0pt}{}{CG}{\mathrm{G}}$	-0.444 ±0.111	-0.643 ±0.076	-0.320 ±0.088	-0.670 ±0.078	-0.662 ±0.167
(A532) $\genfrac{}{}{0pt}{}{CG}{\mathrm{G}}$	-0.770 ±0.055	-0.613 ±0.049	-0.686 ±0.056	-0.487 ±0.159	-0.435 ±0.071
(ROX) $\genfrac{}{}{0pt}{}{GG}{\mathrm{C}}$	0.283 ±0.059	0.044 ±0.092	-0.024 ±0.092	0.092 ±0.085	0.303 ±0.040
(A532) $\genfrac{}{}{0pt}{}{GG}{\mathrm{C}}$	0.299 ±0.043	0.048 ±0.064	0.126 ±0.060	-0.047 ±0.041	0.226 ±0.036

Supplementary Table 9: Real ΔG° for $3'$ single-base dangles.

	ΔG°_{37} (kcal/mol)	ΔH° (kcal/mol)	ΔS° (cal/mol·K)
(ROX) $^{AA}_{\ \mathrm{T}}$	-0.534 ±0.094	-5.36 ±1.04	-16.26 ±3.42
(A532) $^{AA}_{\ \mathrm{T}}$	-0.710 ±0.052	-2.32 ±0.49	-5.79 ±1.61
(ROX) $^{AT}_{\ \mathrm{A}}$	-0.467 ±0.061	-4.02 ±0.91	-12.00 ±3.00
(A532) $^{AT}_{\ \mathrm{A}}$	-0.299 ±0.042	-1.86 ±0.66	-4.83 ±2.17
(ROX) $^{AC}_{\ \mathrm{G}}$	-0.662 ±0.058	-3.13 ±0.66	-7.81 ±2.16
(A532) $^{AC}_{\ \mathrm{G}}$	-0.607 ±0.062	-4.81 ±1.04	-13.38 ±3.44
(ROX) $^{AG}_{\ \mathrm{C}}$	-0.703 ±0.058	-1.95 ±0.94	-4.74 ±3.10
(A532) $^{AG}_{\ \mathrm{C}}$	-0.574 ±0.048	1.36 ±1.08	6.45 ±3.57
(ROX) $^{TA}_{\ \mathrm{T}}$	-0.337 ±0.102	-1.04 ±1.10	-2.28 ±3.63
(A532) $^{TA}_{\ \mathrm{T}}$	-0.501 ±0.074	-4.10 ±0.66	-11.52 ±2.19
(ROX) $^{TT}_{\ \mathrm{A}}$	-0.189 ±0.067	-2.59 ±0.72	-8.25 ±2.36
(A532) $^{TT}_{\ \mathrm{A}}$	-0.116 ±0.075	-1.71 ±1.12	-5.21 ±3.70
(ROX) $^{TC}_{\ \mathrm{G}}$	-0.308 ±0.074	-3.48 ±0.75	-10.26 ±2.46
(A532) $^{TC}_{\ \mathrm{G}}$	-0.416 ±0.070	-1.77 ±0.92	-4.49 ±3.02
(ROX) $^{TG}_{\ \mathrm{C}}$	-0.504 ±0.044	-0.35 ±0.85	0.40 ±2.82
(A532) $^{TG}_{\ \mathrm{C}}$	-0.579 ±0.030	1.71 ±0.74	7.24 ±2.45
(ROX) $^{CA}_{\ \mathrm{T}}$	-0.380 ±0.081	-5.31 ±1.01	-16.66 ±3.34
(A532) $^{CA}_{\ \mathrm{T}}$	-0.070 ±0.079	-1.40 ±1.10	-3.90 ±3.62
(ROX) $^{CT}_{\ \mathrm{A}}$	-0.026 ±0.065	-1.70 ±0.78	-5.38 ±2.57
(A532) $^{CT}_{\ \mathrm{A}}$	0.017 ±0.039	-1.71 ±0.71	-5.57 ±2.36
(ROX) $^{CC}_{\ \mathrm{G}}$	-0.456 ±0.090	1.25 ±0.71	5.95 ±2.35
(A532) $^{CC}_{\ \mathrm{G}}$	-0.243 ±0.054	-1.70 ±1.12	-4.25 ±3.69
(ROX) $^{CG}_{\ \mathrm{C}}$	-0.449 ±0.099	-0.96 ±1.43	-1.64 ±4.72
(A532) $^{CG}_{\ \mathrm{C}}$	-0.398 ±0.076	-1.07 ±0.59	-1.59 ±1.95
(ROX) $^{GA}_{\ \mathrm{T}}$	-0.412 ±0.043	-1.60 ±1.28	-3.96 ±4.23
(A532) $^{GA}_{\ \mathrm{T}}$	-0.274 ±0.061	-0.17 ±0.69	0.70 ±2.29
(ROX) $^{GT}_{\ \mathrm{A}}$	-0.196 ±0.059	-1.96 ±0.79	-5.93 ±2.60
(A532) $^{GT}_{\ \mathrm{A}}$	-0.110 ±0.062	0.76 ±0.80	2.98 ±2.65
(ROX) $^{GC}_{\ \mathrm{G}}$	-0.390 ±0.065	-1.96 ±0.77	-4.50 ±2.56
(A532) $^{GC}_{\ \mathrm{G}}$	-0.459 ±0.078	-2.19 ±0.95	-5.51 ±3.12
(ROX) $^{GG}_{\ \mathrm{C}}$	-0.664 ±0.048	-1.49 ±1.58	-2.62 ±5.21
(A532) $^{GG}_{\ \mathrm{C}}$	-0.436 ±0.028	-0.85 ±0.81	-1.19 ±2.69

Supplementary Table 10: ΔG° parameters at 37°C, and fitted ΔH°, and ΔS° values for 5′ single-base dangles.

	ΔG°_{37} (kcal/mol)	ΔH° (kcal/mol)	ΔS° (cal/mol·K)
(ROX) $^{AA}_{\mathrm{T}}$	0.033 ±0.083	-0.04 ±1.15	0.16 ±3.80
(A532) $^{AA}_{\mathrm{T}}$	-0.060 ±0.091	0.91 ±1.34	3.61 ±4.42
(ROX) $^{TA}_{\mathrm{A}}$	-0.443 ±0.148	-2.32 ±1.42	-6.48 ±4.67
(A532) $^{TA}_{\mathrm{A}}$	-0.093 ±0.061	-0.10 ±0.87	0.70 ±2.88
(ROX) $^{CA}_{\mathrm{G}}$	-0.546 ±0.093	-0.16 ±1.62	1.37 ±5.35
(A532) $^{CA}_{\mathrm{G}}$	-0.743 ±0.069	-4.24 ±1.05	-11.64 ±3.46
(ROX) $^{GA}_{\mathrm{C}}$	-0.117 ±0.075	2.63 ±1.32	8.96 ±4.36
(A532) $^{GA}_{\mathrm{C}}$	-0.118 ±0.064	1.93 ±0.60	6.52 ±1.98
(ROX) $^{AT}_{\mathrm{T}}$	0.191 ±0.067	-1.94 ±0.96	-6.89 ±3.17
(A532) $^{AT}_{\mathrm{T}}$	0.176 ±0.061	-1.35 ±0.92	-4.67 ±3.04
(ROX) $^{TT}_{\mathrm{A}}$	-0.042 ±0.187	-0.29 ±1.95	-1.26 ±6.44
(A532) $^{TT}_{\mathrm{A}}$	0.294 ±0.050	0.85 ±1.03	2.27 ±3.39
(ROX) $^{CT}_{\mathrm{G}}$	0.152 ±0.150	-0.08 ±1.50	0.17 ±4.95
(A532) $^{CT}_{\mathrm{G}}$	-0.061 ±0.064	-3.98 ±0.90	-12.38 ±2.97
(ROX) $^{GT}_{\mathrm{C}}$	0.171 ±0.094	3.86 ±1.40	11.62 ±4.61
(A532) $^{GT}_{\mathrm{C}}$	0.320 ±0.057	4.90 ±0.70	14.93 ±2.30
(ROX) $^{AC}_{\mathrm{T}}$	0.488 ±0.084	0.26 ±1.00	-0.28 ±3.31
(A532) $^{AC}_{\mathrm{T}}$	0.482 ±0.085	0.02 ±1.02	-1.05 ±3.36
(ROX) $^{TC}_{\mathrm{A}}$	-0.171 ±0.158	-0.97 ±2.39	-3.11 ±7.88
(A532) $^{TC}_{\mathrm{A}}$	0.188 ±0.046	-0.90 ±0.87	-3.26 ±2.87
(ROX) $^{CC}_{\mathrm{G}}$	0.042 ±0.144	1.50 ±1.95	5.55 ±6.43
(A532) $^{CC}_{\mathrm{G}}$	-0.131 ±0.059	-2.64 ±1.11	-7.94 ±3.67
(ROX) $^{GC}_{\mathrm{C}}$	-0.016 ±0.081	-1.00 ±1.12	-3.41 ±3.70
(A532) $^{GC}_{\mathrm{C}}$	0.114 ±0.130	2.00 ±0.71	6.32 ±2.35
(ROX) $^{AG}_{\mathrm{T}}$	0.243 ±0.091	1.27 ±1.86	3.77 ±6.15
(A532) $^{AG}_{\mathrm{T}}$	0.172 ±0.070	2.94 ±1.09	9.62 ±3.61
(ROX) $^{TG}_{\mathrm{A}}$	-0.212 ±0.146	-1.70 ±1.90	-5.39 ±6.26
(A532) $^{TG}_{\mathrm{A}}$	0.023 ±0.089	1.23 ±0.92	4.81 ±3.03
(ROX) $^{CG}_{\mathrm{G}}$	-0.320 ±0.088	0.17 ±1.52	2.34 ±5.00
(A532) $^{CG}_{\mathrm{G}}$	-0.686 ±0.056	-1.93 ±1.05	-4.22 ±3.45
(ROX) $^{GG}_{\mathrm{C}}$	-0.024 ±0.092	2.27 ±1.09	7.14 ±3.60
(A532) $^{GG}_{\mathrm{C}}$	0.126 ±0.060	2.85 ±0.71	9.05 ±2.33

Supplementary Table 11: ΔG° parameters at 37°C, and fitted ΔH°, and ΔS° values for 5′ single-base dangles.

	ΔG°_{10} (kcal/mol)	ΔG°_{25}	ΔG°_{37}	ΔG°_{45}	$\Delta G^\circ_{\mathrm{Mg},25}$
ROX−A/T vs. AA/T	0.325 ±0.051	0.371 ±0.037	0.258 ±0.084	-0.070 ±0.017	0.197 ±0.092
A532−A/T vs. AA/T	0.197 ±0.020	0.211 ±0.039	0.368 ±0.019	0.024 ±0.034	0.211 ±0.017
ROX−T/A vs. AT/A	0.536 ±0.044	0.364 ±0.035	0.266 ±0.021	-0.037 ±0.045	0.390 ±0.050
A532−T/A vs. AT/A	0.021 ±0.020	0.124 ±0.033	-0.057 ±0.021	-0.124 ±0.042	0.073 ±0.013
ROX−C/G vs. AC/G	0.645 ±0.044	0.399 ±0.026	0.295 ±0.031	0.187 ±0.013	0.422 ±0.030
A532−C/G vs. AC/G	0.398 ±0.059	0.385 ±0.037	0.179 ±0.047	0.042 ±0.054	0.279 ±0.033
ROX−G/C vs. AG/C	0.206 ±0.065	0.130 ±0.052	0.187 ±0.039	-0.128 ±0.019	0.362 ±0.013
A532−G/C vs. AG/C	-0.135 ±0.090	0.279 ±0.056	0.169 ±0.042	0.132 ±0.040	0.028 ±0.092
ROX−A/T vs. TA/T	0.051 ±0.025	0.205 ±0.043	0.061 ±0.092	0.093 ±0.051	0.246 ±0.010
A532−A/T vs. TA/T	0.336 ±0.029	0.409 ±0.049	0.159 ±0.056	0.015 ±0.090	0.025 ±0.107
ROX−T/A vs. TT/A	0.196 ±0.026	0.013 ±0.050	-0.012 ±0.034	-0.202 ±0.023	0.079 ±0.056
A532−T/A vs. TT/A	-0.117 ±0.054	-0.399 ±0.061	-0.240 ±0.065	-0.275 ±0.085	-0.327 ±0.026
ROX−C/G vs. TC/G	0.262 ±0.023	0.126 ±0.058	-0.058 ±0.056	-0.299 ±0.052	0.202 ±0.040
A532−C/G vs. TC/G	-0.043 ±0.040	-0.150 ±0.025	-0.012 ±0.057	-0.124 ±0.072	-0.150 ±0.016
ROX−G/C vs. TG/C	0.161 ±0.017	0.070 ±0.010	-0.011 ±0.011	-0.037 ±0.025	0.169 ±0.014
A532−G/C vs. TG/C	-0.164 ±0.072	0.036 ±0.061	0.174 ±0.018	-0.012 ±0.002	-0.081 ±0.030
ROX−A/T vs. CA/T	0.200 ±0.044	0.206 ±0.065	0.104 ±0.069	-0.254 ±0.014	0.010 ±0.022
A532−A/T vs. CA/T	-0.132 ±0.065	-0.150 ±0.057	-0.272 ±0.063	-0.134 ±0.061	-0.122 ±0.007
ROX−T/A vs. CT/A	0.119 ±0.045	-0.008 ±0.088	-0.175 ±0.031	-0.141 ±0.005	0.016 ±0.029
A532−T/A vs. CT/A	-0.275 ±0.026	-0.358 ±0.014	-0.373 ±0.013	-0.401 ±0.081	-0.350 ±0.028
ROX−C/G vs. CC/G	0.167 ±0.022	0.111 ±0.017	0.089 ±0.076	0.242 ±0.052	0.207 ±0.084
A532−C/G vs. CC/G	-0.096 ±0.063	0.025 ±0.059	-0.185 ±0.036	-0.030 ±0.065	-0.192 ±0.049
ROX−G/C vs. CG/C	0.207 ±0.043	0.079 ±0.079	-0.066 ±0.089	-0.070 ±0.115	0.136 ±0.023
A532−G/C vs. CG/C	0.268 ±0.015	0.173 ±0.074	-0.007 ±0.072	0.050 ±0.037	0.100 ±0.018
ROX−A/T vs. GA/T	0.135 ±0.132	0.191 ±0.064	0.136 ±0.002	0.116 ±0.039	0.206 ±0.036
A532−A/T vs. GA/T	-0.084 ±0.058	0.077 ±0.013	-0.069 ±0.036	0.035 ±0.015	0.014 ±0.078
ROX−T/A vs. GT/A	0.287 ±0.025	0.010 ±0.029	-0.005 ±0.013	0.026 ±0.067	0.002 ±0.031
A532−T/A vs. GT/A	-0.357 ±0.042	-0.225 ±0.058	-0.246 ±0.050	-0.249 ±0.025	-0.197 ±0.073
ROX−C/G vs. GC/G	0.425 ±0.047	0.210 ±0.035	0.023 ±0.043	0.099 ±0.027	0.269 ±0.025
A532−C/G vs. GC/G	0.121 ±0.122	0.022 ±0.040	0.031 ±0.066	-0.079 ±0.016	0.098 ±0.061
ROX−G/C vs. GG/C	0.451 ±0.090	0.310 ±0.022	0.149 ±0.023	0.237 ±0.173	0.303 ±0.016
A532−G/C vs. GG/C	0.066 ±0.042	0.153 ±0.034	0.032 ±0.014	-0.041 ±0.069	0.027 ±0.017

Supplementary Table 12: Raw ΔG° values for $5'$ single-base dangles directly calculated from the second reaction.

	ΔG°_{10} (kcal/mol)	ΔG°_{25}	ΔG°_{37}	ΔG°_{45}	$\Delta G^\circ_{\mathrm{Mg},25}$
A−ROX / T vs. AA / T	-0.659 ±0.104	-0.500 ±0.041	-0.483 ±0.065	-0.432 ±0.032	-0.208 ±0.060
A−A532 / T vs. AA / T	-0.222 ±0.060	-0.006 ±0.053	-0.024 ±0.070	0.342 ±0.108	-0.022 ±0.079
T−ROX / A vs. TA / A	-0.751 ±0.043	-0.588 ±0.061	-0.885 ±0.044	-0.533 ±0.054	-0.580 ±0.077
T−A532 / A vs. TA / A	-0.001 ±0.040	0.111 ±0.076	-0.132 ±0.048	0.149 ±0.045	0.044 ±0.109
C−ROX / G vs. CA / G	-0.428 ±0.157	-0.334 ±0.027	-0.343 ±0.056	-0.379 ±0.031	-0.190 ±0.053
C−A532 / G vs. CA / G	0.752 ±0.064	0.522 ±0.091	0.495 ±0.047	0.464 ±0.019	0.443 ±0.051
G−ROX / C vs. GA / C	-0.760 ±0.062	-0.628 ±0.043	-0.575 ±0.037	-0.565 ±0.041	-0.374 ±0.035
G−A532 / C vs. GA / C	-0.355 ±0.019	-0.131 ±0.040	-0.087 ±0.034	-0.152 ±0.040	-0.321 ±0.047
A−ROX / T vs. AT / T	-0.630 ±0.043	-0.612 ±0.124	-0.642 ±0.042	-0.790 ±0.037	-0.587 ±0.030
A−A532 / T vs. AT / T	-0.280 ±0.037	-0.400 ±0.074	-0.260 ±0.020	-0.169 ±0.032	-0.114 ±0.100
T−ROX / A vs. TT / A	-1.314 ±0.089	-1.007 ±0.070	-1.285 ±0.122	-0.915 ±0.103	-0.960 ±0.043
T−A532 / A vs. TT / A	-0.554 ±0.047	-0.321 ±0.050	-0.519 ±0.033	-0.091 ±0.104	-0.187 ±0.065
C−ROX / G vs. CT / G	-0.866 ±0.048	-0.767 ±0.030	-1.041 ±0.131	-0.809 ±0.037	-0.570 ±0.072
C−A532 / G vs. CT / G	0.224 ±0.036	0.252 ±0.041	-0.187 ±0.039	0.007 ±0.020	0.022 ±0.091
G−ROX / C vs. GT / C	-1.196 ±0.057	-1.166 ±0.052	-0.863 ±0.067	-1.004 ±0.085	-0.931 ±0.058
G−A532 / C vs. GT / C	-0.949 ±0.039	-0.658 ±0.036	-0.525 ±0.018	-0.414 ±0.029	-0.788 ±0.027
A−ROX / T vs. AC / T	-0.981 ±0.063	-0.890 ±0.064	-0.938 ±0.066	-0.859 ±0.028	-0.564 ±0.005
A−A532 / T vs. AC / T	-0.668 ±0.038	-0.572 ±0.110	-0.566 ±0.063	-0.392 ±0.057	-0.620 ±0.026
T−ROX / A vs. TC / A	-1.186 ±0.099	-0.899 ±0.153	-1.156 ±0.072	-0.989 ±0.218	-0.756 ±0.055
T−A532 / A vs. TC / A	-0.333 ±0.045	-0.295 ±0.060	-0.413 ±0.026	-0.383 ±0.039	-0.236 ±0.016
C−ROX / G vs. CC / G	-0.961 ±0.086	-0.685 ±0.083	-0.931 ±0.123	-0.648 ±0.081	-0.479 ±0.213
C−A532 / G vs. CC / G	0.296 ±0.054	0.024 ±0.063	-0.117 ±0.029	0.187 ±0.048	-0.057 ±0.025
G−ROX / C vs. GC / C	-0.580 ±0.027	-0.814 ±0.038	-0.676 ±0.048	-0.887 ±0.078	-0.522 ±0.034
G−A532 / C vs. GC / C	-0.496 ±0.028	-0.259 ±0.032	-0.319 ±0.118	-0.288 ±0.048	-0.419 ±0.052
A−ROX / T vs. AG / T	-1.295 ±0.201	-0.751 ±0.036	-0.693 ±0.075	-0.595 ±0.086	-0.656 ±0.029
A−A532 / T vs. AG / T	-0.544 ±0.052	-0.222 ±0.026	-0.256 ±0.039	0.170 ±0.055	-0.345 ±0.024
T−ROX / A vs. TG / A	-1.132 ±0.099	-0.752 ±0.033	-1.115 ±0.037	-0.877 ±0.089	-0.641 ±0.057
T−A532 / A vs. TG / A	-0.256 ±0.040	0.138 ±0.055	-0.248 ±0.081	-0.012 ±0.059	-0.102 ±0.044
C−ROX / G vs. CG / G	-0.592 ±0.041	-0.257 ±0.032	-0.569 ±0.047	-0.325 ±0.049	-0.284 ±0.149
C−A532 / G vs. CG / G	0.559 ±0.029	0.519 ±0.041	0.438 ±0.023	0.448 ±0.153	0.344 ±0.064
G−ROX / C vs. GG / C	-0.922 ±0.027	-0.761 ±0.072	-0.668 ±0.064	-0.880 ±0.042	-0.651 ±0.018
G−A532 / C vs. GG / C	-0.555 ±0.035	-0.352 ±0.027	-0.330 ±0.026	-0.219 ±0.035	-0.498 ±0.015

Supplementary Table 13: Raw ΔG° values for 3′ single-base dangles directly calculated from the second reaction with ROX and A532, respectively.

	ΔG°_{37} (kcal/mol)	ΔH° (kcal/mol)	ΔS° (cal/mol·K)
$\frac{ROX-A}{T}$ vs. $\frac{AA}{T}$	0.258 ±0.084	4.74 ±0.44	15.07 ±1.45
$\frac{A532-A}{T}$ vs. $\frac{AA}{T}$	0.368 ±0.019	-0.23 ±0.37	-1.57 ±1.23
$\frac{ROX-T}{A}$ vs. $\frac{AT}{A}$	0.266 ±0.021	4.28 ±0.64	13.09 ±2.12
$\frac{A532-T}{A}$ vs. $\frac{AT}{A}$	-0.057 ±0.021	0.99 ±0.38	3.32 ±1.25
$\frac{ROX-C}{G}$ vs. $\frac{AC}{G}$	0.295 ±0.031	3.98 ±0.34	11.94 ±1.13
$\frac{A532-C}{G}$ vs. $\frac{AC}{G}$	0.179 ±0.047	3.68 ±0.89	11.27 ±2.93
$\frac{ROX-G}{C}$ vs. $\frac{AG}{C}$	0.187 ±0.039	3.62 ±0.50	11.66 ±1.63
$\frac{A532-G}{C}$ vs. $\frac{AG}{C}$	0.169 ±0.042	-0.49 ±0.90	-2.09 ±2.96
$\frac{ROX-A}{T}$ vs. $\frac{TA}{T}$	0.061 ±0.092	-0.49 ±0.50	-1.97 ±1.64
$\frac{A532-A}{T}$ vs. $\frac{TA}{T}$	0.159 ±0.056	2.11 ±0.59	6.19 ±1.93
$\frac{ROX-T}{A}$ vs. $\frac{TT}{A}$	-0.012 ±0.034	3.27 ±0.41	10.82 ±1.37
$\frac{A532-T}{A}$ vs. $\frac{TT}{A}$	-0.240 ±0.065	1.07 ±0.99	4.40 ±3.27
$\frac{ROX-C}{G}$ vs. $\frac{TC}{G}$	-0.058 ±0.056	4.36 ±0.46	14.43 ±1.50
$\frac{A532-C}{G}$ vs. $\frac{TC}{G}$	-0.012 ±0.057	0.24 ±0.73	1.18 ±2.40
$\frac{ROX-G}{C}$ vs. $\frac{TG}{C}$	-0.011 ±0.011	1.90 ±0.27	6.14 ±0.90
$\frac{A532-G}{C}$ vs. $\frac{TG}{C}$	0.174 ±0.018	0.45 ±0.06	1.43 ±0.20
$\frac{ROX-A}{T}$ vs. $\frac{CA}{T}$	0.104 ±0.069	4.35 ±0.38	14.43 ±1.24
$\frac{A532-A}{T}$ vs. $\frac{CA}{T}$	-0.272 ±0.063	0.26 ±1.03	1.42 ±3.39
$\frac{ROX-T}{A}$ vs. $\frac{CT}{A}$	-0.175 ±0.031	0.94 ±0.14	3.40 ±0.46
$\frac{A532-T}{A}$ vs. $\frac{CT}{A}$	-0.373 ±0.013	0.56 ±0.41	3.03 ±1.37
$\frac{ROX-C}{G}$ vs. $\frac{CC}{G}$	0.089 ±0.076	0.27 ±0.43	0.44 ±1.41
$\frac{A532-C}{G}$ vs. $\frac{CC}{G}$	-0.185 ±0.036	0.51 ±0.96	2.02 ±3.17
$\frac{ROX-G}{C}$ vs. $\frac{CG}{C}$	-0.066 ±0.089	2.72 ±0.87	8.88 ±2.86
$\frac{A532-G}{C}$ vs. $\frac{CG}{C}$	-0.007 ±0.072	2.16 ±0.30	6.68 ±1.00
$\frac{ROX-A}{T}$ vs. $\frac{GA}{T}$	0.136 ±0.002	0.07 ±0.14	-0.20 ±0.46
$\frac{A532-A}{T}$ vs. $\frac{GA}{T}$	-0.069 ±0.036	0.46 ±0.37	1.34 ±1.23
$\frac{ROX-T}{A}$ vs. $\frac{GT}{A}$	-0.005 ±0.013	2.99 ±0.43	9.68 ±1.41
$\frac{A532-T}{A}$ vs. $\frac{GT}{A}$	-0.246 ±0.050	-1.09 ±0.55	-2.67 ±1.81
$\frac{ROX-C}{G}$ vs. $\frac{GC}{G}$	0.023 ±0.043	2.87 ±0.59	8.83 ±1.93
$\frac{A532-C}{G}$ vs. $\frac{GC}{G}$	0.031 ±0.066	1.68 ±0.45	5.52 ±1.49
$\frac{ROX-G}{C}$ vs. $\frac{GG}{C}$	0.149 ±0.023	3.94 ±0.96	12.20 ±3.17
$\frac{A532-G}{C}$ vs. $\frac{GG}{C}$	0.032 ±0.014	1.03 ±0.57	3.19 ±1.89

Supplementary Table 14: ΔG° values for 5′ single-base dangles at 37°C calculated in second reaction; fitted ΔH°, and ΔS° values for 5′ single-base dangles are also listed in the table. The data were calculated from fluorophores ROX and A532, respectively.

	ΔG°_{37} (kcal/mol)	ΔH° (kcal/mol)	ΔS° (cal/mol·K)
$A-ROX$ / T vs. AA / T	-0.483 ±0.065	-1.88 ±0.81	-4.55 ±2.68
$A-A532$ / T vs. AA / T	-0.024 ±0.070	-3.48 ±1.11	-11.52 ±3.68
$T-ROX$ / A vs. TA / A	-0.885 ±0.044	-1.21 ±0.75	-1.61 ±2.47
$T-A532$ / A vs. TA / A	-0.132 ±0.048	-0.51 ±0.69	-1.75 ±2.27
$C-ROX$ / G vs. CA / G	-0.343 ±0.056	0.18 ±0.78	1.73 ±2.57
$C-A532$ / G vs. CA / G	0.495 ±0.047	2.80 ±0.50	7.36 ±1.64
$G-ROX$ / C vs. GA / C	-0.575 ±0.037	-2.20 ±0.80	-5.18 ±2.63
$G-A532$ / C vs. GA / C	-0.087 ±0.034	-2.54 ±0.38	-7.76 ±1.24
$A-ROX$ / T vs. AT / T	-0.642 ±0.042	0.50 ±0.67	3.90 ±2.20
$A-A532$ / T vs. AT / T	-0.260 ±0.020	-1.07 ±0.54	-2.68 ±1.77
$T-ROX$ / A vs. TT / A	-1.285 ±0.122	-3.56 ±1.52	-8.18 ±5.00
$T-A532$ / A vs. TT / A	-0.519 ±0.033	-1.32 ±0.84	-2.85 ±2.79
$C-ROX$ / G vs. CT / G	-1.041 ±0.131	-0.94 ±0.71	-0.44 ±2.33
$C-A532$ / G vs. CT / G	-0.187 ±0.039	2.37 ±0.45	7.54 ±1.49
$G-ROX$ / C vs. GT / C	-0.863 ±0.067	-3.64 ±1.02	-8.53 ±3.38
$G-A532$ / C vs. GT / C	-0.525 ±0.018	-5.10 ±0.51	-14.77 ±1.70
$A-ROX$ / T vs. AC / T	-0.938 ±0.066	-1.86 ±0.67	-3.13 ±2.20
$A-A532$ / T vs. AC / T	-0.566 ±0.063	-2.58 ±0.72	-6.71 ±2.37
$T-ROX$ / A vs. TC / A	-1.156 ±0.072	-1.55 ±1.81	-1.41 ±5.98
$T-A532$ / A vs. TC / A	-0.413 ±0.026	0.34 ±0.66	2.35 ±2.18
$C-ROX$ / G vs. CC / G	-0.931 ±0.123	-2.87 ±1.39	-6.91 ±4.58
$C-A532$ / G vs. CC / G	-0.117 ±0.029	2.16 ±0.78	6.98 ±2.59
$G-ROX$ / C vs. GC / C	-0.676 ±0.048	1.35 ±0.55	6.91 ±1.80
$G-A532$ / C vs. GC / C	-0.319 ±0.118	-2.45 ±0.54	-7.03 ±1.79
$A-ROX$ / T vs. AG / T	-0.693 ±0.075	-3.76 ±1.64	-10.02 ±5.40
$A-A532$ / T vs. AG / T	-0.256 ±0.039	-4.65 ±0.79	-14.68 ±2.60
$T-ROX$ / A vs. TG / A	-1.115 ±0.037	2.10 ±1.20	9.96 ±3.95
$T-A532$ / A vs. TG / A	-0.248 ±0.081	-1.94 ±0.74	-6.15 ±2.45
$C-ROX$ / G vs. CG / G	-0.569 ±0.047	-1.67 ±0.69	-4.19 ±2.28
$C-A532$ / G vs. CG / G	0.438 ±0.023	1.83 ±0.55	4.46 ±1.83
$G-ROX$ / C vs. GG / C	-0.668 ±0.064	-1.68 ±0.51	-2.73 ±1.69
$G-A532$ / C vs. GG / C	-0.330 ±0.026	-2.90 ±0.54	-8.39 ±1.79

Supplementary Table 15: ΔG°, ΔH°, and ΔS° values for $3'$ single-base dangles calculated with ROX and A532 in the second reaction, respectively.

Length (nt)	$\substack{ROX-A \\ T}$ vs. $\substack{nTAA \\ T}$	$\substack{A532-A \\ T}$ vs. $\substack{nTAA \\ T}$	$\substack{ROX-A \\ T}$ vs. $\substack{nTCA \\ T}$	$\substack{A532-A \\ T}$ vs. $\substack{nTCA \\ T}$	$\substack{ROX-A \\ T}$ vs. $\substack{nTGA \\ T}$	$\substack{A532-A \\ T}$ vs. $\substack{nTGA \\ T}$
0	-0.216 ±0.038	-0.303 ±0.031	-0.216 ±0.038	-0.303 ±0.031	-0.216 ±0.038	-0.303 ±0.031
1	0.371 ±0.037	0.211 ±0.039	0.164 ±0.091	-0.150 ±0.057	0.191 ±0.064	0.077 ±0.013
2	0.367 ±0.027	0.208 ±0.030	0.161 ±0.021	-0.029 ±0.047	0.214 ±0.045	-0.074 ±0.036
3	0.152 ±0.022	0.007 ±0.050	-0.023 ±0.063	-0.258 ±0.046	-0.368 ±0.043	-0.626 ±0.064
4	0.252 ±0.056	-0.016 ±0.037	-0.312 ±0.047	-0.189 ±0.016	-0.874 ±0.063	-1.215 ±0.091
5	-0.155 ±0.075	-0.290 ±0.039	-0.296 ±0.017	-0.432 ±0.106	-1.060 ±0.048	-1.380 ±0.095
6	-0.068 ±0.044	-0.218 ±0.038	-0.328 ±0.076	-0.224 ±0.023	-0.831 ±0.059	-0.725 ±0.060
7	-0.072 ±0.030	-0.273 ±0.036	-0.521 ±0.062	-0.193 ±0.031	-0.762 ±0.035	-0.877 ±0.074
9	-0.177 ±0.039	-0.366 ±0.050	-0.440 ±0.043	-0.365 ±0.096	-0.658 ±0.057	-1.000 ±0.036
11	-0.233 ±0.039	-0.414 ±0.042	-0.415 ±0.056	-0.417 ±0.029	-0.581 ±0.078	-0.947 ±0.093
13	-0.035 ±0.043	-0.092 ±0.032	-0.343 ±0.095	-0.520 ±0.058	-0.615 ±0.042	-0.509 ±0.050
16	-0.167 ±0.032	-0.227 ±0.053	-0.429 ±0.033	-0.499 ±0.052	-0.634 ±0.044	-0.764 ±0.054
21	-0.157 ±0.063	-0.169 ±0.036	-0.400 ±0.074	-0.236 ±0.044	-0.694 ±0.053	-0.639 ±0.062

Supplementary Table 16: Raw ΔG° values for $5'$ multibase dangle parameters at 25°C in 1xPBS buffer.

Length(nt)	$\substack{A-ROX \\ T}$ vs. $\substack{AATn \\ T}$	$\substack{A-A532 \\ T}$ vs. $\substack{AATn \\ T}$	$\substack{A-ROX \\ T}$ vs. $\substack{ACTn \\ T}$	$\substack{A-A532 \\ T}$ vs. $\substack{ACTn \\ T}$	$\substack{A-ROX \\ T}$ vs. $\substack{AGTn \\ T}$	$\substack{A-A532 \\ T}$ vs. $\substack{AGTn \\ T}$
0	-0.654 ±0.028	-0.135 ±0.048	-0.654 ±0.028	-0.135 ±0.048	-0.654 ±0.028	-0.135 ±0.048
1	-0.500 ±0.041	-0.006 ±0.053	-0.890 ±0.064	-0.572 ±0.110	-0.751 ±0.036	-0.222 ±0.026
2	-0.723 ±0.048	-0.396 ±0.025	-1.169 ±0.046	-0.621 ±0.066	-1.563 ±0.098	-0.882 ±0.042
3	-0.782 ±0.058	-0.522 ±0.058	-0.773 ±0.041	-0.639 ±0.048	-1.871 ±0.061	-0.936 ±0.117
4	-0.775 ±0.031	-0.691 ±0.020	-0.968 ±0.053	-0.659 ±0.054	-1.764 ±0.061	-1.190 ±0.047
5	-1.055 ±0.058	-0.842 ±0.027	-1.325 ±0.129	-0.956 ±0.047	-1.884 ±0.040	-1.351 ±0.081
6	-0.828 ±0.063	-0.341 ±0.040	-1.083 ±0.045	-0.658 ±0.048	-1.533 ±0.085	-1.094 ±0.088
7	-1.083 ±0.048	-0.612 ±0.040	-1.323 ±0.091	-0.834 ±0.103	-1.954 ±0.114	-0.947 ±0.079
9	-1.430 ±0.032	-0.976 ±0.057	-1.305 ±0.040	-0.689 ±0.048	-1.596 ±0.186	-0.926 ±0.042
11	-1.272 ±0.059	-0.861 ±0.053	-1.343 ±0.071	-0.824 ±0.051	-1.361 ±0.044	-0.807 ±0.064
13	-1.221 ±0.047	-0.775 ±0.046	-1.243 ±0.075	-0.605 ±0.038	-1.258 ±0.069	-1.211 ±0.018
16	-1.266 ±0.080	-0.930 ±0.058	-1.703 ±0.088	-0.789 ±0.075	-1.961 ±0.155	-1.064 ±0.061
21	-0.980 ±0.089	-0.746 ±0.039	-1.494 ±0.073	-1.205 ±0.042	-1.645 ±0.095	-1.135 ±0.069

Supplementary Table 17: Raw ΔG° values for $3'$ multibase dangle parameters at 25°C in 1xPBS buffer.

Length(nt)	$\substack{ROX-A \\ T}$ vs. $\substack{(n-1)TTA \\ T}$	$\substack{A532-A \\ T}$ vs. $\substack{(n-1)TTA \\ T}$	$\substack{A-ROX \\ T}$ vs. $\substack{ATT(n-1) \\ T}$	$\substack{A-A532 \\ T}$ vs. $\substack{ATT(n-1) \\ T}$
0	-0.216 ±0.038	-0.303 ±0.031	-0.654 ±0.028	-0.135 ±0.048
1	0.267 ±0.017	0.409 ±0.049	-0.612 ±0.124	-0.400 ±0.074
2	0.274 ±0.055	0.345 ±0.030	-1.024 ±0.072	-0.821 ±0.054
3	0.103 ±0.056	-0.254 ±0.055	-1.269 ±0.093	-0.692 ±0.022
4	-0.076 ±0.072	-0.158 ±0.025	-1.285 ±0.088	-0.633 ±0.058
5	-0.196 ±0.018	-0.915 ±0.050	-1.432 ±0.103	-0.845 ±0.037
6	-0.271 ±0.026	-0.473 ±0.082	-1.144 ±0.067	-0.673 ±0.055
7	-0.288 ±0.021	-0.545 ±0.034	-1.519 ±0.064	-0.906 ±0.055
8	-0.433 ±0.039	-0.873 ±0.078	-1.297 ±0.067	-0.897 ±0.059
10	-0.250 ±0.027	-1.015 ±0.073	-1.195 ±0.039	-0.956 ±0.052
12	-0.295 ±0.048	-0.361 ±0.043	-1.458 ±0.055	-0.941 ±0.021
15	-0.272 ±0.048	-0.492 ±0.059	-1.313 ±0.066	-1.080 ±0.034
20	-0.214 ±0.029	-0.532 ±0.057	-1.344 ±0.080	-0.988 ±0.065

Supplementary Table 18: Raw ΔG° values for multibase dangle parameters when dangles are thymines in 1xPBS buffer.

Length (nt)	$nTAA$ T	$nTAA$ T	$nTCA$ T	$nTCA$ T	$nTGA$ T	$nTGA$ T
1	-0.587 ±0.053	-0.514 ±0.050	-0.380 ±0.099	-0.153 ±0.065	-0.408 ±0.074	-0.380 ±0.034
2	-0.583 ±0.047	-0.511 ±0.043	-0.377 ±0.043	-0.274 ±0.056	-0.430 ±0.059	-0.230 ±0.047
3	-0.368 ±0.044	-0.310 ±0.059	-0.193 ±0.073	-0.045 ±0.056	0.152 ±0.058	0.323 ±0.071
4	-0.468 ±0.068	-0.287 ±0.048	0.096 ±0.061	-0.114 ±0.035	0.657 ±0.073	0.912 ±0.096
5	-0.061 ±0.084	-0.013 ±0.050	0.079 ±0.041	0.129 ±0.110	0.844 ±0.062	1.077 ±0.100
6	-0.148 ±0.058	-0.085 ±0.049	0.112 ±0.085	-0.079 ±0.039	0.615 ±0.070	0.422 ±0.068
7	-0.145 ±0.048	-0.030 ±0.047	0.305 ±0.073	-0.110 ±0.044	0.546 ±0.052	0.574 ±0.080
9	-0.039 ±0.054	0.063 ±0.059	0.224 ±0.057	0.062 ±0.101	0.442 ±0.068	0.697 ±0.047
11	0.017 ±0.054	0.111 ±0.052	0.199 ±0.068	0.113 ±0.042	0.365 ±0.086	0.643 ±0.098
13	-0.181 ±0.057	-0.211 ±0.045	0.127 ±0.102	0.217 ±0.066	0.399 ±0.056	0.206 ±0.059
16	-0.049 ±0.050	-0.076 ±0.062	0.213 ±0.051	0.196 ±0.060	0.418 ±0.058	0.461 ±0.063
21	-0.059 ±0.074	-0.134 ±0.048	0.184 ±0.083	-0.068 ±0.054	0.478 ±0.065	0.336 ±0.070

Supplementary Table 19: Real ΔG° values for $5'$ multibase dangle parameters at 25°C 1xPBS buffer.

Length(nt)	$AATn$ T	$AATn$ T	$ACTn$ T	$ACTn$ T	$AGTn$ T	$AGTn$ T
1	-0.154 ±0.050	-0.128 ±0.071	0.236 ±0.070	0.438 ±0.120	0.097 ±0.045	0.088 ±0.054
2	0.069 ±0.056	0.261 ±0.054	0.514 ±0.054	0.486 ±0.082	0.909 ±0.102	0.747 ±0.063
3	0.128 ±0.064	0.387 ±0.075	0.119 ±0.050	0.504 ±0.067	1.217 ±0.067	0.802 ±0.127
4	0.120 ±0.042	0.556 ±0.052	0.314 ±0.060	0.525 ±0.072	1.110 ±0.067	1.055 ±0.067
5	0.400 ±0.065	0.707 ±0.055	0.670 ±0.132	0.821 ±0.067	1.229 ±0.049	1.217 ±0.094
6	0.173 ±0.069	0.207 ±0.062	0.429 ±0.053	0.523 ±0.067	0.879 ±0.090	0.959 ±0.100
7	0.428 ±0.055	0.477 ±0.063	0.669 ±0.096	0.700 ±0.113	1.300 ±0.117	0.812 ±0.092
9	0.775 ±0.043	0.841 ±0.074	0.650 ±0.049	0.554 ±0.068	0.941 ±0.188	0.791 ±0.064
11	0.618 ±0.065	0.727 ±0.071	0.689 ±0.077	0.689 ±0.069	0.707 ±0.052	0.672 ±0.080
13	0.567 ±0.054	0.641 ±0.066	0.589 ±0.080	0.470 ±0.061	0.603 ±0.075	1.077 ±0.051
16	0.612 ±0.085	0.795 ±0.075	1.048 ±0.092	0.654 ±0.089	1.306 ±0.157	0.929 ±0.078
21	0.326 ±0.094	0.611 ±0.062	0.840 ±0.078	1.070 ±0.064	0.991 ±0.099	1.000 ±0.084

Supplementary Table 20: Real ΔG° values for $3'$ multibase dangle parameters at 25°C 1xPBS buffer.

Length (nt)	$(n-1)TTA$ T	$(n-1)TTA$ T	$ATT(n-1)$ T	$ATT(n-1)$ T
1	-0.484 ±0.042	-0.712 ±0.059	-0.043 ±0.127	0.265 ±0.088
2	-0.490 ±0.067	-0.648 ±0.044	0.369 ±0.077	0.686 ±0.072
3	-0.319 ±0.067	-0.050 ±0.063	0.614 ±0.097	0.558 ±0.052
4	-0.140 ±0.082	-0.146 ±0.040	0.630 ±0.092	0.498 ±0.075
5	-0.020 ±0.042	0.611 ±0.059	0.777 ±0.106	0.710 ±0.060
6	0.055 ±0.046	0.170 ±0.088	0.489 ±0.072	0.538 ±0.073
7	0.072 ±0.043	0.241 ±0.046	0.865 ±0.070	0.771 ±0.073
8	0.217 ±0.055	0.570 ±0.084	0.642 ±0.072	0.762 ±0.076
10	0.034 ±0.047	0.712 ±0.079	0.540 ±0.048	0.822 ±0.070
12	0.079 ±0.061	0.058 ±0.053	0.803 ±0.062	0.807 ±0.052
15	0.055 ±0.061	0.189 ±0.067	0.658 ±0.072	0.946 ±0.059
20	-0.002 ±0.048	0.229 ±0.065	0.690 ±0.085	0.853 ±0.081

Supplementary Table 21: Real ΔG° values for multibase dangle parameters when dangles are thymines at 25°C 1xPBS buffer.

Length (nt)	$nTAA$ T	$nTCA$ T	$nTGA$ T	$AATn$ T	$ACTn$ T	$AGTn$ T
1	-0.549 ±0.045 (9.0)	-0.243 ±0.101 (6.2)	-0.389 ±0.042 (9.9)	-0.144 ±0.045 (9.9)	0.310 ±0.101 (7.7)	0.093 ±0.036 (10.0)
2	-0.545 ±0.041 (8.7)	-0.332 ±0.051 (7.9)	-0.319 ±0.081 (2.9)	0.167 ±0.078 (3.9)	0.503 ±0.050 (9.9)	0.809 ±0.084 (8.1)
3	-0.343 ±0.042 (9.4)	-0.109 ±0.070 (7.3)	0.228 ±0.076 (6.5)	0.248 ±0.104 (3.1)	0.283 ±0.144 (-11.5)	1.073 ±0.169 (0.8)
4	-0.363 ±0.077 (5.1)	-0.037 ±0.085 (0.3)	0.768 ±0.109 (5.5)	0.315 ±0.159 (-33.7)	0.410 ±0.088 (4.9)	1.082 ±0.051 (9.7)
5	-0.031 ±0.052 (9.7)	0.093 ±0.062 (9.8)	0.932 ±0.103 (5.8)	0.567 ±0.117 (-3.2)	0.770 ±0.093 (8.9)	1.225 ±0.053 (10.0)
6	-0.114 ±0.044 (9.3)	-0.019 ±0.086 (5.2)	0.517 ±0.084 (6.1)	0.191 ±0.048 (9.9)	0.470 ±0.055 (8.8)	0.917 ±0.073 (9.6)
7	-0.087 ±0.053 (7.1)	0.046 ±0.157 (-15.2)	0.557 ±0.049 (9.9)	0.451 ±0.045 (9.7)	0.683 ±0.075 (10.0)	1.027 ±0.189 (-0.9)
9	0.010 ±0.054 (8.4)	0.165 ±0.083 (7.9)	0.592 ±0.101 (0.3)	0.800 ±0.049 (9.4)	0.610 ±0.054 (8.7)	0.829 ±0.116 (9.3)
11	0.065 ±0.050 (8.5)	0.146 ±0.051 (8.8)	0.495 ±0.118 (5.4)	0.670 ±0.062 (8.7)	0.689 ±0.052 (10.0)	0.693 ±0.049 (9.9)
13	-0.198 ±0.038 (9.8)	0.181 ±0.069 (9.4)	0.305 ±0.080 (4.4)	0.600 ±0.050 (9.2)	0.522 ±0.066 (8.6)	0.884 ±0.176 (-18.4)
16	-0.061 ±0.041 (9.9)	0.205 ±0.040 (10.0)	0.439 ±0.045 (9.8)	0.709 ±0.086 (7.4)	0.848 ±0.153 (0.5)	1.054 ±0.166 (4.8)
21	-0.104 ±0.052 (9.2)	0.032 ±0.103 (3.3)	0.409 ±0.069 (7.8)	0.498 ±0.117 (3.2)	0.967 ±0.096 (4.7)	0.996 ±0.065 (10.0)

Supplementary Table 22: Consensus ΔG° values for multibase dangle parameters at 25°C in 1xPBS buffer.

Length(nt)	$^{(n-1)TTA}_{T}$	$^{ATT(n-1)}_{T}$
1	-0.579 ±0.089 (-0.4)	0.139 ±0.135 (5.9)
2	-0.586 ±0.070 (6.0)	0.534 ±0.124 (0.9)
3	-0.180 ±0.106 (1.5)	0.578 ±0.059 (9.7)
4	-0.144 ±0.046 (10.0)	0.557 ±0.076 (8.8)
5	0.242 ±0.229 (-67.9)	0.734 ±0.066 (9.7)
6	0.094 ±0.065 (8.5)	0.514 ±0.054 (9.8)
7	0.154 ±0.068 (2.8)	0.819 ±0.060 (9.1)
8	0.356 ±0.137 (-2.9)	0.701 ±0.068 (8.7)
10	0.285 ±0.252 (-48.2)	0.655 ±0.110 (-1.2)
12	0.068 ±0.041 (9.9)	0.805 ±0.041 (10.0)
15	0.119 ±0.066 (7.8)	0.817 ±0.112 (0.3)
20	0.096 ±0.092 (1.6)	0.773 ±0.082 (8.1)

Supplementary Table 23: Consensus ΔG° values for multibase thymine dangle parameters at 25°C in 1xPBS buffer.

Length (nt)	(A532) $^{nAA}_{T}$	(ROX) $^{nAA}_{T}$	$^{nAA}_{T}$
1	-0.534 ±0.0498	-0.587 ±0.0529	-0.549 ±0.045 (9.0)
2	-0.491 ±0.0930	-0.558 ±0.0428	-0.529 ±0.060 (9.2)
3	-0.304 ±0.0430	-0.355 ±0.0424	-0.320 ±0.040 (8.6)
4	-0.187 ±0.0364	-0.394 ±0.0581	-0.254 ±0.089 (-1.6)
6	-0.250 ±0.0405	-0.197 ±0.0460	-0.215 ±0.033 (9.7)
11	-0.235 ±0.0399	-0.399 ±0.0442	-0.302 ±0.072 (0.4)
21	-0.253 ±0.0345	-0.381 ±0.0444	-0.298 ±0.060 (2.9)

Supplementary Table 24: Real and consensus ΔG° values for multibase dangle parameters when dangles are adenines at 25°C in 1xPBS buffer.

Length (nt)	(A532) $^{(n-1)CAA}_{T}$	(ROX) $^{(n-1)CAA}_{T}$	$^{(n-1)CAA}_{T}$
1	-0.534 ±0.0498	-0.587 ±0.0529	-0.549 ±0.045 (9.0)
2	-0.433 ±0.0331	-0.500 ±0.0532	-0.446 ±0.044 (8.0)
3	-0.204 ±0.0542	-0.210 ±0.0459	-0.198 ±0.037 (9.9)
4	-0.426 ±0.0513	-0.227 ±0.0450	-0.311 ±0.072 (3.1)
6	0.018 ± 0.0524	-0.241 ±0.0388	-0.122 ±0.105 (-8.7)
9	-0.043 ±0.0364	-0.171 ±0.0407	-0.093 ±0.059 (2.6)
11	-0.104 ±0.0326	-0.156 ±0.0513	-0.112 ±0.040 (8.5)
13	-0.0377 ±0.0394	-0.289 ±0.0521	-0.135 ±0.102 (-7.6)
16	-0.1836 ±0.0478	-0.1484 ±0.0425	-0.156 ±0.032 (9.9)

Supplementary Table 25: Real and consensus ΔG° values at 25°C in 1xPBS buffer with first dangle base as adenine and rest dangles as cytosine.

Length (nt)	(A532) $^{nAA}_{T}$	(ROX) $^{nAA}_{T}$	$^{nAA}_{T}$	(A532) $^{(n-1)TAA}_{T}$	(ROX) $^{(n-1)TAA}_{T}$	$^{(n-1)TAA}_{T}$
1	-0.629 ±0.0341	-0.487 ±0.108	-0.595 ±0.080 (8.0)	-0.629 ±0.0341	-0.487 ±0.108	-0.595 ±0.080 (8.0)
2	-2.081 ±0.0628	-2.079 ±0.0592	-2.080 ±0.043 (10.0)	-0.772 ±0.0317	-0.724 ±0.0566	-0.754 ±0.037 (9.4)
3	-2.024 ±0.0381	-2.263 ±0.0720	-2.106 ±0.097 (0.6)	-0.974 ±0.0467	-0.628 ±0.0614	-0.824 ±0.129 (-10.4)
4	-1.766 ±0.0485	-1.987 ±0.0822	-1.848 ±0.094 (4.3)	-0.490 ±0.0481	-0.623 ±0.0577	-0.550 ±0.060 (6.9)
6	-1.864 ±0.0323	-1.909 ±0.0584	-1.880 ±0.037 (9.5)	-0.570 ±0.0875	-0.467 ±0.0641	-0.511 ±0.066 (9.1)
11	-2.055 ±0.0405	-2.124 ±0.0738	-2.079 ±0.049 (9.3)	-0.376 ±0.0356	-0.346 ±0.0591	-0.365 ±0.036 (9.8)
21	-2.148 ±0.0293	-2.051 ±0.0601	-2.072 ±0.040 (9.6)	-0.0144 ±0.0674	-0.0596 ±0.0564	-0.031 ±0.036 (10.0)

Supplementary Table 26: Real and consensus ΔG° values for multibase dangles in Tris-Mg buffer with first dangle base as adenine and rest dangle bases as adenine or thymine at 25°C.

Dangle type	ΔG° (kcal/mol)		ΔH° (kcal/mol)		ΔS° (cal/mol·K)	
	PBS	Tris-Mg	PBS	Tris-Mg	PBS	Tris-Mg
5’ $^{AA}_{\ \ \mathrm{T}}$	-0.77 ±0.42	-0.45 ±0.58	-5.00 ±3.07	-1.64 ±4.56	-14.18 ±8.87	-3.99 ±13.36
5’ $^{A2A}_{\ \ \ \mathrm{T}}$	-0.78 ±0.45	-1.19 ±0.61	-5.74 ±3.40	-7.84 ±4.72	-16.63 ±9.94	-22.32 ±13.81
5’ $^{A3A}_{\ \ \ \mathrm{T}}$	0.23 ±0.44	-0.61 ±0.44	1.25 ±3.30	-3.64 ±3.53	3.44 ±9.57	-10.14 ±10.36
5’ $^{A10A}_{\ \ \ \ \mathrm{T}}$	0.76 ±0.54	-0.19 ±1.10	5.44 ±3.98	-0.70 ±8.35	15.69 ±11.53	-1.73 ±24.33
5’ $^{A20A}_{\ \ \ \ \mathrm{T}}$	0.63 ±0.63	-0.59 ±0.66	4.92 ±4.77	-3.61 ±4.91	14.39 ±13.91	-10.14 ±14.25
5’ $^{TA}_{\ \ \mathrm{T}}$	-0.76 ±0.45	-1.48 ±0.43	-6.55 ±3.40	-10.22 ±3.28	-19.41 ±9.90	-29.34 ±9.57
5’ $^{T3A}_{\ \ \ \mathrm{T}}$	0.77 ±0.48	-0.51 ±0.57	5.39 ±3.53	-2.91 ±4.46	15.49 ±10.21	-8.05 ±13.07
5’ $^{T10A}_{\ \ \ \ \mathrm{T}}$	0.69 ±0.57	-1.03 ±0.77	3.50 ±4.53	-7.40 ±5.42	9.43 ±13.30	-21.35 ±15.59
5’ $^{T20A}_{\ \ \ \ \mathrm{T}}$	0.65 ±0.49	-1.50 ±0.69	3.38 ±3.66	-10.69 ±5.33	9.14 ±10.62	-30.85 ±15.54

Supplementary Table 27: Inferred thermodynamic parameters for $5'$ multinucleotide dangles at 25°C in 1x PBS buffer or Tris-Mg buffer, using melt curve analysis.

Oligo Name	Sequence	Length (nt)
5p-Ref-A	AACACT CATTCAATACCCTACGTC	24
5p-Ref-T	TACACT CATTCAATACCCTACGTC	24
5p-Ref-C	CACACT CATTCAATACCCTACGTC	24
5p-Ref-G	GACACT CATTCAATACCCTACGTC	24
5p-Comp-A	TGGATA GACGTAGGGTATTGAATG AGTGTT	30
5p-Comp-T	TGGATA GACGTAGGGTATTGAATG AGTGTA	30
5p-Comp-C	TGGATA GACGTAGGGTATTGAATG AGTGTG	30
5p-Comp-G	TGGATA GACGTAGGGTATTGAATG AGTGTC	30
5p-A532-A	/Alexa532/-AACACT CATTCAATACCCTACGTC	24
5p-A532-T	/Alexa532/-TACACT CATTCAATACCCTACGTC	24
5p-A532-C	/Alexa532/-CACACT CATTCAATACCCTACGTC	24
5p-A532-G	/Alexa532/-GACACT CATTCAATACCCTACGTC	24
5p-ROX-A	/ROX/-AACACT CATTCAATACCCTACGTC	24
5p-ROX-T	/ROX/-TACACT CATTCAATACCCTACGTC	24
5p-ROX-C	/ROX/-CACACT CATTCAATACCCTACGTC	24
5p-ROX-G	/ROX/-GACACT CATTCAATACCCTACGTC	24
5p-Catalyst	CATTCAATACCCTACGTC TATCCA TTTTTTTT	32
3p-Ref-A	CTGCATCCCATAACTTAC TCACAA	24
3p-Ref-T	CTGCATCCCATAACTTAC TCACAT	24
3p-Ref-C	CTGCATCCCATAACTTAC TCACAC	24
3p-Ref-G	CTGCATCCCATAACTTAC TCACAG	24
3p-Comp-A	TTGTGA GTA AGTTATGGGATGCAG ATAGGT	30
3p-Comp-T	ATGTGA GTA AGTTATGGGATGCAG ATAGGT	30
3p-Comp-C	GTGTGA GTA AGTTATGGGATGCAG ATAGGT	30
3p-Comp-G	CTGTGA GTA AGTTATGGGATGCAG ATAGGT	30
3p-A532-A	CTGCATCCCATAACTTAC TCACAA-/Alexa532/	24
3p-A532-T	CTGCATCCCATAACTTAC TCACAT-/Alexa532/	24
3p-A532-C	CTGCATCCCATAACTTAC TCACAC-/Alexa532/	24
3p-A532-G	CTGCATCCCATAACTTAC TCACAG-/Alexa532/	24
3p-ROX-A	CTGCATCCCATAACTTAC TCACAA-/ROX/	24
3p-ROX-T	CTGCATCCCATAACTTAC TCACAT-/ROX/	24
3p-ROX-C	CTGCATCCCATAACTTAC TCACAC-/ROX/	24
3p-ROX-G	CTGCATCCCATAACTTAC TCACAG-/ROX/	24
3p-Catalyst	TTTTTTTT ACCTAT CTGCATCCCATAACTTAC	32

Supplementary Table 28: Oligos used to determine fluorophore thermodynamics.

Oligo Name	Sequence	Length (nt)
5p-Dangle-AA	AAACACT CATTCAATACCCTACGTC	25
5p-Dangle-TA	TAACACT CATTCAATACCCTACGTC	25
5p-Dangle-CA	CAACACT CATTCAATACCCTACGTC	25
5p-Dangle-GA	GAACACT CATTCAATACCCTACGTC	25
5p-Dangle-AT	ATACACT CATTCAATACCCTACGTC	25
5p-Dangle-TT	TTACACT CATTCAATACCCTACGTC	25
5p-Dangle-CT	CTACACT CATTCAATACCCTACGTC	25
5p-Dangle-GT	GTACACT CATTCAATACCCTACGTC	25
5p-Dangle-AC	ACACACT CATTCAATACCCTACGTC	25
5p-Dangle-TC	TCACACT CATTCAATACCCTACGTC	25
5p-Dangle-CC	CCACACT CATTCAATACCCTACGTC	25
5p-Dangle-GC	GCACACT CATTCAATACCCTACGTC	25
5p-Dangle-AG	AGACACT CATTCAATACCCTACGTC	25
5p-Dangle-TG	TGACACT CATTCAATACCCTACGTC	25
5p-Dangle-CG	CGACACT CATTCAATACCCTACGTC	25
5p-Dangle-GG	GGACACT CATTCAATACCCTACGTC	25

Supplementary Table 29: Oligos used to interrogate 5′ single nucleotide dangle thermodynamics.

Oligo Name	Sequence	Length (nt)
3p-Dangle-AA	CTGCATCCCATAACTTAC TCACAAA	25
3p-Dangle-AT	CTGCATCCCATAACTTAC TCACAAT	25
3p-Dangle-AC	CTGCATCCCATAACTTAC TCACAAC	25
3p-Dangle-AG	CTGCATCCCATAACTTAC TCACAAG	25
3p-Dangle-TA	CTGCATCCCATAACTTAC TCACATA	25
3p-Dangle-TT	CTGCATCCCATAACTTAC TCACATT	25
3p-Dangle-TC	CTGCATCCCATAACTTAC TCACATC	25
3p-Dangle-TG	CTGCATCCCATAACTTAC TCACATG	25
3p-Dangle-CA	CTGCATCCCATAACTTAC TCACACA	25
3p-Dangle-CT	CTGCATCCCATAACTTAC TCACACT	25
3p-Dangle-CC	CTGCATCCCATAACTTAC TCACACC	25
3p-Dangle-CG	CTGCATCCCATAACTTAC TCACACG	25
3p-Dangle-GA	CTGCATCCCATAACTTAC TCACAGA	25
3p-Dangle-GT	CTGCATCCCATAACTTAC TCACAGT	25
3p-Dangle-GC	CTGCATCCCATAACTTAC TCACAGC	25
3p-Dangle-GG	CTGCATCCCATAACTTAC TCACAGG	25

Supplementary Table 30: Oligos used to interrogate 3′ single nucleotide dangle thermodynamics.

Oligo Name	Sequence	Length (nt)
5p-Dangle-T1A	TA AACACT CATTCAATACCCTACGTC	26
5p-Dangle-T2A	TTA AACACT CATTCAATACCCTACGTC	27
5p-Dangle-T3A	TTTA AACACT CATTCAATACCCTACGTC	28
5p-Dangle-T4A	TTTTA AACACT CATTCAATACCCTACGTC	29
5p-Dangle-T5A	TTTTTA AACACT CATTCAATACCCTACGTC	30
5p-Dangle-T6A	TTTTTTA AACACT CATTCAATACCCTACGTC	31
5p-Dangle-T8A	TTTTTTTTA AACACT CATTCAATACCCTACGTC	33
5p-Dangle-T10A	TTTTTTTTTTA AACACT CATTCAATACCCTACGTC	35
5p-Dangle-T12A	TTTTTTTTTTTTA AACACT CATTCAATACCCTACGTC	37
5p-Dangle-T15A	TTTTTTTTTTTTTTTA AACACT CATTCAATACCCTACGTC	40
5p-Dangle-T20A	TTTTTTTTTTTTTTTTTTTTA AACACT CATTCAATACCCTACGTC	45

Supplementary Table 31: Oligos used to interrogate 5′ multi-nucleotide dangles thymines with adenine (A) being the first dangle nucleotide.

Oligo Name	Sequence	Length (nt)
5p-Dangle-A1A	AA AACACT CATTCAATACCCTACGTC	26
5p-Dangle-A2A	AAA AACACT CATTCAATACCCTACGTC	27
5p-Dangle-A3A	AAAA AACACT CATTCAATACCCTACGTC	28
5p-Dangle-A5A	AAAAAA AACACT CATTCAATACCCTACGTC	30
5p-Dangle-A10A	AAAAAAAAAAA AACACT CATTCAATACCCTACGTC	35
5p-Dangle-T20A	AAAAAAAAAAAAAAAAAAAAA AACACT CATTCAATACCCTACGTC	45

Supplementary Table 32: Oligos used to interrogate 5′ multi-nucleotide dangles adenines next to the first dangle nucleotide adenine.

Oligo Name	Sequence	Length (nt)
5p-Dangle-C1A	CA AACACT CATTCAATACCCTACGTC	26
5p-Dangle-C2A	CCA AACACT CATTCAATACCCTACGTC	27
5p-Dangle-C3A	CCCA AACACT CATTCAATACCCTACGTC	28
5p-Dangle-C5A	CCCCCA AACACT CATTCAATACCCTACGTC	30
5p-Dangle-C8A	CCCCCCCCA AACACT CATTCAATACCCTACGTC	33
5p-Dangle-C10A	CCCCCCCCCCA AACACT CATTCAATACCCTACGTC	35
5p-Dangle-C12A	CCCCCCCCCCCCA AACACT CATTCAATACCCTACGTC	37
5p-Dangle-C15A	CCCCCCCCCCCCCCCA AACACT CATTCAATACCCTACGTC	40

Supplementary Table 33: Oligos used to interrogate 5′ multi-nucleotide dangles cytosines (C) next to adenine as the first dangle nucleotide.

Oligo Name	Sequence	Length (nt)
5p-Dangle-T1T	TT AACACT CATTCAATACCCTACGTC	26
5p-Dangle-T2T	TTT AACACT CATTCAATACCCTACGTC	27
5p-Dangle-T3T	TTTT AACACT CATTCAATACCCTACGTC	28
5p-Dangle-T4T	TTTTT AACACT CATTCAATACCCTACGTC	29
5p-Dangle-T5T	TTTTTT AACACT CATTCAATACCCTACGTC	30
5p-Dangle-T6T	TTTTTTT AACACT CATTCAATACCCTACGTC	31
5p-Dangle-T7T	TTTTTTTT AACACT CATTCAATACCCTACGTC	32
5p-Dangle-T9T	TTTTTTTTTT AACACT CATTCAATACCCTACGTC	34
5p-Dangle-T11T	TTTTTTTTTTTT AACACT CATTCAATACCCTACGTC	36
5p-Dangle-T14T	TTTTTTTTTTTTTTT AACACT CATTCAATACCCTACGTC	39
5p-Dangle-T19T	TTTTTTTTTTTTTTTTTTTT AACACT CATTCAATACCCTACGTC	44

Supplementary Table 34: Oligos used to interrogate 5′ multi-nucleotide dangles as thymines.

Oligo Name	Sequence	Length (nt)
5p-Dangle-T1C	TC AACACT CATTCAATACCCTACGTC	26
5p-Dangle-T2C	TTC AACACT CATTCAATACCCTACGTC	27
5p-Dangle-T3C	TTTC AACACT CATTCAATACCCTACGTC	28
5p-Dangle-T4C	TTTTC AACACT CATTCAATACCCTACGTC	29
5p-Dangle-T5C	TTTTTC AACACT CATTCAATACCCTACGTC	30
5p-Dangle-T6C	TTTTTTC AACACT CATTCAATACCCTACGTC	31
5p-Dangle-T8C	TTTTTTTTC AACACT CATTCAATACCCTACGTC	33
5p-Dangle-T10C	TTTTTTTTTTC AACACT CATTCAATACCCTACGTC	35
5p-Dangle-T12C	TTTTTTTTTTTTC AACACT CATTCAATACCCTACGTC	37
5p-Dangle-T15C	TTTTTTTTTTTTTTTC AACACT CATTCAATACCCTACGTC	40
5p-Dangle-T20C	TTTTTTTTTTTTTTTTTTTTC AACACT CATTCAATACCCTACGTC	45

Supplementary Table 35: Oligos used to interrogate 5′ multi-nucleotide dangles T with C being the first dangle nucleotide.

Oligo Name	Sequence	Length (nt)
5p-Dangle-T1G	TG AACACT CATTCAATACCCTACGTC	26
5p-Dangle-T2G	TTG AACACT CATTCAATACCCTACGTC	27
5p-Dangle-T3G	TTTG AACACT CATTCAATACCCTACGTC	28
5p-Dangle-T4G	TTTTG AACACT CATTCAATACCCTACGTC	29
5p-Dangle-T5G	TTTTTG AACACT CATTCAATACCCTACGTC	30
5p-Dangle-T6G	TTTTTTG AACACT CATTCAATACCCTACGTC	31
5p-Dangle-T8G	TTTTTTTTG AACACT CATTCAATACCCTACGTC	33
5p-Dangle-T10G	TTTTTTTTTTG AACACT CATTCAATACCCTACGTC	35
5p-Dangle-T12G	TTTTTTTTTTTTG AACACT CATTCAATACCCTACGTC	37
5p-Dangle-T15G	TTTTTTTTTTTTTTTG AACACT CATTCAATACCCTACGTC	40
5p-Dangle-T20G	TTTTTTTTTTTTTTTTTTTTG AACACT CATTCAATACCCTACGTC	45

Supplementary Table 36: Oligos used to interrogate 5′ multi-nucleotide dangles T with G being the first dangle nucleotide.

Oligo Name	Sequence	Length (nt)
3p-Dangle-AT1	CTGCATCCCATAACTTAC TCACAA AT	26
3p-Dangle-AT2	CTGCATCCCATAACTTAC TCACAA ATT	27
3p-Dangle-AT3	CTGCATCCCATAACTTAC TCACAA ATTT	28
3p-Dangle-AT4	CTGCATCCCATAACTTAC TCACAA ATTTT	29
3p-Dangle-AT5	CTGCATCCCATAACTTAC TCACAA ATTTTT	30
3p-Dangle-AT6	CTGCATCCCATAACTTAC TCACAA ATTTTTT	31
3p-Dangle-AT8	CTGCATCCCATAACTTAC TCACAA ATTTTTTTT	33
3p-Dangle-AT10	CTGCATCCCATAACTTAC TCACAA ATTTTTTTTTT	35
3p-Dangle-AT12	CTGCATCCCATAACTTAC TCACAA ATTTTTTTTTTTT	37
3p-Dangle-AT15	CTGCATCCCATAACTTAC TCACAA ATTTTTTTTTTTTTTT	40
3p-Dangle-AT20	CTGCATCCCATAACTTAC TCACAA ATTTTTTTTTTTTTTTTTTTT	45

Supplementary Table 37: Oligos used to interrogate 3′ multi-nucleotide dangles T with A being the first dangle nucleotide.

Oligo Name	Sequence	Length (nt)
3p-Dangle-TT1	CTGCATCCCATAACTTAC TCACAA TT	26
3p-Dangle-TT2	CTGCATCCCATAACTTAC TCACAA TTT	27
3p-Dangle-TT3	CTGCATCCCATAACTTAC TCACAA TTTT	28
3p-Dangle-TT4	CTGCATCCCATAACTTAC TCACAA TTTTT	29
3p-Dangle-TT5	CTGCATCCCATAACTTAC TCACAA TTTTTT	30
3p-Dangle-TT6	CTGCATCCCATAACTTAC TCACAA TTTTTTT	31
3p-Dangle-TT7	CTGCATCCCATAACTTAC TCACAA TTTTTTTT	32
3p-Dangle-TT9	CTGCATCCCATAACTTAC TCACAA TTTTTTTTTT	34
3p-Dangle-TT11	CTGCATCCCATAACTTAC TCACAA TTTTTTTTTTTT	36
3p-Dangle-TT14	CTGCATCCCATAACTTAC TCACAA TTTTTTTTTTTTTTT	39
3p-Dangle-TT19	CTGCATCCCATAACTTAC TCACAA TTTTTTTTTTTTTTTTTTTT	44

Supplementary Table 38: Oligos used to interrogate 3′ multi-nucleotide dangles thymines.

Oligo Name	Sequence	Length (nt)
3p-Dangle-CT1	CTGCATCCCATAACTTAC TCACAA CT	26
3p-Dangle-CT2	CTGCATCCCATAACTTAC TCACAA CTT	27
3p-Dangle-CT3	CTGCATCCCATAACTTAC TCACAA CTTT	28
3p-Dangle-CT4	CTGCATCCCATAACTTAC TCACAA CTTTT	29
3p-Dangle-CT5	CTGCATCCCATAACTTAC TCACAA CTTTTT	30
3p-Dangle-CT6	CTGCATCCCATAACTTAC TCACAA CTTTTTT	31
3p-Dangle-CT8	CTGCATCCCATAACTTAC TCACAA CTTTTTTTT	33
3p-Dangle-CT10	CTGCATCCCATAACTTAC TCACAA CTTTTTTTTTT	35
3p-Dangle-CT12	CTGCATCCCATAACTTAC TCACAA CTTTTTTTTTTTT	37
3p-Dangle-CT15	CTGCATCCCATAACTTAC TCACAA CTTTTTTTTTTTTTTT	40
3p-Dangle-CT20	CTGCATCCCATAACTTAC TCACAA CTTTTTTTTTTTTTTTTTTTT	45

Supplementary Table 39: Oligos used to interrogate 3′ multi-nucleotide dangles T with C being the first dangle nucleotide.

Oligo Name	Sequence	Length (nt)
3p-Dangle-GT1	CTGCATCCCATAACTTAC TCACAA GT	26
3p-Dangle-GT2	CTGCATCCCATAACTTAC TCACAA GTT	27
3p-Dangle-GT3	CTGCATCCCATAACTTAC TCACAA GTTT	28
3p-Dangle-GT4	CTGCATCCCATAACTTAC TCACAA GTTTT	29
3p-Dangle-GT5	CTGCATCCCATAACTTAC TCACAA GTTTTT	30
3p-Dangle-GT6	CTGCATCCCATAACTTAC TCACAA GTTTTTT	31
3p-Dangle-GT8	CTGCATCCCATAACTTAC TCACAA GTTTTTTTT	33
3p-Dangle-GT10	CTGCATCCCATAACTTAC TCACAA GTTTTTTTTTT	35
3p-Dangle-GT12	CTGCATCCCATAACTTAC TCACAA GTTTTTTTTTTTT	37
3p-Dangle-GT15	CTGCATCCCATAACTTAC TCACAA GTTTTTTTTTTTTTTT	40
3p-Dangle-GT20	CTGCATCCCATAACTTAC TCACAA GTTTTTTTTTTTTTTTTTTTT	45

Supplementary Table 40: Oligos used to interrogate 3′ multi-nucleotide dangles T with G being the first dangle nucleotide.

Supplementary note 1: Motif thermodynamics inferred from traditional melt analysis

Thermal melt analysis is traditionally the most commonly used method to determine DNA thermodynamic parameters. The study of the melting characteristics of DNA oligomers as means to evaluate sequence-dependent thermodynamic stability of DNA was first reported in the early 1980's. Two assumptions are applied during the analysis: (1) the transition equilibrium is assumed to involve only two states, and (2) the difference in the heat capacities of the two states is assumed to be negligible.

In melting analysis, samples are heated at a constant rate from 4°C to 95°C. The duplex to coil transition is monitored by measuring the absorbance at 260 nm, or by fluorescence of an intercalating dye. The upper and lower temperature baselines were assumed to represent a single state: either duplex or single strand. Because the duplex and single-stranded states themselves may exhibit temperature-dependent absorbance/fluorescence, slopes must be fitted to determine hybridization yields at other temperatures. Subjective judgement calls in baseline slope determination may have significant impact on the inferred ΔG° values.

A rough description of a typical melt analysis procedure is as follows: Two short complementary single strands form duplex D: S1 + S2 $\rightleftharpoons$ D. The parameters $\Delta H^\circ_{\text{duplex}}$ and $\Delta S^\circ_{\text{duplex}}$ are the duplex melting transition enthalpy and entropy, respectively. The total concentration of strands C_T is given by C_T=[S1] + [S2] + 2[D]. At $\tau = \tau_m$, $C_T K_D = \alpha$, where $\alpha = 4$ when S1 and S2 are distinct species or $\alpha = 1$ when S1 and S2 are identical. From $\Delta H^\circ_{\text{duplex}}$-$\tau_m \cdot \Delta S^\circ_{\text{duplex}} = R \cdot \tau_m \ln(C_T/\alpha)$, the enthalpy and entropy can be obtained by two methods: (1) averaging ΔH° and ΔS° from the fits of individual curves, and (2) fitting plots of reciprocal melting temperature versus the natural logarithm of the total strand concentration.

One shortcoming for melting approach is that the two-state assumption is frequently incorrect for moderate to long DNA oligonucleotides, because the single-stranded molecules may adopt one or more secondary structures. For any sequence, unless two-state melting is independently verified, uncertainties due to potential deviations from two-state behavior will always affect the predictions of melting stability. Because of this, most previous reports used short oligonucleotides (6-16nt) or self-complementary strands.

Melt experiments do not produce very accurate thermodynamic parameters for several reasons. First, experiments are typically performed in 1 M NaCl, possibly for historical reasons. Thermodynamics parameters fitted at one buffer condition are difficult to extrapolate to other buffer conditions; for example, to date there is no salt adjustment for the thermodynamics of RNA folding. Additionally, assumptions about the baselines in melt curve analysis affect the inferred ΔH° and ΔS° values. Finally, although DNA absorbs light strongly at 260 nm wavelength, other chemical impurities in the solution may also absorb at this wavelength, complicating inference of DNA hybridization thermodynamics.

Even disregarding the challenges of extrapolating thermodynamic parameters from melting analysis to other temperatures and buffer conditions, melt analysis must contend with a decomposition problem, because the fitted ΔH° and ΔS° values represent the sum of all component motifs (e.g. base stacks). For example, Bommarito's studies on single-nucleotide dangles used 9 nt oligonucleotides, so that the ΔH° and ΔS° values obtained correspond to the sum of 7 base stacks and 2 single-base dangles. Through the course of roughly 30 such experiments, a system of linear equations can be set up to solve for the ΔG° of each single-base dangle. However, this approach lacks accuracy due to the error contributions by the 7 individual base stack parameters for each sequence.

Temperature-dependence of ΔH° and ΔS°. In melt curve analyses, ΔH° and ΔS° of hybridization are assumed to be temperature invariant, which is equivalent to the assumptio of no change in heat capability Cp. Published experimental data, however, have been inconsistent with regards to this assumption. Whereas ΔCp has been correlated with changes in solvent-exposed hydrophobic surface area, the underlying physical basis of this correlation lies in changes in the fluctuations of hydrogen-bonding patterns among solvating waters. Based on this, Cp should be temperature dependent. Several groups have performed research on heat capacity change of DNA across temperatures using numerous different approaches [2-4]. This is the case for the data analyzed by Petrushka and Goodman [5], who found strong nonlinear correlations between experimental values of ΔH° and ΔS° for regular base-pair doublets and a number of mismatched and modified base pairs. A significant heat capacity increase ΔCp associated with DNA melting, in the range of 40-100 cal/mol·K per base pair was also reported in ref. [6].

Should the ΔH° and ΔS° of DNA hybridization motifs truly be temperature dependent, previous thermodynamic parameters inferred from melt curves would be even further challenged for accuracy. Our native characterization approach, in contrast, allows more direct measurement of ΔH° and ΔS° at temperatures far away from

melting temperature.

Supplementary note 2: Noncovalent Catalysis vs. Thermal Anneal

The reader may wonder if a thermal anneal of the reaction shown in manuscript Fig. 1b could be forced to equilibrium quick through a thermal anneal process. This would potentially simplify the thermodynamics measurement protocol, because no catalyst oligonucleotide would be needed. However, the authors believe that thermal annealing would lead to a kinetic trap and result in inaccurate results.

At lower temperatures, the dissociation rate duplexes become exponentially slower, so the system essentially get trapped in the equilibrium distribution at the melting temperature of the most stable species. Changes in the relative ΔG° of different species at lower temperatures will not be reflected in the observed species distribution. Thus, thermal anneal does NOT guarantee equilibrium species distribution, in contrast to popular opinion.

To take a concrete hypothetical example, assume XZ +Y $\Longrightarrow$ YX +X has $\Delta G^\circ_{\mathrm{rxn}} = +1.43$ kcal/mol at 55°C. This means there are 9:1 of YZ and XZ. As temperature falls to 25°C, assume $\Delta G^\circ_{\mathrm{rxn}}$ becomes +2.72 kcal/mol, and it is supposed to have a redistribution of YZ and XZ in the ratio of 99:1. However, dissociation rates of both YZ and XZ are so slow that the species are still trapped at the condition of 55 °C, leading to observation of YZ and XZ being still 9:1. One way to solve this problem is to spend exponentially more time at lower temperatures to reach equilibrium, but this approach then degenerates into the naive slow equilibration approach shown in Fig. 1b.

Use of the noncovalent catalyst is thus a better solution for accurate native characterization of DNA hybridization motif thermodynamics.

Supplementary note 3: Characterization of oligonucleotide stoichiometry

Historically and typically, the extinction coefficient ϵ of a DNA oligonucleotide at 260 nm is calculated via a nearest-neighbor model, and the concentration of an oligonucleotide is calculated by dividing the observed absorbance A by ϵ and path length l via the Lambert-Beer Law ($c = \frac{A}{\epsilon \cdot l}$). However, absorption characterization of concentration is not ideal because (1) chemical buffers and impurities can contribute to absorbance at 260 nm, and (2) predicted extinction coefficient ϵ based on literature parameters deviate from true values by up to 15% (based on the authors' experience). Such inaccuracies in concentrations and stoichiometry would have distinct impact on the inferred ΔG° of DNA thermodynamic motifs.

In this paper, we arbitrarily assume that the extinction coefficient-based concentrations of Complement (bottom) strands are nominally correct, and measure the concentrations of the Reference (top) strands based on native polyacrylamide gel electrophoresis (PAGE). Our methodology is demonstrated in Supplementary Figure 9.

The stoichiometry PAGE allows us to determine the relative concentrations of reference and test species to their paired complementary species, but does not give the absolute concentrations of any species. Fortunately, the absolute concentrations of oligonucleotide species are not needed for our native characterization methods of inferring motif thermodynamics. For example, in a typical $T + PC \rightleftharpoons TC + P$ reaction, $\Delta G^\circ = -R\tau \ln(K_{\mathrm{eq}}) = -R\tau \ln(\frac{[TC][P]}{[T][PC]})$ will be the same if the concentrations of all species are scaled.

Supplementary note 4: Suitability of fluorescent PAGE assays for thermodynamic characterization

We believe that fluorescent PAGE is a good method for assaying reaction equilibrium, because PAGE separates DNA species and provides identity information as well as confirmation that there are no undesirable intermediates, and because fluorescent PAGE has extremely low background signal that enables accurate yield quantitation. To further ensure accurate quantitation, we picked the ROX and Alexa-532 fluorophores because they are highly photostable.

Low background. We observed lower background in scans of PAGE gels with fluorescent species, as compared to SybrGold stained PAGE gels. SybrGold and other intercalating dyes preferentially bind to dsDNA, but also nonspecifically binds to the gel matrix and furthermore has a baseline fluorescence even when it is not intercalated. This leads to background fluorescence, which needs to be subtracted to infer band intensity, causing an increase in band intensity quantitation error.

Fluorophore photostability. We typically let the catalysis reactions proceed at the desired temperatures for up to 3 hours to ensure equilibration (under foil to minimize photobleaching), and subsequently run the gel for

up to 90 minutes. Verification that photobleaching is not significant at these time scales is important to ensure reproducibility of results, as well as to ensure that there do not exist systematic biases in converting fluorescent band intensity to concentration. Supplementary Figure 13 shows that even with 5.5 hr of continuous excitation at 45 °C, there is not significant reduction of fluorescence for either the Alexa-532 or ROX fluorophores.

Supplementary note 5: Fluorophore $\Delta G^\circ_{\text{rxn}}$ inference from band intensities

In this section, we describe how quantitated gel bands are used to infer $\Delta G^\circ_{\text{rxn}}$ values. Other than the 6-lane gel shown in manuscript Fig. 2b demonstrating the effectiveness of catalysis, all other gels include only the 4 lanes corresponding to lanes 1, 2, 5, and 6 in Fig. 2b, omitting lanes 3 and 4 because these pre-equilibrium states do not help inform ΔG°.

Supplementary Figure 14 illustrates the process by which band intensities are first mapped to concentrations, and then to ΔG° values. Importantly, the per-unit fluorescence of single-stranded species and double-stranded species are not assumed to be identical, because of the known quenching effects of proximal G nucleotides. Furthermore, there is lane-to-lane pipetting error, which results in a difference of total DNA quantity in each lane; the total inferred concentration of the fluorescence species in each lane is corrected according to the total inferred concentration in the first lane.

Systematic bias due to CZ, CXZ, and CYZ. We did not observe any higher molecular weight CXZ or CYZ bands on any of the fluorescent PAGE experiments. However, we cannot quantitate the equilibrium concentration of CZ (a.k.a. Cat·Comp), and the presence of significant CZ at equilibrium will result in an overestimate of reaction ΔG°. From our analysis (Supplementary Figure 16), we estimate that there is a systematic bias of no more than 0.2 kcal/mol due to CZ. Furthermore, we believe that this systematic bias will be roughly the same in value for all experiments involving fluorophores. The upshot is that dangle parameter characterizations that subtract two different ΔG° values likely have little to no bias due to CZ intermediates.

Fitting ΔH° and ΔS° values. From the 4 different best-fit values of ΔG° at 10, 25, 37, and 45°C, we can make a preliminary estimate of ΔH° and ΔS° based on maximum likelihood fits, described in Supplementary Figure 17. Fitted values of ΔH° and ΔS° will be reported in later supplementary sections.

Placing confidence intervals on ΔH° and ΔS° tends to be more subtle than that of ΔG°, due to the fact that the 4 ΔG° values may have different standard deviations resulting in asymmetrical upper and lower bounds relative to the best-fit. In particular, placing a reasonable estimate on ΔS° given such few temperature-based data points was difficult, so we arbitrarily picked a methodology to provide a rough estimate. Finally, we generally do not believe that ΔH° and ΔS° are temperature invariant, which means that our linear fits may not be of high predictive value for extrapolating ΔG° values at other temperatures.

Effects of Running buffer. Our hybridization and catalysis reactions were performed in 10 mM Tris-$MgCl_2$ buffer or 1x PBS buffer at different temperatures. Our PAGE assays were subsequently carried out in 1x TAE running buffer at the same temperature as the hybridization/catalysis reaction. Samples were immediately loaded, and voltage was typically applied within 3 minutes of loading. We beleve that equilibriums were not significantly disturbed due to this transient buffer change.

To experimentally verify that buffer switching does not impact inferred thermodynamic paramters, we performed four groups of experiments with 1x TAE with 12.5 mM $MgCl_2$ as running buffer, and compared them to gels run in TAE without $MgCl_2$. Supplementary Figure 18 shows that there is no significant difference in inferred ΔG° values for either fluorophores or single-base dangles, at the p = 0.05 (2 standard deviation) significance level. We could not practically use 1x PBS as gel running buffer because the high salinity would cause exorbiant amounts of Joule heating. Based on our results using TAE with Mg^{2+} as running buffer, we expect that parameters in 1x PBS are likewise not significantly affected by the transient change in gel running buffer before application of voltage.

Species identity confirmation via SybrGold. A single stranded ladder 10-60 bases and a double strand ladder were used to support the identities of the gel bands we used for equilibrium constant calculation.

Supplementary note 6: Single-base dangle $\Delta G^\circ_{\text{rxn}}$ inference

As described in the main text, the ΔG° of a single-base dangle is arithmetically calculated by subtracting the ΔG° values of two reactions. The first reaction reports the ΔG° of a fluorophore next to a duplex, and the second reaction reports the ΔG° of a fluorophore next to a duplex minus the dangle thermodynamics. In this and future

sections, we also refer to the ΔG° of the second reaction as the "raw" ΔG° value, and the subtracted ΔG° value corresponding to only the dangle as the "real" ΔG° value. We performed all experiments using both ROX and Alexa-532 fluorophores, so there are two sets of raw and real ΔG° values for each dangle parameter. The two real ΔG° values are then combined into a single consensus ΔG° value as described in the main text. Supplementary Figure 20 shows the workflow of the experiments we performed to obtain the 160 consensus ΔG° values for the 32 single-base dangles under 5 different sets of temperature/salinity conditions.

We previously described the positive bias that ignoring the CZ intermediate would have on inferred ΔG° values, but argued that this effect would be canceled out for dangles because both reaction 1 and reaction 2 will harbor similar systematic biases. Supplementary Figure 21 shows the effect of different concentrations of CZ on the inferred dangle ΔG° value. As the assumed concentration of CZ ranges between 0 and 30 nM (the total amount of catalyst in the reaction), the ΔG° of both reaction 1 and reaction 2 become more negative by about 0.2 kcal/mol. The deviation in the dangle ΔG°, however, is kept below 0.1 kcal/mol.

This analysis assumes that the concentration of CZ is identical or similar for both reaction 1 and reaction 2, which is likely to be true because the sequence identity of the CZ species is the same in both reaction 1 and 2, implying the same standard free energy of formation.

Supplementary note 7: Melt curve analysis

To compare our method to traditional melt curve analysis, we also performed inference of ΔG° for poly A and poly T multinucleotide dangles using traditional melting analysis and Van't Hoff equation fitting. The concentrations of DNA strands and hybridization buffer used was identical to those in our native catalysis experiments. Table S11-1 shows the summary of the melt curve based results; in 1x PBS there is a trend for longer dangles to be destabilizing, but this is not present in TE-$MgCl_2$ buffer. This is consistent with our observations from native catalysis fluorescent PAGE experiments. Note that the error bars on ΔG° values are significantly higher for melt curve experiments.

Supplementary Figure 70 demonstrates the overall procedure for ΔH° and ΔS° inference from melt curves. The observed fluorescence at different temperatures (blue) is compared against a linear fit of the fluorescence dependence temperature at low temperatures (red) and at high temperatures (purple). This allows inference of hybridization yields at different temperatures; the equilibrium constant K is calculated from the hybridization yield. The Van't Hoff plot shows the relationship between the natural logarithm of the equilibrium constant K and the inverse of the temperature (in Kelvin). The best linear fit to the Van't Hoff plot gives the ΔH° (slope) and ΔS° (intercept) of the hybridization reaction. Mathematically, the fitting of the Van't Hoff plot can be expressed as:

$$K = \frac{[dsDNA]}{[ssDNA]*[ssDNA]} \quad (1)$$

$$\Delta G^\circ = \Delta H^\circ - \tau \cdot \Delta S^\circ \quad (2)$$

$$\Delta G^\circ = -R \cdot \tau \cdot \ln(K) \quad (3)$$

$$\ln(K) = \frac{-\Delta H^\circ}{R} \cdot \frac{1}{\tau} + \frac{\Delta S^\circ}{R} \quad (4)$$

The details of the upper and lower baselines can have large impact on the inferred thermodynamic parameters. For example, Supplementary Figure 71 shows the effects of slight differences in assumption in determining the upper and lower baselines. For our melt curve thermodynamics inferences, we consistently used the average of the first 7 data points (60 to 61.2 °C) to fit the upper baseline and 20 data points (71.2 to 75 ^circC) to fit the lower baseline. Different assumptions here would have led to systematically different ΔH° and ΔS° values.

Each set of data was acquired in triplicate, and ΔG°, ΔH° and ΔS° values were averaged from triplicates. As mentioned in the main text and manuscript Fig. 2fg, melt curves showed poor reproducibility in the Van't Hoff plots, resulting in errors that increase the standard deviation of the best estimate ΔG° of dangle motifs. These run-to-run errors are presumably unbiased and can be corrected through averaging a statistically large number of experiments, but the 6- to 12-fold higher standard deviation over the native catalysis method means that 36- to 144-fold more experiments are needed to achieve the same parameter precision.
